# A Novel 10‐Protein Score for Liver Fat Content Predicts Cardiovascular‐Kidney‐Metabolic Disease Risk

**DOI:** 10.1002/advs.202515645

**Published:** 2025-12-23

**Authors:** Xiaoqin Gan, Yuanxiu Wei, Yiting Wu, Xinyue Su, Gangling Wang, Sisi Yang, Ziliang Ye, Yanjun Zhang, Hao Xiang, Yu Huang, Yiwei Zhang, Yuanyuan Zhang, Xianhui Qin

**Affiliations:** ^1^ Division of Nephrology Nanfang Hospital Southern Medical University National Clinical Research Center for Kidney Disease State Key Laboratory of Multi‐organ Injury Prevention and Treatment Guangdong Provincial Institute of Nephrology Guangdong Provincial Key Laboratory of Renal Failure Research Guangzhou 510515 China; ^2^ Department of Epidemiology and Biostatistics School of Public Health Anhui Medical University Hefei Anhui China; ^3^ Institute of Biomedicine Anhui Medical University Hefei China

**Keywords:** cardiovascular‐kidney‐metabolic disease, liver fat content, magnetic resonance imaging, proteomics, proton density fat fraction

## Abstract

Liver fat content (LFC) is a key biomarker for cardiovascular‐kidney‐metabolic (CKM), yet noninvasive assessment remains challenging. It aims to develop a plasma proteomic‐based LFC score to predict MRI‐derived proton density fat fraction (MRI‐PDFF), evaluate its association with 14 CKM diseases, and explore interactions with polygenic risk scores (PRS). Both the comprehensive 62‐protein and simplified 10‐protein LFC scores are developed and validated in 5,320 UK Biobank participants (96.8% White). The 10‐protein LFC score, comprising proteins involved in adipogenesis, lipid metabolism, and insulin signaling (IGFBP2, FABP4, MET, CPM, CES1, IGFBP1, CDHR2, RBP5, ERBB2, SSC5D), showed superior diagnostic accuracy for hepatic steatosis compared to fatty liver index(FLI) (AUC = 0.850 vs. 0.794). Among 45,444 participants, it exhibited significant associations with 16 cardiometabolic risk markers and 13 incident CKM outcomes, particularly metabolic dysfunction‐associated steatotic liver disease (MASLD), type 2 diabetes (T2D), chronic kidney disease, and coronary heart disease. The score significantly enhanced risk prediction for 8–10 CKM outcomes beyond standard risk factors and FLI (e.g., MASLD,C‐index improved from 0.743 to 0.774; T2D, from 0.773 to 0.806). The score showed significant interactions with PRS for T2D (*P*‐interaction<0.001). The 62‐protein model yielded similar results. A 10‐protein score accurately quantifies liver fat, predicts cardiometabolic risk, and integrates with genetic risk for precision prevention, providing a practical MRI alternative.

## Graphical abstract

A novel 10‐protein plasma score precisely quantifies liver fat, outperforming the fatty liver index. It robustly predicts the risk of numerous cardiovascular‐kidney‐metabolic diseases and integrates with genetic data for precision prevention, offering a practical alternative to magnetic resonance imaging (MRI).

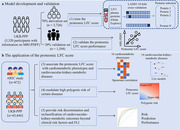

## 1 Introduction

Liver fat content (LFC) is a key prognostic biomarker closely linked to cardiovascular and metabolic health.^[^ ^1^, ^2^ ^]^ As the primary indicator of hepatic steatosis, accurate LFC quantification is clinically essential for diagnosing and managing metabolic dysfunction‐associated steatotic liver disease (MASLD, formerly termed non‐alcoholic fatty liver disease [NAFLD]), a condition defined by concurrent hepatic steatosis and cardiometabolic risk factors according to recent international consensus guidelines.^[^ ^3^ ^]^ Although liver biopsy remains the historical gold standard for diagnosis, its invasiveness poses substantial limitations, including sampling variability, low patient acceptance for serial monitoring, and procedure‐related risks.^[^ ^4^ ^]^ These constraints have accelerated the development of non‐invasive approaches for LFC assessment.

The advent of magnetic resonance imaging‐derived proton density fat fraction (MRI‐PDFF) has revolutionized LFC quantification by enabling noninvasive, whole‐liver fat assessment. As the current gold standard for noninvasive imaging, MRI‐PDFF offers excellent accuracy and reproducibility for LFC measurement.^[^ ^5^ ^]^ However, its clinical adoption faces challenges due to high costs, specialized equipment needs, long scan times, and incompatibility with metallic implants. These limitations have driven the search for accessible biomarkers that can accurately reflect MRI‐PDFF‐measured LFC, which would facilitate early steatosis detection and cost‐effective monitoring.

Plasma proteomics offers a powerful platform for high‐throughput biomarker discovery and risk stratification.^[^ ^6^, ^7^, ^8^ ^]^ Previous studies have identified circulating proteins associated with fatty liver disease or metabolic dysfunction‐associated steatohepatitis (MASH)/MASLD,^[^ ^9^, ^10^, ^11^, ^12^, ^13^, ^14^ ^]^ including regulators of adipogenesis and fat deposition (e.g., IGFBP2,^[^ ^15^, ^16^ ^]^ CES1,^[^ ^17^ ^]^ IGFBP1^[^ ^18^, ^19^ ^]^), modulators of insulin sensitivity (e.g., FABP4,^[^ ^20^ ^]^ IGFBP1), and mediators of lipid metabolism (e.g., MET,^[^ ^21^ ^]^ IGFBP2). However, most prior efforts have prioritized diagnosis of advanced disease stages, particularly MASH/MASLD with significant fibrosis,^[^ ^10^, ^11^, ^12^, ^13^, ^14^ ^]^ leaving proteomic signatures specific to early hepatic steatosis largely unexplored.

To address these gaps and leverage the precision of MRI‐PDFF while overcoming its practical limitations, we sought to identify plasma protein biomarkers that not only correlate closely with imaging‐based liver fat quantification but also provide predictive value for clinical outcomes. Currently, a significant research void exists in large‐scale proteomic investigations directly linked to MRI‐PDFF‐quantified liver fat. The development of proteomic signatures that accurately reflect LFC while simultaneously predicting cardiovascular‐kidney‐metabolic (CKM) outcomes (here referring to the spectrum of conditions interconnecting cardiovascular, metabolic, and renal systems) could revolutionize preventive strategies for metabolic disorders. Such an integrated approach would not only enhance early detection of hepatic steatosis but also help elucidate the mechanistic pathways connecting hepatic fat accumulation to systemic metabolic dysregulation.

Therefore, we utilized comprehensive proteomic data from the UK Biobank Pharma Proteomics Project (UKB‐PPP)^[^ ^22^ ^]^ to achieve three key objectives: 1) develop and validate a plasma proteomic‐derived LFC score for predicting hepatic steatosis (MRI‐PDFF>5%); 2) assess its clinical relevance by examining associations with diverse cardiometabolic traits and evaluating its predictive performance for 14 CKM outcomes; and 3) explore potential interactions between the proteomic signature and polygenic risk scores (PRS) for cardiometabolic diseases (including hypertension, coronary heart disease [CHD], and type 2 diabetes [T2D]) to elucidate gene‐protein‐disease pathways.

## 2 Results

### 2.1 Study Population and Baseline Characteristics

The initial analysis comprised UK Biobank participants with available MRI‐PDFF and proteomics data, who were randomly allocated into derivation (70%, *N* = 3726) and validation (30%, *N* = 1594) cohorts (Figure S1, Supporting Information). As presented in **Table** **1**, both cohorts demonstrated comparable baseline characteristics.

**Table 1:** General characteristics of study participants.

Characteristics^a)^	Derivation dataset	Validation dataset	Proteomic dataset
N	3726	1594	45444
Age, year	55.0 (48.0,61.0)	54.0 (48.0,60.0)	57.0 (49.0,63.0)
Male, n (%)	1773 (47.6)	752 (47.2)	20107 (44.2)
White, n (%)	3604 (96.7)	1545 (96.9)	42627 (93.8)
BMI, kg m^−2^	25.9 (23.5,28.6)	26.1 (23.6,28.8)	26.5 (24.0,29.5)
TDI	−2.6 (‐3.8, ‐0.2)	−2.5 (−3.9, −0.2)	−2.1 (−3.7,0.6)
SBP, mmHg	132.5 (121.5145.0)	133.0 (121.5144.5)	135.5 (124.0148.5)
Smoking status, n (%)			
Never	2228 (59.8)	974 (61.1)	25507 (56.1)
Previous	1266 (34.0)	513 (32.2)	15219 (33.5)
Current	232 (6.2)	107 (6.7)	4718 (10.4)
Alcohol drinking, n (%)			
Never	177 (4.8)	74 (4.6)	3554 (7.8)
<1 time/week	707 (19.0)	288 (18.1)	10040 (22.1)
1‐4 times/week	1977 (53.1)	884 (55.5)	22553 (49.6)
>4 times/week	865 (23.2)	348 (21.8)	9297 (20.5)
LDL‐C, mmol/L	3.5 (3.0,4.0)	3.6 (3.0,4.0)	3.6 (3.0,4.1)
HbA1c, mmol/L	34.9 (32.2,36.9)	34.6 (32.2,36.6)	35.3 (32.8,37.4)

^a)^ Data are expressed as median (IQR), or n (%), accordingly.

Abbreviations: BMI, body mass index; HbA1c, glycosylated hemoglobin A1c; SBP, systolic blood pressure; TDI, Townsend deprivation index; LDL‐C, low‐density lipoprotein cholesterol.

The association between the proteomic LFC score and risk of 14 CKM outcomes was evaluated using a dataset excluding participants with prevalent CKM diseases (N = 45444; median age 57.0 years; 44.2% male; 93.8% white; Figure S1, Supporting Information). Compared with participants in the derivation and validation sets, those in the proteomic dataset were older, more likely to be female, or smokers. Additionally, they were observed to reside in more deprived areas and to have higher systolic blood pressure and HbA1c levels (Table 1).

### 2.2 Development and Validation of the Proteomic LFC Scores (both the Comprehensive [62‐Protein] Score and Simplified 10‐Protein Score)

**Figure 1:**
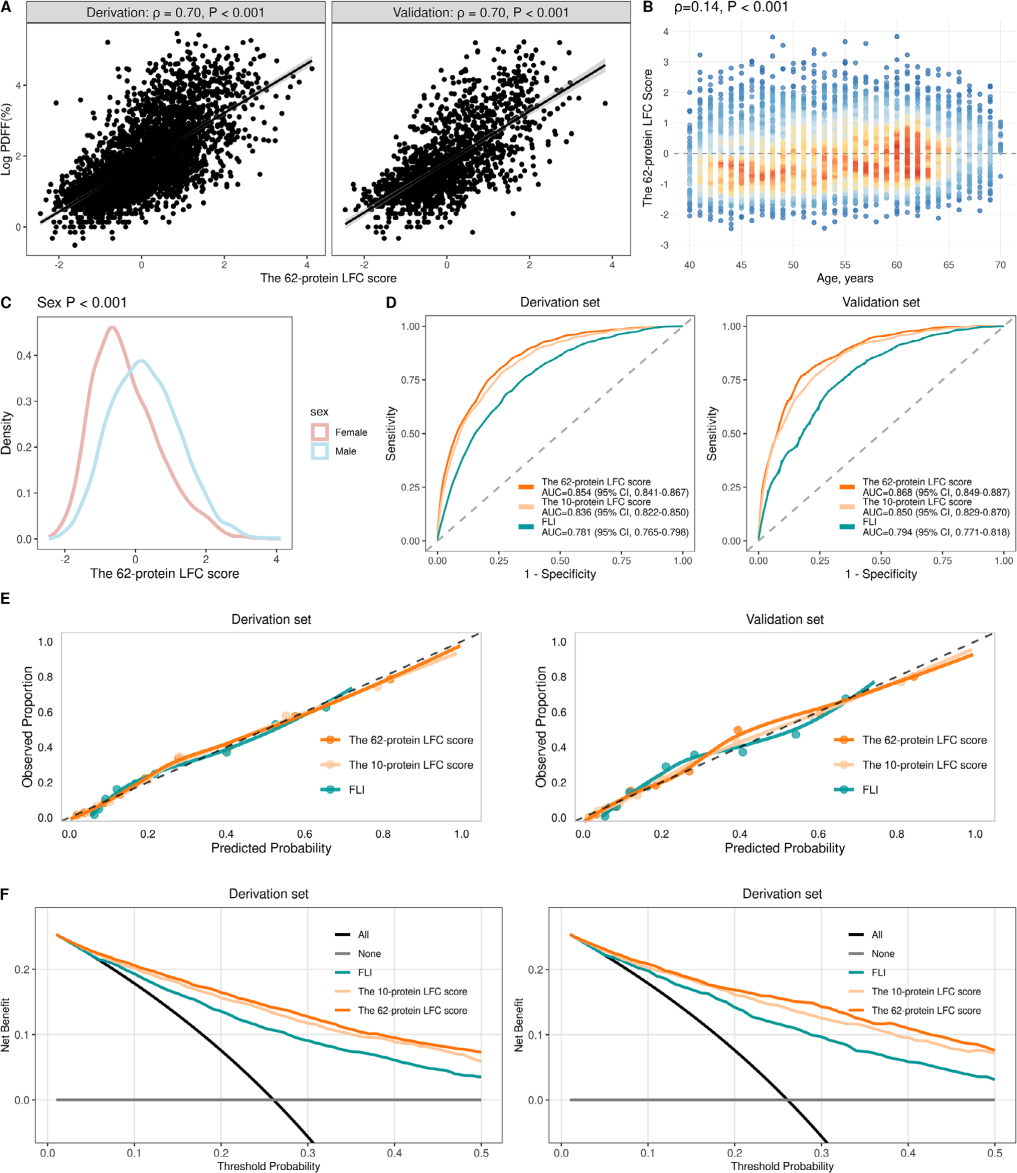
Development and validation of the proteomic LFC score. A) Correlation between the proteomic LFC score and MRI‐PDFF in the derivation (left, n = 3726) and validation (right, *n* = 1594) cohorts. B,C) Association of the proteomic LFC score with (B) age and (C) sex (*n* = 5320). D) Receiver operating characteristic (ROC) analysis for predicting hepatic steatosis (MRI‐PDFF >5%) in derivation (*n* = 3726) and validation (*n* = 1594) cohorts. E) Calibration plots demonstrating the accuracy of probability predictions, with the dashed line representing perfect calibration in derivation (*n* = 3726) and validation (*n* = 1594) cohorts. F) Decision curve analysis (DCA) evaluating the net clinical benefit of the models across different threshold probabilities in derivation (*n* = 3726) and validation (*n* = 1594) cohorts. *P* values in (A) and (B) were derived from Spearman rank correlation tests; *P* values in (C) were obtained from linear regression models assessing the relationship between the proteomic LFC score and sex. Abbreviations: ROC, receiver operating characteristic; MRI, magnetic resonance imaging; PDFF, proton density fat fraction; LFC, liver fat content; FLI, fatty liver index.

The comprehensive proteomic LFC score was developed through LASSO regression analysis in the derivation cohort, incorporating 62 of 2911 plasma proteins (Figure S2 and Table S1, Supporting Information), achieving a >97% reduction in proteomic dimensionality. This score demonstrated strong correlations with MRI‐PDFF values in both derivation and validation cohorts (Spearman's ρ = 0.70 for both, *P* < 0.001; **Figure** **1**A). Consistent with established LFC epidemiology,^[^ ^23^ ^]^ the score showed positive associations with age (Figure 1B) and was significantly higher in male participants (Figure 1C). The comprehensive 62‐protein LFC score demonstrated significantly higher AUC values for diagnosing hepatic steatosis (MRI‐PDFF >5%) than the FLI in both the derivation (0.854 vs. 0.781; *P* < 0.001) and validation (0.868 vs. 0.794; *P* < 0.001) sets (Figure 1D; Table S2, Supporting Information).

To improve clinical utility, we developed a simplified 10‐protein score based on the highest absolute LASSO coefficients. The 10‐protein model was found to offer the optimal balance, achieving 97.9% of the maximal discriminative accuracy (AUC = 0.836 vs. 0.854 for the full 62‐protein model) while substantially improving parsimony and clinical feasibility (Figure S3, Table S1, Supporting Information). Both AIC and BIC supported this choice, as a clear performance plateau was observed beyond 10 proteins (Figure S3, Table S1, Supporting Information). Bootstrap resampling (*n* = 500) confirmed the high stability of the simplified 10‐protein panel. Eight of the ten proteins were selected in over 90% of the bootstrap iterations, and the mean selection frequency for the entire panel was 86.4% (Figure S4, Supporting Information). This abbreviated 10‐protein score maintained strong correlations with both MRI‐PDFF (Spearman's ρ = 0.66 in both cohorts, *P* < 0.001) and the comprehensive proteomic score (Spearman's ρ = 0.97 in both cohorts, *P* < 0.001). The 10‐protein LFC score demonstrated significantly superior discrimination for diagnosing hepatic steatosis (MRI‐PDFF >5%) compared with the FLI, with AUCs of 0.836 vs. 0.781 in the derivation set and 0.850 vs. 0.794 in the validation set (both *P* < 0.001), despite its substantially reduced panel size (Figure 1D; Table S2, Supporting Information).

For continuous liver fat quantification, both proteomic models demonstrated substantially better performance than FLI (Table S2, Supporting Information). The 10‐protein model achieved superior accuracy (R^2^: 0.28–0.30; RMSE: 4.07–4.09%; MAE: 2.55–2.56%) compared to FLI (R^2^: 0.18‐0.22; RMSE: 4.32–4.36%; MAE: 2.65–2.66%) across derivation and validation sets, indicating its superior ability to capture the continuous information inherent in MRI‐PDFF measurements.

All three models, the 62‐protein LFC score, 10‐protein LFC score, and FLI, exhibited good calibration in both derivation and validation sets (Figure 1E), with calibration slopes and intercepts consistently near the ideal values (slope = 1, intercept = 0; Table S2, Supporting Information). The 62‐protein LFC score achieved the lowest Brier scores (derivation: 0.130; validation: 0.125), indicating superior overall performance, followed by the 10‐protein score (derivation: 0.137; validation: 0.131) and FLI (derivation: 0.156; validation: 0.153; Table S2, Supporting Information). DCA revealed comparable clinical utility between the two protein‐based scores, both of which provided higher net benefit than FLI across a wide range of threshold probabilities (Figure 1F). GO and KEGG enrichment analyses indicated that the 62 LFC‐related plasma proteins were significantly enriched in several key biological pathways, including steroid, peptide, and glucocorticoid hormone responses, lipid transport and localization, and the PPAR signaling pathway within the endocrine system (Figure S5, Table S3, Supporting Information). The 10‐protein panel (IGFBP2, FABP4, MET, CPM, CES1, IGFBP1, CDHR2, RBP5, ERBB2, SSC5D) comprehensively reflects the multifactorial pathogenesis of hepatic steatosis and its systemic metabolic consequences by targeting different interconnected key pathways (Table S4, Supporting Information).^[^ ^15^, ^16^, ^17^, ^18^, ^19^, ^20^, ^21^, ^24^, ^25^, ^26^, ^27^, ^28^, ^29^ ^]^

### 2.3 Associations of Comprehensive 62‐Protein and 10‐protein LFC Scores and Their Top Ten Constituent Proteins with Cardiometabolic Risk Markers

**Figure 2:**
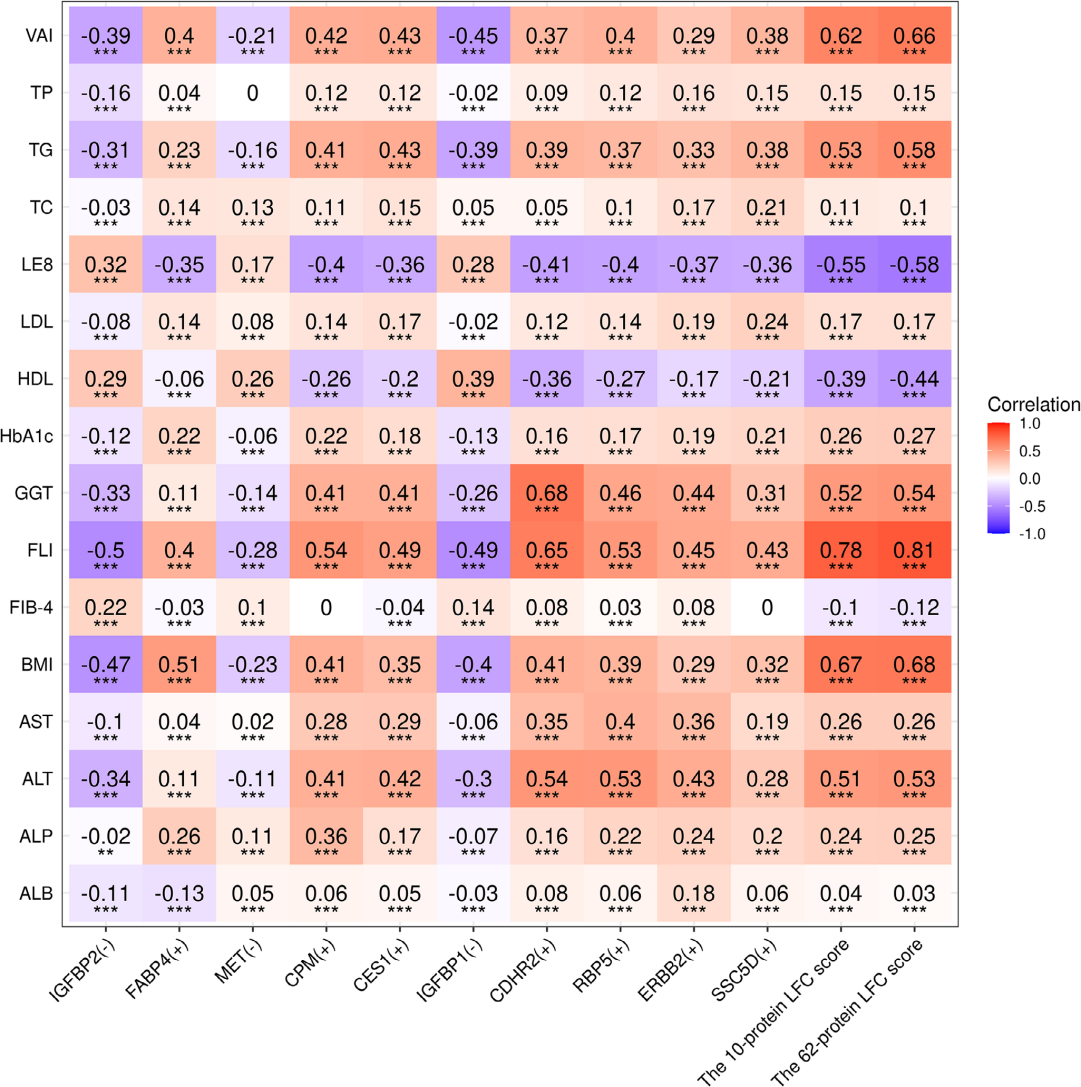
Associations of comprehensive (62‐protein) and 10‐protein LFC scores and their top ten constituent proteins with cardiometabolic risk markers and hepatic biomarkers (*n* = 45444). An (+) or (‐) mark in proteins indicates the LASSO directionality. Statistical significance was assessed using Spearman rank correlation tests, as indicated by ^*^ *P* < 0.05, ^**^ *P* < 0.01, and ^***^ *P* < 0.001. Abbreviations: LFC, liver fat content; TG, triglyceride; TC, total cholesterol; HDL‐C, high‐density lipoprotein‐cholesterol; LDL‐C, low‐density lipoprotein‐cholesterol; HbA1c, glycosylated hemoglobin A1c; BMI, body mass index; VAI, visceral adiposity index; LE8, life's essential 8; ALT, alanine aminotransferase; AST, aspartate aminotransferase; GGT, gamma‐glutamyl transferase; ALP, alkaline phosphatase; ALB, albumin; TP, total protein; FLI, fatty liver index; FIB‐4, fibrosis‐4 index; LASSO, least absolute shrinkage and selection operator.

In the proteomic dataset (*n* = 45444, Figure S1, Supporting Information), both the 62‐protein and 10‐protein LFC scores showed significant associations with key cardiometabolic risk factors and hepatic biomarkers. The scores demonstrated: 1) strong positive correlations (all ρ > 0.6) with VAI, BMI, and FLI; 2) moderate positive associations (all ρ > 0.5) with TG and ALT levels; and 3) moderate inverse correlations (both ρ < −0.5) with LE8 scores (**Figure** **2**). Notably, neither score showed an association with the FIB‐4, aligning with its established role as a marker of hepatic fibrosis rather than steatosis. Proteins with positive LASSO directionality exhibited correlations with cardiometabolic phenotypes in the same direction as the proteomic LFC score, while those with negative directionality showed opposite correlations.

To preliminarily assess the generalizability of our proteomic findings, we conducted a cross‐platform consistency analysis using data from the multi‐ethnic Atherosclerosis Risk in Communities (ARIC) study (covering 461 proteins in a subsample of *n* = 472 participants at visit 5, 2011‐2013).^[^ ^30^ ^]^ Despite differences in proteomic profiling technology (SOMAscan vs. Olink) and the limited overlap of proteins, three overlapping proteins (FABP4, MET, and CES1) demonstrated consistent cross‐sectional associations with key cardiometabolic risk factors (Figure S6, Supporting Information). This observation suggests that the key biological signals captured by our panel may be robust across distinct measurement platforms and populations.

### 2.4 Associations Between Comprehensive 62‐Protein and Simplified 10‐Protein LFC Scores and 14 CKM Outcomes Risks

During a median follow‐up of 13.6 years (IQR: 12.8–14.3) in 45444 participants without prevalent CKM diseases, 2757 incident T2D cases (6.1%), 11919 hypertension cases (26.2%), 3821 CHD cases (8.4%), 563 MASLD cases (1.2%), and 1722 CKD cases (3.8%) were identified. Information on other outcomes was documented in Table S5 (Supporting Information).

**Figure 3:**
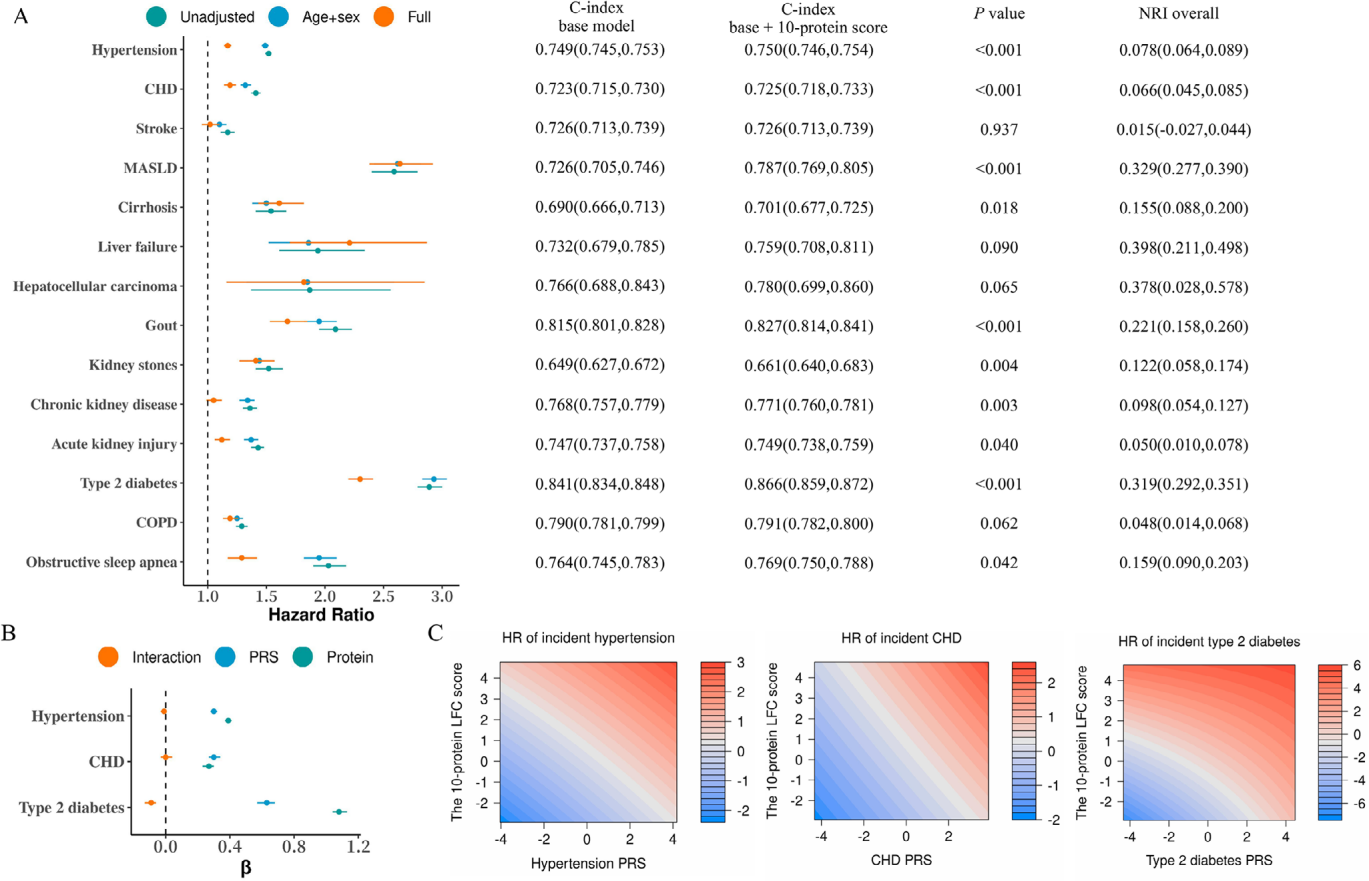
Association of the 10‐protein LFC score and polygenic risk with 14 cardiovascular‐kidney‐metabolic (CKM) outcomes (*n* = 45444). A) Forest plot of Cox regression analyses showing associations between the 10‐protein LFC score and 14 CKM outcomes. The fully adjusted model incorporated standard risk factors: age, sex, race, BMI, Townsend deprivation index, smoking status, alcohol use, systolic blood pressure, LDL cholesterol, and HbA1c. Error bars indicate 95% CIs. The accompanying table compares model discrimination (C‐index) between baseline models (standard risk factors only) and enhanced models (adding the 10‐protein score), with *P* values reflecting the significance of predictive improvement. *P* values for the difference in the C‐index were obtained using bootstrap tests with 100 replications. B) Cox regression coefficients (β) with 95% confidence intervals (error bars) derived from models testing interactions between the 10‐protein LFC score and PRSs of hypertension, CHD, and T2D on corresponding incident outcomes. C) Contour map of the model‐predicted HR across the range of proteomic LFC score and PRSs, with median values as reference points. Abbreviations: LFC, liver fat content; COPD, chronic obstructive pulmonary disease; BMI, body mass index; MASLD, metabolic dysfunction‐associated steatotic liver disease; CHD, coronary heart disease; HbA1c, glycosylated hemoglobin A1c; LDL‐C, low‐density lipoprotein‐cholesterol; PRS, polygenic risk scores.

After multivariable adjustment for standard risk factors (including age, sex, race, BMI, Townsend deprivation index, smoking, alcohol, systolic blood pressure, LDL‐C, and HbA1c), each 1‐SD increase in the simplified 10‐protein LFC score showed significant associations with 13 CKM outcomes: hypertension (adjusted HR, 1.15; 95%CI, 1.12–1.18), CHD (1.16; 1.11–1.21), MASLD (2.38; 2.15–2.64), cirrhosis (1.53; 1.36–1.72), liver failure (2.05; 1.58–2.67), hepatocellular carcinoma (1.86; 1.19–2.90), gout (1.70; 1.54–1.87), kidney stones (1.38; 1.24–1.53), CKD (1.18; 1.11‐1.25), AKI (1.11; 1.05–1.18), T2D (2.19; 2.09–2.30), COPD (1.12; 1.06–1.19), and OSA (1.33; 1.20–1.46) (**Figure** **3**A; Table S5, Supporting Information). Restricted cubic spline analyses confirmed that while the associations demonstrated some non‐linearity, they maintained a predominantly monotonically increasing relationship for the 13 significant outcomes (Figure S7, Supporting Information). This pattern strengthens the evidence for a positive dose‐response relationship while revealing greater complexity in the risk trajectories. The effect estimates remained consistent between the comprehensive 62‐protein LFC score and the simplified 10‐protein score across nearly all outcomes (Figure S8A, Table S5, Supporting Information).

Notably, the 62‐ and 10‐protein LFC scores showed a significant association with stroke in crude and age‐/sex‐adjusted models, which was attenuated to non‐significance upon further adjustment for cardiometabolic risk factors. Nevertheless, stratified analyses identified a significant interaction by age (both *P*‐interaction < 0.001). Specifically, a significant risk increase was observed exclusively in participants under 57 years old (10‐protein; adjusted HR, 1.17; 95% CI, 1.05–1.32; 62‐protein; adjusted HR, 1.21; 95% CI, 1.07–1.35) (Table S6, Supporting Information).

The associations of the 62‐ and 10‐protein LFC scores with disease outcomes proved robust and persistent across all sensitivity analyses, encompassing multiple imputation, complete‐case analysis, competing risk adjustment, and propensity score methods (Table S7, Supporting Information).

### 2.5 Discriminative Performance and Reclassification Improvement of Comprehensive 62‐Protein and Simplified 10‐Protein LFC Scores for 14 CKM Outcomes

The simplified 10‐protein LFC score demonstrated significant improvements in both discrimination and reclassification beyond standard risk factors for 10 CKM outcomes (all *P* < 0.05). Notable enhancements included: MASLD (C‐index improved from 0.726 to 0.787; Continuous NRI = 32.9%), T2D (from 0.841 to 0.866; NRI = 31.9%), gout (from 0.815 to 0.827; NRI = 22.1%), cirrhosis (from 0.690 to 0.701; NRI = 15.5%), CHD (from 0.723 to 0.725; NRI = 6.6%), and CKD (from 0.768 to 0.771; NRI = 9.8%) (Figure 3B).

The integration of the 10‐protein LFC score with FLI models yielded enhanced predictive performance for 8 outcomes, with particularly strong improvements observed for: MASLD (C‐index improved from 0.743 to 0.774; NRI = 22.7%), liver failure (from 0.697 to 0.719; NRI = 24.4%), T2D (from 0.773 to 0.806; NRI = 26.3%), and gout (from 0.815 to 0.822; NRI = 14.3%). More modest but statistically significant improvements were seen for CHD (from 0.712 to 0.714; NRI = 5.5%) and CKD (from 0.749 to 0.752; NRI = 7.1%) (**Table** **2**).

**Table 2:** Enhanced predictive performance of the 10‐protein LFC score for 14 cardiovascular‐kidney‐metabolic (CKM) outcomes beyond FLI (*n* = 45444).

Outcomes	C‐index		*P* value	NRI overall
	Age +sex+ FLI	Age +sex + FLI + 10‐protein score		
Hypertension	0.701(0.696,0.705)	0.703(0.699,0.708)	<0.001	0.060(0.045,0.071)
CHD	0.712(0.704,0.720)	0.714(0.706,0.721)	0.010	0.055(0.027,0.076)
Stroke	0.708(0.694,0.721)	0.708(0.694,0.721)	0.933	−0.037(−0.054,0.087)
MASLD	0.743(0.723,0.763)	0.774(0.755,0.793)	<0.001	0.227(0.160,0.288)
Cirrhosis	0.664(0.640,0.689)	0.669(0.644,0.693)	0.170	0.103(0.056,0.163)
Liver failure	0.697(0.644,0.750)	0.719(0.668,0.770)	0.045	0.244(0.087,0.364)
Hepatocellular carcinoma	0.750(0.656,0.843)	0.753(0.660,0.845)	0.802	0.147(−0.130,0.381)
Gout	0.815(0.801,0.829)	0.822(0.808,0.836)	<0.001	0.143(0.094,0.194)
Kidney stones	0.631(0.608,0.653)	0.645(0.623,0.667)	0.002	0.101(0.048,0.158)
Chronic kidney disease	0.749(0.738,0.761)	0.752(0.741,0.763)	0.006	0.071(0.036,0.103)
Acute kidney injury	0.721(0.710,0.732)	0.722(0.711,0.733)	0.106	0.040(0.003,0.072)
Type 2 diabetes	0.773(0.764,0.781)	0.806(0.798,0.814)	<0.001	0.263(0.227,0.285)
COPD	0.685(0.674,0.695)	0.685(0.674,0.696)	0.313	0.027(−0.025,0.047)
Obstructive sleep apnea	0.742(0.723,0.761)	0.744(0.726,0.763)	0.236	0.052(0.000,0.097)

*P* values for the difference in the C‐index were obtained using bootstrap tests with 100 replications.

Abbreviations: LFC, liver fat content; FLI, fatty liver index; COPD, chronic obstructive pulmonary disease; MASLD, metabolic dysfunction‐associated steatotic liver disease; CHD, coronary heart disease; NRI, net reclassification improvement.

Categorical NRI analysis provided further insight into the patterns of risk reclassification across different outcomes (Tables S8 and S9, Supporting Information). The 10‐protein LFC score showed robust overall NRI improvements when added to either standard risk factors or FLI models for incident MASLD (overall NRI = 10.7–12.4%) and T2D (overall NRI = 6.4%). Notably, distinct reclassification patterns emerged between these conditions: when added to FLI, the NRI improvement for MASLD was primarily driven by better identification of future cases (event NRI = 8.2%), whereas for T2D, the improvement mainly resulted from more accurate reclassification of non‐events (non‐event NRI = 4.3%).

The comprehensive protein LFC score demonstrated similar predictive improvements (Figure S8B, Tables S8–S10, Supporting Information). Sensitivity analyses on both multiply imputed and complete‐case datasets reproduced the primary findings, confirming that the predictive improvement of the 62‐ and 10‐protein LFC scores for a range of CKM outcomes was consistent and robust beyond the FLI and established risk factors (Tables S11–S14, Supporting Information).

Furthermore, when directly compared against the FLI, both proteomic LFC scores demonstrated significantly superior predictive performance for multiple incident conditions, including MASLD, kidney stones, and type 2 diabetes (all Delong's *P* < 0.05). For hypertension, CKD, AKI, and OSA, the protein‐based scores showed slightly lower but largely comparable discrimination to FLI, while performance showed no significant difference for all other endpoints (Figure S9, Table S15, Supporting Information). When evaluated against the imaging‐based reference standard, MRI‐PDFF, both proteomic scores exhibited significantly higher predictive accuracy for four key CKM outcomes: incident hypertension, CHD, CKD, and T2D (all *P* < 0.05). For all other outcomes assessed, the proteomic models achieved performance metrics that were statistically comparable to those of MRI‐PDFF itself (Figure S9, Table S16, Supporting Information).

### 2.6 Integration of Comprehensive 62‐Protein and Simplified 10‐Protein LFC Scores and Polygenic Risk on Incident Outcomes

Both analytical approaches, evaluating PRS as a continuous variable with formal interaction terms and stratifying by PRS tertiles, consistently demonstrated significant interactions between the proteomic LFC scores and polygenic risk for T2D (all *P*‐interaction < 0.02), but not for hypertension or CHD (all *P*‐interaction > 0.10) (Figure 3B; Figures S8B and S10, Table S17, Supporting Information). When PRS was analyzed as a continuous variable, the interaction with T2D PRS yielded an HR of 0.91 for both proteomic scores (*P* < 0.001) (Table S17, Supporting Information), indicating that the proteomic score's association with T2D risk modestly attenuated as genetic risk increased. This attenuation pattern was visually confirmed in the stratified analysis (Figure S10, Supporting Information).

Notably, participants with dual high‐risk status, characterized by elevated proteomic scores and high genetic risk, consistently exhibited the highest absolute disease hazards across all three conditions (Figure 3C; Figure S8C, Supporting Information). Moreover, for T2D, the HRs for the proteomic LFC scores were notably higher than those for PRS, highlighting the strong predictive value of the proteomic signatures (Figure 3B; Figure S8B, Table S17, Supporting Information).

## 3 Discussion

This study leverages large‐scale proteomic data from the UK Biobank to develop and validate a plasma proteomic‐derived LFC score that accurately reflects MRI‐PDFF‐measured hepatic steatosis. Our findings demonstrate that a simplified 10‐protein LFC score performs comparably to a comprehensive 62‐protein model in predicting liver fat accumulation and associated CKM outcomes. Importantly, this proteomic signature enhances risk stratification beyond conventional clinical markers and interacts with genetic predisposition, particularly for T2D. These results highlight the potential of proteomics to advance noninvasive steatosis assessment and improve early prediction of multisystem metabolic dysfunction.

Previous proteomic studies^[^ ^9^, ^10^, ^11^, ^12^, ^13^, ^14^ ^]^ have primarily focused on identifying circulating biomarkers for diagnosing advanced stages of fatty liver disease, particularly established MASH or fibrosis.^[^ ^10^, ^11^, ^12^, ^13^, ^14^ ^]^ In contrast, our study was designed to develop a proteomic signature directly aligned with the dynamic continuum of steatotic liver disease, using MRI‐PDFF as a precise and quantitative measure of LFC. This approach enabled us to uncover molecular pathways altered from the earliest phases of steatosis. Moreover, whereas earlier work has largely emphasized liver‐specific endpoints, our novel 10‐protein score demonstrated robust predictive capacity for a wide spectrum of CKM outcomes. These findings provide new, clinically relevant evidence reinforcing the role of hepatic steatosis as a sentinel marker of systemic metabolic dysregulation.

### 3.1 Proteomic Signature Reflects Liver Fat Content and Its Systemic Metabolic Context

The 62‐protein score, developed through LASSO regression, demonstrated strong correlation with MRI‐PDFF–quantified liver fat and superior discriminative performance for steatosis detection compared to the FLI (AUC = 0.868 vs. 0.794). The simplified 10‐protein score maintained robust accuracy (AUC = 0.850), supporting its potential clinical feasibility without substantial compromise in predictive power. We acknowledge that MRI‐PDFF, although the non‐invasive reference standard, is subject to technical variations, including inter‐scan variability and motion artifacts, which may attenuate the observed correlation between our proteomic score and the true hepatic fat content. Notwithstanding this limitation, the biological plausibility of our protein panel strengthens the validity of our approach. The ten selected proteins—IGFBP2, FABP4, MET, CPM, CES1, IGFBP1, CDHR2, RBP5, ERBB2, and SSC5D—collectively represent seven key interconnected pathways central to steatosis pathogenesis: 1) adipogenesis and fat deposition (IGFBP2, FABP4, CPM, CES1, IGFBP1);^[^ ^15^, ^16^, ^17^, ^18^, ^19^, ^20^, ^24^ ^]^ 2) lipid metabolism regulation (IGFBP2, MET, ERBB2);^[^ ^15^, ^16^, ^21^, ^27^ ^]^ 3) inflammation and oxidative stress (FABP4, RBP5, SSC5D);^[^ ^20^, ^28^ ^]^ 4) insulin sensitivity modulation (IGFBP2, FABP4, MET, IGFBP1, ERBB2);^[^ ^15^, ^16^, ^18^, ^19^, ^20^, ^21^, ^27^ ^]^ 5) endothelial dysfunction (FABP4);^[^ ^20^ ^]^ 6) hepatic fibrogenesis (CES1, SSC5D);^[^ ^17^, ^29^ ^]^ and 7) apoptotic regulation (CPM, IGFBP1).^[^ ^24^, ^25^ ^]^ The multi‐functional roles of several proteins (e.g., FABP4, IGFBP2, ERBB2) across different pathways underscore the interconnected biological network underlying steatosis.

The observed directional associations of these proteins with cardiometabolic traits, such as positive correlations with triglycerides and visceral adiposity, further support their relevance to steatosis‐related metabolic dysregulation. It is important to note, however, that plasma proteins originate from multiple tissues; thus, the proteomic signature likely reflects both liver‐specific processes and systemic metabolic influences. While the score was specifically designed to quantify liver fat, its biological foundation inherently encompasses the broader cardiometabolic context of MASLD. Future studies incorporating liver tissue proteomics will be essential to disentangle the liver‐specific contributions from systemic components of this signature. Similarly, further validation using complementary imaging modalities or histology could help clarify the impact of MRI‐PDFF measurement variability. Nevertheless, the current findings establish this protein‐based score as a promising tool that not only reflects liver fat content but also captures the multisystemic pathophysiological landscape of hepatic steatosis.

### 3.2 Proteomic LFC Score Predicts Diverse CKM Outcomes

Our findings establish that both the comprehensive 62‐protein and simplified 10‐protein LFC scores serve as robust predictors for a broad spectrum of CKM outcomes. The simplified 10‐protein score remained significantly associated with 13 of 14 CKM diseases following comprehensive multivariable adjustment. When evaluated against the non‐invasive imaging reference standard, MRI‐PDFF, the proteomic scores demonstrated statistically comparable performance for most endpoints and even surpassed MRI‐PDFF in predicting four key outcomes: incident hypertension, CHD, CKD, and T2D. These findings indicate that our proteomic signature serves as a measure that not only reflects the essential pathophysiological features of LFC but may also incorporate additional systemic metabolic information beyond what is captured by MRI‐PDFF alone.

Integration with the FLI further enhanced predictive accuracy, particularly for MASLD and T2D, underscoring the complementary value of combining proteomic biomarkers with established clinical markers. This synergy may stem from a fundamental distinction: while the FLI captures prevalent anthropometric and metabolic traits, our proteomic score is more likely to reflect active, pathophysiological processes that drive disease progression. The significant improvement in reclassification metrics, particularly the robust categorical net reclassification improvement, confirms that the protein panel adds clinically actionable information beyond routine clinical parameters. These findings suggest a potential role for this combined approach in refining risk stratification, especially for identifying high‐risk individuals who may benefit from targeted interventions.

Notably, the simplified 10‐protein panel retained predictive performance largely comparable to the comprehensive 62‐protein score, suggesting that a refined biomarker set can preserve substantial prognostic value while offering enhanced clinical practicality. Collectively, these results highlight the potential of plasma proteomic signatures to refine multidimensional risk assessment, particularly for cardiometabolic and hepatic outcomes, and identify opportunities for early intervention in high‐risk individuals through this integrated approach.

However, we observed that the association between the LFC score and stroke risk was notably weaker than with metabolic outcomes such as T2D and MASLD and became non‐significant after full adjustment for established metabolic risk factors. This attenuation likely reflects several interrelated factors. First, the substantial attenuation of the association after adjustment for cardiometabolic risk factors indicates that these factors largely account for the observed link between LFC and stroke. Second, the significant interaction by age further clarifies this relationship: the LFC score remained a significant risk factor only in individuals under 57 years, suggesting that its effect may be obscured in older adults by a higher burden of competing risk factors and distinct stroke mechanisms. Biologically, the LFC protein profile, enriched in pathways such as steroid hormone response and lipid transport, is more directly implicated in metabolic conditions like MASLD and T2D than in the complex, multifactorial pathophysiology of stroke. Therefore, the attenuated association with stroke underscores the primary role of the LFC protein score as a marker of systemic metabolic dysfunction, with any link to cerebrovascular events being largely indirect and modulated by age.

### 3.3 Interaction Effects with Genetic Risk

A significant interaction was observed between the proteomic LFC scores and the PRS for T2D (*P*‐interaction < 0.02), characterized by a graded attenuation pattern in which the association of the proteomic score with T2D risk was most pronounced at low genetic risk levels. This finding aligns with prior evidence highlighting the role of gene‐environment interactions in T2D development.^[^ ^31^ ^]^ Moreover, the observed graded attenuation pattern suggests heterogeneous mechanisms underlying T2D. It implies that the metabolic risk captured by the proteomic LFC scores is most discernible in the absence of a strong genetic predisposition, whereas in high‐risk individuals, this risk may be overshadowed by predominant genetic drivers. Notably, participants with combined high proteomic and high genetic risk exhibited the highest absolute disease hazards across all outcomes studied, reinforcing the additive value of integrating proteomic measures with genetic risk profiles. Furthermore, for T2D, the hazard ratios associated with the proteomic LFC scores exceeded those of the PRS, underscoring the strong independent predictive value of proteomic signatures. These findings highlight the potential of multi‐omics approaches to refine risk stratification and support the development of personalized prevention strategies that account for both inherited and dynamic metabolic determinants.

In contrast to the significant interactions observed for T2D, no substantial interactions were detected between the proteomic LFC score and polygenic risks for hypertension or CHD. We hypothesize that this discrepancy may reflect fundamental differences in both genetic architecture and the functional specificity of our proteomic signature. T2D, as a predominantly metabolic disorder, shares core pathophysiological pathways with hepatic steatosis,^[^ ^32^ ^]^ allowing our liver fat‐associated protein panel to capture essential elements of its etiology and consequently interact with its genetic determinants. In comparison, the genetic underpinnings of hypertension and CHD encompass substantial vascular, inflammatory, and renal mechanisms that may not be directly reflected by our metabolically‐oriented protein panel. These findings suggest that the interplay between proteomic and genetic risk factors is most pronounced for metabolically‐driven conditions like T2D, highlighting how multi‐omics integration can not only enhance risk stratification but also reveal the biological pathways through which different risk domains converge or operate independently.

### 3.4 Clinical Implications

Our proteomic LFC score addresses critical limitations of current liver fat assessment by providing a practical alternative to MRI‐PDFF that is suitable for broader population screening. The 10‐protein panel offers distinct translational advantages: 1) Early Detection: it enables early detection of steatosis and CKM risks before symptom onset, creating a crucial window for preventive interventions; 2) Practical Clinical Implementation: it serves as an accessible, cost‐effective first‐line screening tool that can be implemented in primary care settings, establishing a tiered clinical pathway where high‐risk individuals are efficiently triaged for confirmatory MRI or specialist referral. This approach optimizes resource allocation while overcoming the accessibility constraints of routine MRI‐PDFF screening, and 3) Personalized Risk Stratification: it provides a foundation for personalized risk stratification when integrated with genetic data, particularly for conditions like type 2 diabetes. Further steps, including assay standardization, transition to absolute quantification, health‐economic evaluations, and integration with electronic health systems, are necessary before widespread clinical implementation. Our findings underscore the viability of this protein‐based score as a scalable tool for systematic risk assessment. By complementing existing diagnostic approaches, it holds potential to improve the efficiency of specialized healthcare resource allocation. Looking ahead, the development of commercial testing panels based on this 10‐protein signature could significantly enhance accessibility in real‐world settings, though such efforts will require rigorous validation across diverse populations and careful consideration of implementation pathways.

### 3.5 Limitations

Several important limitations should be considered when interpreting our findings. First, while the proteomic platform employed in this study provides broad coverage of plasma proteins, it does not represent a complete proteome analysis, potentially missing some biologically relevant proteins involved in hepatic steatosis and its metabolic consequences. Second, despite rigorous adjustment for confounders through multivariable models, propensity score methods, and sensitivity analyses, the observational nature of our study precludes definitive causal inference. Unmeasured confounding may still influence the observed associations between plasma proteins and hepatic steatosis or CKM outcomes. Future studies using genetic instruments, such as Mendelian randomization, are warranted to clarify potential causal pathways. Third, plasma protein levels were measured at a single baseline timepoint, which does not capture potential temporal variations and may introduce non‐differential misclassification, likely leading to attenuated effect estimates. Nevertheless, from a translational perspective, this proteomic panel offers a distinct practical advantage for future longitudinal profiling: it is substantially more cost‐effective and logistically feasible than repeated MRI‐PDFF assessments. Fourth, the low number of events for certain CKM outcomes, notably liver failure, compromised our statistical power. This limitation increased the uncertainty around the risk estimates and reduced our capacity to detect modest yet clinically important associations, thereby underscoring the necessity of validation in larger cohorts. Fifth, the clinical translation of our findings will require the development and validation of absolute quantification protein assays, as the Olink PEA technology used in our study provides relative quantification (NPX values). Future work focused on establishing calibration methods to convert these relative measurements into standardized absolute concentrations will be essential for developing clinically applicable cut‐off values and facilitating implementation across different healthcare settings. Finally, while our proteomic‐derived LFC score was developed and validated within the UK Biobank, it is essential to note that this cohort is primarily composed of healthier, middle‐aged individuals of White European ancestry from less deprived areas. This specific demographic composition likely contributes to an underestimation of risk associations and may limit the direct translation of our findings to more diverse populations, particularly in light of known ethnic variations in cardiometabolic traits. Although our analysis revealed consistent associations between certain individual proteins and cardiometabolic risk factors in the multi‐ethnic ARIC study, the absence of MRI‐PDFF data and limited proteomic coverage precluded direct validation of the integrated LFC score and its association with liver fat content. Therefore, external validation of the complete proteomic score in diverse, well‐phenotyped cohorts remains an essential objective for future research. Overall, our study is hypothesis‐generating in nature, and some findings require validation in independent samples.

## 4 Conclusion

This study identifies a novel plasma proteomic signature that serves as a robust, noninvasive biomarker for hepatic steatosis and demonstrates strong predictive value for incident CKM diseases. The optimized 10‐protein model achieves an optimal balance between diagnostic accuracy and clinical practicality, while its interaction with genetic risk factors reveals important pathways for personalized prevention strategies. These findings bridge the critical gap between liver fat accumulation and systemic metabolic dysfunction, suggesting that plasma proteomics could revolutionize early risk stratification and precision medicine approaches for CKM diseases. The 10‐protein panel holds particular promise as a first‐line screening tool in primary care settings, where it could efficiently identify high‐risk individuals for subsequent confirmatory MRI assessment, thereby optimizing healthcare resource utilization and clinical efficiency.

## 5 Experimental Section

### Study Populations

The UK Biobank was a large prospective cohort comprising over 500 000 individuals aged 37–73 years, recruited from 22 assessment centers across England, Scotland, and Wales between 2006 and 2010. Participants provided extensive baseline data through questionnaires, biological samples, and physical assessments. The study was approved by the North West Research Ethics Committee (11/NW/0382), and all participants provided informed consent. Further details on the study design and data collection were available in prior publications.^[^ ^7^, ^33^, ^34^ ^]^

In the UKB‐PPP, plasma proteomic profiling was conducted for 53029 participants within the UK Biobank. Participant selection was based on a stratified, pseudo‐randomized sampling algorithm specifically designed to ensure the sub‐cohort was representative of the broader UK Biobank population, maintaining proportional distributions of age, sex, and recruitment center.^[^ ^22^ ^]^ After excluding participants who withdrew (*n* = 12), the final analytical cohort comprised 53017 individuals (Figure S1, Supporting Information). Among these, we identified 5320 participants with available MRI‐PDFF measurements, who were then randomly divided into a derivation set (*n* = 3726, 70%) and a validation set (*n* = 1594, 30%) for the development and validation of a plasma proteomic signature predicting LFC. Additionally, to assess the clinical relevance of the LFC proteomic signature, a total of 45 444 participants without prevalent CKM diseases was included in a separate proteomic dataset for outcome prediction analyses (Figure S1, Supporting Information).

### Proteomics Measurements

Proteomic profiling was performed using blood plasma samples obtained at the baseline assessment. The profiling was performed using the Olink Explore 3072 platform, which employs proximity extension assay (PEA) technology. The detailed methodology, including assay procedures, data processing pipelines, and quality control measures, has been previously described.^[^ ^22^ ^]^ This high‐throughput platform enables simultaneous quantification of protein relative abundances across four specialized panels: inflammation, oncology, cardiometabolic, and neurology. The PEA technology offers exceptional sensitivity, high reproducibility, and minimal cross‐reactivity. After rigorous quality control procedures, a total of 2941 protein analytes (representing 2923 unique proteins) were successfully quantified.

### Magnetic Resonance Imaging

Liver imaging was performed at the UK Biobank imaging center in Cheadle (UK) using a Siemens 1.5T MAGNETOM Aera scanner between August 2014 and October 2015.^[^ ^35^ ^]^ MRI‐PDFF maps were generated using a multi‐echo spoiled‐gradient‐echo acquisition. MRI‐PDFF, defined as the ratio of protons bound to fat to the total number of liver protons,^[^ ^36^ ^]^ provides a highly accurate (≈100%) quantification of LFC.^[^ ^37^ ^]^ Further details on the imaging protocol and analysis are available in prior studies,^[^ ^35^ ^]^ and the corresponding UK Biobank data field is provided in Table S18 (Supporting Information).

### Definition of PRS

In this study, we employed standardized PRS for cardiometabolic traits from the UK Biobank resource (https://biobank.ndph.ox.ac.uk/showcase/label.cgi?id=301), including hypertension, CHD, and T2D. These PRS were generated using established computational methods,^[^ ^38^ ^]^ with adjustments for principal components derived from common genotype variants in the 1000 Genomes Project reference dataset. Detailed information can be found in Table S18 (Supporting Information). All supporting data for these scores originated from external genome‐wide association studies (GWAS) as part of the UK Biobank project 9659,^[^ ^38^ ^]^ representing the standard PRS set.

### Statistical Methods*—*Missing Data Handling

During data preprocessing, we excluded 12 proteins with ≥20% missing values.^[^ ^22^ ^]^ The remaining 2911 proteins had a median missingness of 7.8% (IQR: 3.0%‐17.4%; Figure S11, Supporting Information), of which 1463 proteins showed a missing rate of less than 15%. The missing measurements of the remaining 2911 proteins were imputed with mean values.^[^ ^22^ ^]^ To assess the robustness of our findings to the choice of imputation method, we conducted a comprehensive sensitivity analysis using Multiple Imputation by Chained Equations (MICE). This imputation process assumed that missing data were missing at random (MAR) conditional on the observed variables in the dataset,^[^ ^39^, ^40^ ^]^ which was supported by our observation that missingness in the 10 proteins with the highest missingness rates was significantly associated with multiple fully observed variables (Table S19, Supporting Information). To leverage this finding, these associated variables were incorporated into the imputation model to obtain more accurate estimates. Additionally, analyses were incorporated in a complete‐case dataset comprising only subjects with no missing protein data. The impact of using a more lenient protein exclusion threshold (≥25% missingness) on our primary results was further evaluated.

### Statistical Methods—Development and Validation of Proteomic‐derived LFC score for MRI‐PDFF Prediction

Before feature selection, both the 2911 predictor proteins and MRI‐PDFF were log2‐transformed and standardized. In the derivation set, least absolute shrinkage and selection operator (LASSO) regression was applied using the R package glmnet to identify the most predictive proteins for LFC, with MRI‐PDFF as the continuous outcome. Age and sex were incorporated as mandatory, unpenalized covariates to preserve their clinical relevance in the model. The LASSO penalty parameter (λ) was optimized through 10‐fold cross‐validation, and we selected the λ.1se value (the largest λ within one standard error of the minimum mean squared error) to prioritize model parsimony while maintaining predictive accuracy. The complete derivation set, along with this cross‐validated penalty parameter, was then used in the LASSO objective function to derive the final coefficients for candidate proteins.

Cross‐validation error plots and coefficient path curves were generated to visualize the LASSO regression results and the variable selection process (Figure S2, Supporting Information). The cross‐validation plot displays the mean squared error across different penalty parameters, while the coefficient path plot illustrates how protein coefficients shrink toward zero as the penalty increases. This procedure automatically selected a 62‐protein panel through the retention of non‐zero coefficients in the final model. The proteomic‐derived LFC score was calculated as the linear combination of selected protein concentrations weighted by their respective coefficients (excluding age and sex; see Table S1, Supporting Information) and standardized (mean 0, variance 1) for subsequent analyses. Sensitivity analysis using the 25% missingness threshold (retaining 2919 proteins) yielded an identical 62‐protein panel with unchanged coefficients, confirming the stability of our feature selection.

To enhance clinical utility, a simplified proteomic‐derived LFC score was created using the top 10 proteins with the highest absolute coefficient values (Table S1, Supporting Information). A systematic model comparison was conducted across panels ranging from 1 to 62 proteins based on predictive performance (AUC), Akaike Information Criterion (AIC), and Bayesian Information Criterion (BIC) to rigorously justify the selection of 10 proteins. The stability of the simplified 10‐protein panel was assessed using bootstrap resampling with 500 iterations. The diagnostic performance of both the comprehensive (62‐protein) proteomic LFC score and the simplified 10‐protein score was then evaluated against the fatty liver index (FLI)^[^ ^41^ ^]^ for detecting hepatic steatosis (MRI‐PDFF >5%) through ROC analysis, using area under the curve (AUC) as the primary comparison metric. Pairwise comparisons of the AUC were conducted using DeLong's test. Calibration was assessed using calibration curves and quantified via the calibration slope and intercept. A slope of 1 and an intercept of 0 indicate perfect agreement between predicted probabilities and observed outcomes. Overall model performance was further using the Brier score, which measures the mean squared error between predicted probabilities and actual binary outcomes, with lower scores (ideal = 0) reflecting better performance. To assess clinical utility, decision curve analysis (DCA) was performed, estimating the net benefit of using the prediction model compared to default strategies (treat‐all or treat‐none) across a range of risk thresholds. In addition to AUC for binary classification of hepatic steatosis (MRI‐PDFF >5%), the association between the protein scores and continuous MRI‐PDFF values was evaluated using R‐squared (R^2^) to quantify explained variance, along with Root Mean Squared Error (RMSE) and Mean Absolute Error (MAE) to assess agreement.

To functionally characterize the 62 identified LFC‐related plasma proteins, we performed Gene Ontology (GO) and Kyoto Encyclopedia of Genes and Genomes (KEGG) enrichment analyses using the clusterProfiler R package. The GO analysis encompassed biological processes (BP), molecular functions (MF), and cellular components (CC). Significantly enriched terms were defined as those with a false discovery rate (FDR) <0.05. The mechanistic relevance of these 10 proteins was further investigated by examining their directionally plausible associations with key cardiometabolic parameters. These parameters included lipid profiles (triglyceride [TG], total cholesterol [TC], high‐density lipoprotein‐cholesterol [HDL‐C]), glycemic control (glycosylated hemoglobin A1c [HbA1c]), adiposity measures (body mass index [BMI], visceral adiposity index [VAI]),^[^ ^42^ ^]^ and cardiovascular health metrics (Life's Essential 8 [LE8]).^[^ ^43^, ^44^ ^]^ Additionally, their correlations with hepatic biomarkers were analyzed, encompassing liver enzymes (alanine aminotransferase [ALT], aspartate aminotransferase [AST], gamma‐glutamyl transferase [GGT]), synthetic function markers (albumin [ALB], total protein [TP]), and validated indices of hepatic steatosis (FLI) and hepatic fibrosis (FIB‐4).^[^ ^41^, ^45^, ^46^ ^]^

### Statistical Methods—Association of Proteomic LFC Scores with Various Clinical Outcomes and Interaction with Polygenic Risk

To examine the prospective relationship between proteomic LFC scores (both the comprehensive score and simplified 10‐protein score) and the risk of 14 CKM diseases, survival analyses were conducted in a cohort of 45 444 UK Biobank participants without prior diagnoses of these conditions at baseline (Figure S1, Supporting Information). The studied outcomes included hypertension, CHD, stroke, MASLD, cirrhosis, liver failure, hepatocellular carcinoma, gout, kidney stones, chronic kidney disease (CKD), acute kidney injury (AKI), T2D, chronic obstructive pulmonary disease (COPD), and obstructive sleep apnea (OSA). Disease diagnoses were ascertained using International Classification of Diseases (ICD‐9, ICD‐10) and Office of Population Censuses and Surveys (OPCS‐4) codes from hospital admissions, procedures, and operations (see Table S20, Supporting Information for specific codes). The follow‐up duration was defined as the period from enrollment (coinciding with the blood sample collection date) until the first occurrence of any of the following: disease diagnosis, death, loss to follow‐up, or the end of the study period (May 31, 2022, for Wales; August 31, 2022, for Scotland; and October 31, 2022, for England).

Associations were assessed using Cox proportional hazards models, with the proteomic LFC scores as the primary exposure. Three nested models were constructed with progressive adjustment: 1) unadjusted; 2) adjusted for age and sex; and 3) further adjusted for standard risk factors, including age, sex, race, Townsend deprivation index (TDI), BMI, smoking status, alcohol consumption, systolic blood pressure, low‐density lipoprotein cholesterol (LDL‐C), and HbA1c. Additional covariate details are provided in Table S18 (Supporting Information) and the UK Biobank protocol (www.ukbiobank.ac.uk). The proportional hazards assumption was verified using Schoenfeld residuals, with no significant violations detected across the models (Figures S12 and S13, Supporting Information). The robustness of the primary findings was evaluated through a battery of sensitivity analyses, which included: 1) multiple imputation of missing protein values; 2) complete‐case analysis using only subjects with full protein data; 3) competing risk regression via Fine‐Gray models to address mortality as a competing event; and 4) generalized propensity score (GPS) methods with both inverse probability weighting and GPS‐adjusted Cox regression to balance the full suite of clinical and demographic covariates across the protein score exposure.^[^ ^47^ ^]^

To evaluate the incremental predictive value of the proteomic LFC scores for 14 CKM diseases beyond conventional risk factors and FLI, we assessed model performance using Harrell's C‐statistic for discrimination and both continuous and categorical NRI. For categorical NRI, outcome‐specific thresholds set at half the cumulative incidence rate were employed, and the components of NRI were reported to distinguish between improved reclassification of events and non‐events. The proteomic scores were further directly compared against the imaging‐based reference standard, MRI‐PDFF, in a subset of participants with available imaging data (*n* = 5320), and against the FLI in the full proteomic cohort (*n* = 53017) (Figure S1, Supporting Information). Performance was evaluated using the AUC for discrimination and the 10‐year continuous NRI.

Cox regression was used to examine proteomic‐genetic interactions for three outcomes: 1) proteomic LFC score × hypertension PRS for incident hypertension, 2) proteomic LFC score × CHD PRS for incident CHD, and 3) proteomic LFC score × T2D PRS for incident T2D. Models included the proteomic LFC score, PRS, and an interaction term between the two, adjusted for age, sex, race, and four principal components of genetic ancestry. To enhance clinical interpretability, we generated forest plots to display the HRs of the proteomic LFC score within each genetic risk stratum by categorizing the PRS into low, intermediate, and high‐risk groups based on tertiles.

A two‐tailed *P* < 0.05 was considered to be statistically significant in all analyses. All statistical analyses were performed using R 4.1.1 software.

### Ethics Statement

The UK Biobank was approved by the North West Research Ethics Committee (11/NW/0382), and all participants signed informed consent. This research has been conducted using the UK Biobank Resource under Application Number 73201.

## Conflict of Interest

The authors declare no conflict of interest.

## Supporting information



Supporting Information

## Data Availability

The data that support the findings of this study are available from [UK Biobank]. Restrictions apply to the availability of these data, which were used under license for this study. Data are available at https://biobank.ndph.ox.ac.uk/ with the permission of [UK Biobank].
