# Supplementary material for: A Novel 10‐Protein Score for Liver Fat Content Predicts Cardiovascular‐Kidney‐Metabolic Disease Risk
Source: Adv Sci (Weinh). 2025 Dec 23;13(10):e15645. doi: 10.1002/advs.202515645 (PMC12915156; doi:10.1002/advs.202515645)
Supplement: Supplementary file 1 — Supporting Information [file ADVS-13-e15645-s001.docx]

**A Novel 10-Protein Score for Liver Fat Content Predicts Cardiovascular-Kidney-Metabolic Disease Risk**

**Supplementary Figures List**

Supplementary Figure 1. Flow chart of study participants.

Supplementary Figure 2. LASSO regression analysis for protein biomarker selection in liver fat content prediction.

Supplementary Figure 3. Model Selection Analysis for Optimal Protein Number in Liver Fat Content Prediction.

Supplementary Figure 4. Selection frequency of the 10-protein liver fat content panel across 500 bootstrap iterations.

Supplementary Figure 5. Functional Enrichment Analysis of the 62 LFC-related plasma proteins.

Supplemental Figure 6. Association of FABP4, MET, and CES1 with cardiometabolic risk factors in the Atherosclerosis Risk in Communities (ARIC) study.

Supplementary Figure 7. Restricted cubic spline curves for the associations between the simplified 10-protein LFC score and the risk of 14 CKM outcomes.

Supplementary Figure 8. Association of the 62-protein LFC score and polygenic risk with the risk of 14 cardiovascular-kidney-metabolic outcomes.

Supplementary Figure 9. The AUC for predicting cardiovascular-kidney-metabolic outcomes.

Supplementary Figure 10. Association between the 62-protein LFC score (A) and 10-protein LFC score (B) with clinical outcomes stratified by genetic risk.

Supplementary Figure 11. Distribution of missing data rates among 2,911 plasma proteins included in analysis.

Supplementary Figure 12. Schoenfeld residual plots for testing proportional hazards in the association between the 62-protein score and CKM diseases.

Supplementary Figure 13. Schoenfeld residual plots for testing proportional hazards in the association between the 10-protein score and CKM diseases.

**Supplementary Tables List**

Supplementary Table 1. Protein Weights and Model Performance for Proteomic LFC Score.

Supplementary Table 2. Comprehensive model performance for predicting hepatic steatosis (MRI-PDFF >5%) and continuous liver fat content (MRI-PDFF) in derivation and validation set.

Supplementary Table 3. Significant enrichment of the 62 LFC-related plasma proteins. Supplementary Table 4. Biological curation of top-ten LFC-related plasma proteins.

Supplementary Table 5. The association between the 62-protein LFC score and 10-protein LFC score with 14 cardiovascular-kidney-metabolic (CKM) outcomes.

Supplementary Table 6. Stratified analyses for association between the 62-protein LFC score and 10-protein LFC score with risk of stroke.

Supplementary Table 7. The association between the 62-protein LFC score and 10-protein LFC score with clinical outcomes.

Supplementary Table 8. Categorical net reclassification improvement (NRI) of the 62- and 10-protein LFC score for 14 cardiovascular-kidney-metabolic (CKM) outcomes beyond standard risk factors.

Supplementary Table 9. Categorical net reclassification improvement (NRI) of the 62- and 10-protein LFC score for 14 cardiovascular-kidney-metabolic (CKM) outcomes beyond FLI.

Supplementary Table 10. Predictive performance of the 62-protein LFC score over the FLI model for various clinical outcomes.

Supplementary Table 11. Enhanced predictive performance of the 62- and 10-protein LFC score for 14 cardiovascular-kidney-metabolic (CKM) outcomes beyond FLI using multiple imputation.

Supplementary Table 12. Enhanced predictive performance of the 62- and 10-protein LFC score for 14 cardiovascular-kidney-metabolic (CKM) outcomes beyond standard risk factors using multiple imputation.

Supplementary Table 13. Enhanced predictive performance of the 62- and 10-protein LFC score for 14 cardiovascular-kidney-metabolic (CKM) outcomes beyond FLI: Analysis of Complete Cases (Sample size: 41,213 for the 10-protein score and 26,168 for the 62-protein score).

Supplementary Table 14. Enhanced predictive performance of the 62- and 10-protein LFC score for 14 cardiovascular-kidney-metabolic (CKM) outcomes beyond standard risk factors: Analysis of Complete Cases (Sample size: 41,213 for the 10-protein score and 26,168 for the 62-protein score).

Supplementary Table 15. Predictive performance of protein panels versus FLI for various disease outcomes.

Supplementary Table 16. Predictive performance of protein panels versus PDFF for various disease outcomes.

Supplementary Table 17. Cox model results from UK Biobank with protein scores and polygenic risk scores as the main predictors, including an interaction term.

Supplementary Table 18. Definitions of relevant UKB data fields and codes.

Supplementary Table 19. MAR Assumption Evaluation Results.

Supplementary Table 20. Disease definitions used in the UK Biobank study.


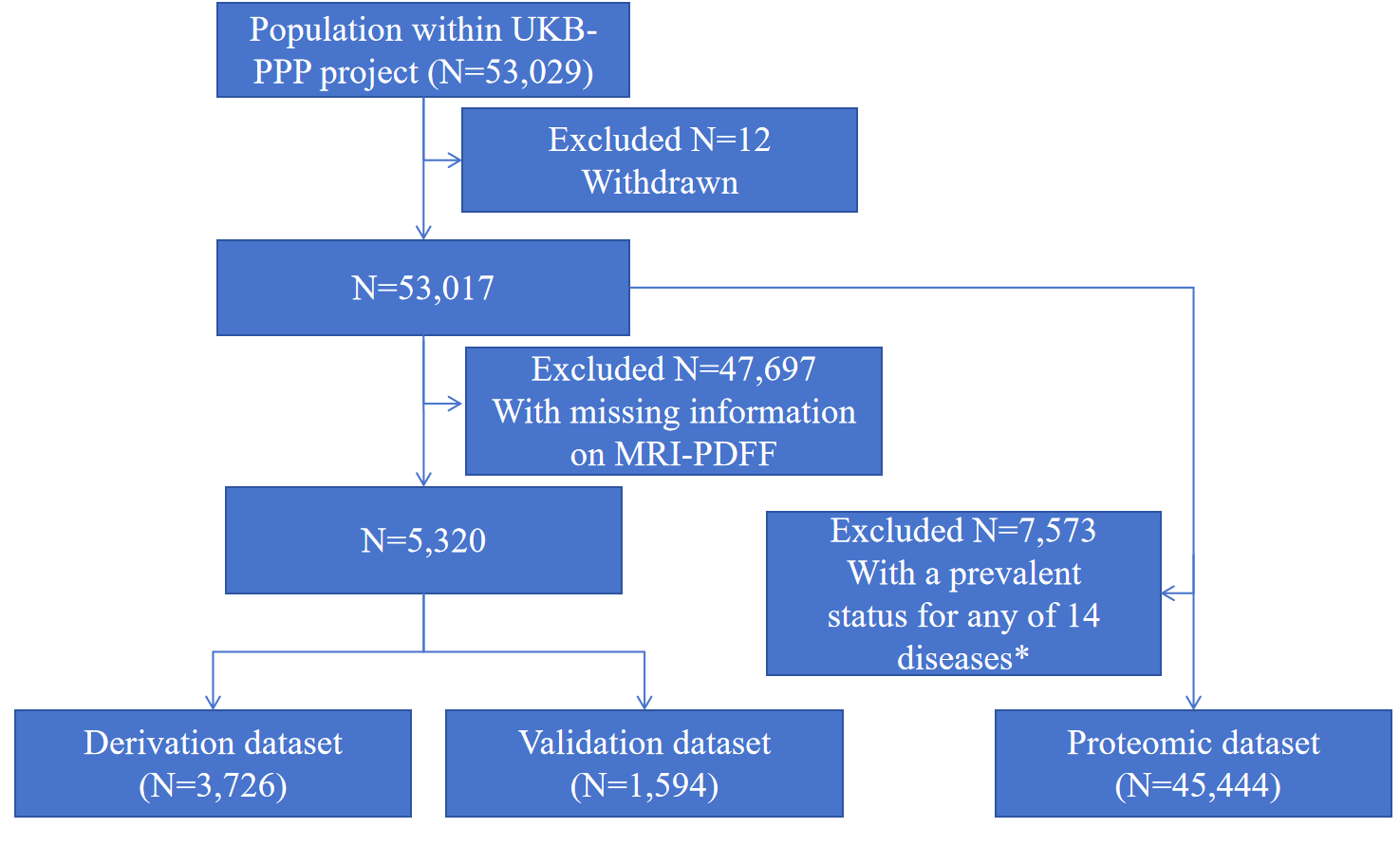


**Supplementary Figure 1. Flow chart of study participants.**

*These 14 diseases included hypertension, coronary heart disease, stroke, MASLD, cirrhosis, liver failure, hepatocellular carcinoma, gout, kidney stones, chronic kidney disease, acute kidney injury, type 2 diabetes, COPD and obstructive sleep apnea.

**Abbreviation:** MRI-PDFF, magnetic resonance imaging-based proton density fat fraction; UKB-PPP, UK Biobank Pharma Proteomics Project.

**
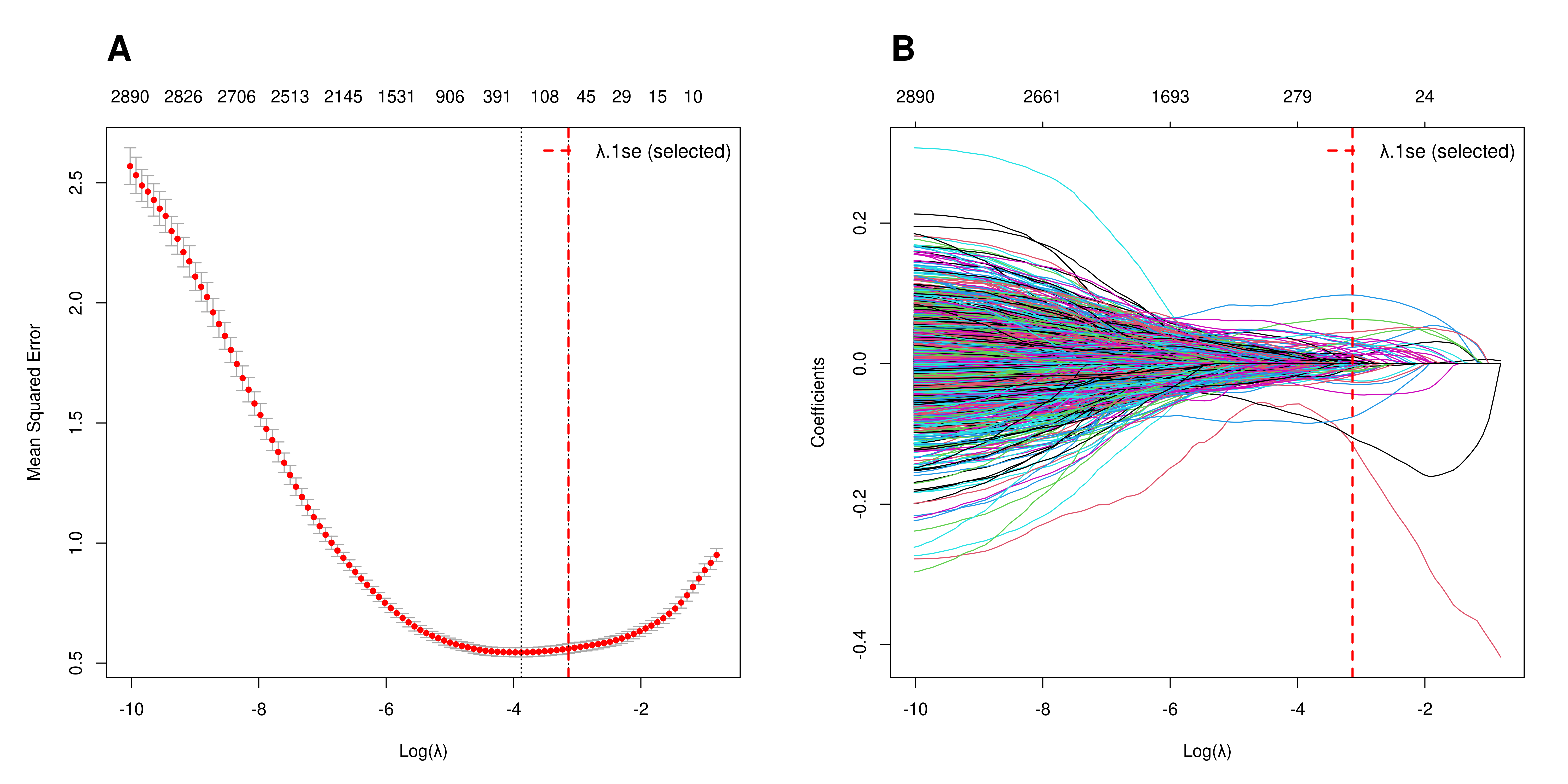
Supplementary Figure 2. LASSO regression analysis for protein biomarker selection in liver fat content prediction.**

(A) Cross-validation mean squared error (MSE) plot across different penalty parameters (lambda). The dashed red line indicates the selected lambda.1se value.

(B) Coefficient paths showing shrinkage of protein coefficients with increasing lambda penalty. The vertical dashed red line marks the lambda.1se value where the final 10-protein panel was selected.

The analysis included 2,911 proteins with age and sex as unpenalized covariates, using 10-fold cross-validation for parameter optimization.

**
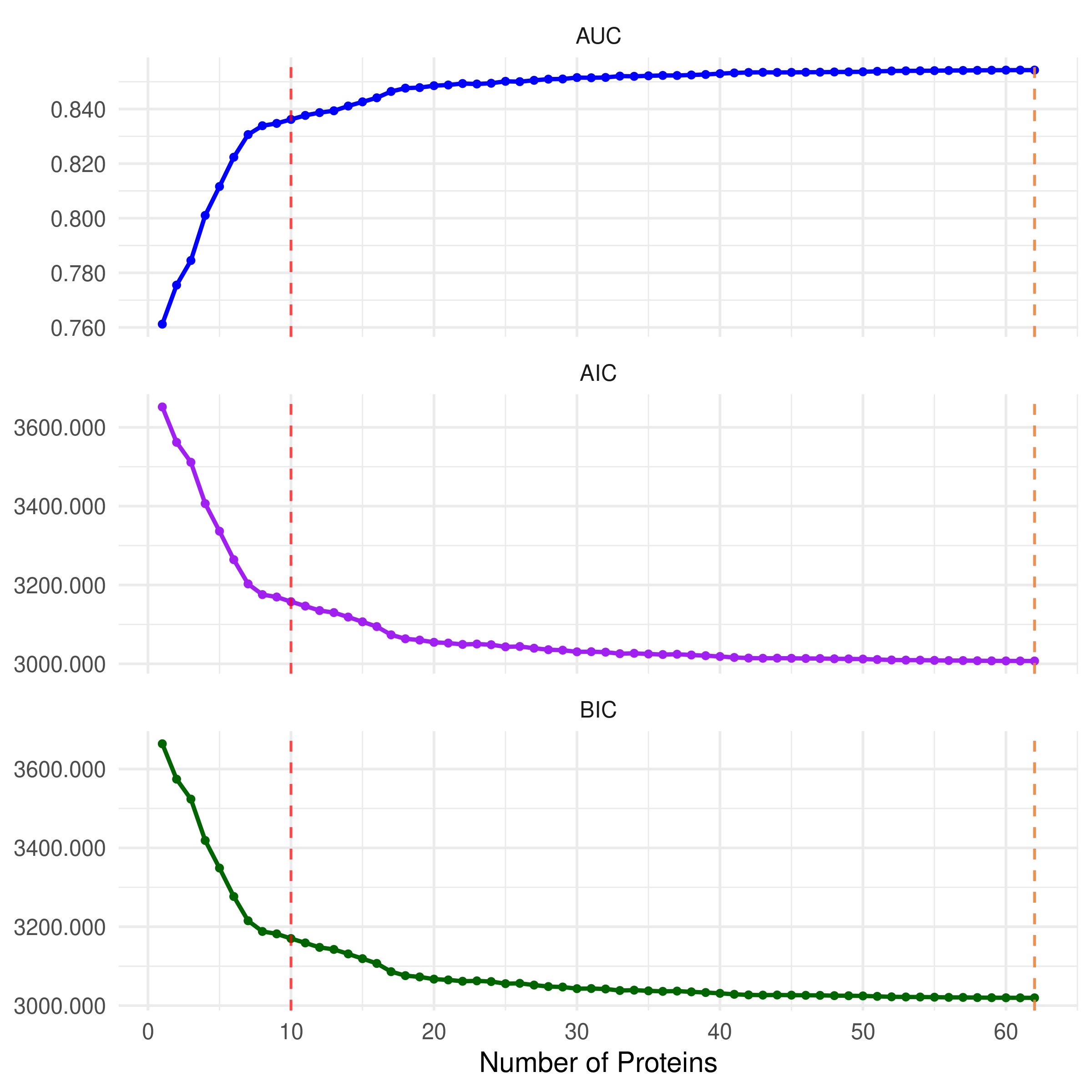
**

**Supplementary Figure 3. Model Selection Analysis for Optimal Protein Number in Liver Fat Content Prediction.**

Performance metrics for models containing 1 to 62 proteins in the derivation set (n=3,726). Top: Area under the receiver operating characteristic curve (AUC) for detecting hepatic steatosis (MRI-PDFF >5%). Middle: Akaike Information Criterion (AIC). Bottom: Bayesian Information Criterion (BIC). The red dashed line indicates the selected 10-protein model (AUC=0.836), which achieved 97.9% of the maximum performance (AUC=0.854 with 62 proteins). The 10-protein model was selected as the optimal balance between predictive accuracy, statistical parsimony, and clinical feasibility.

**
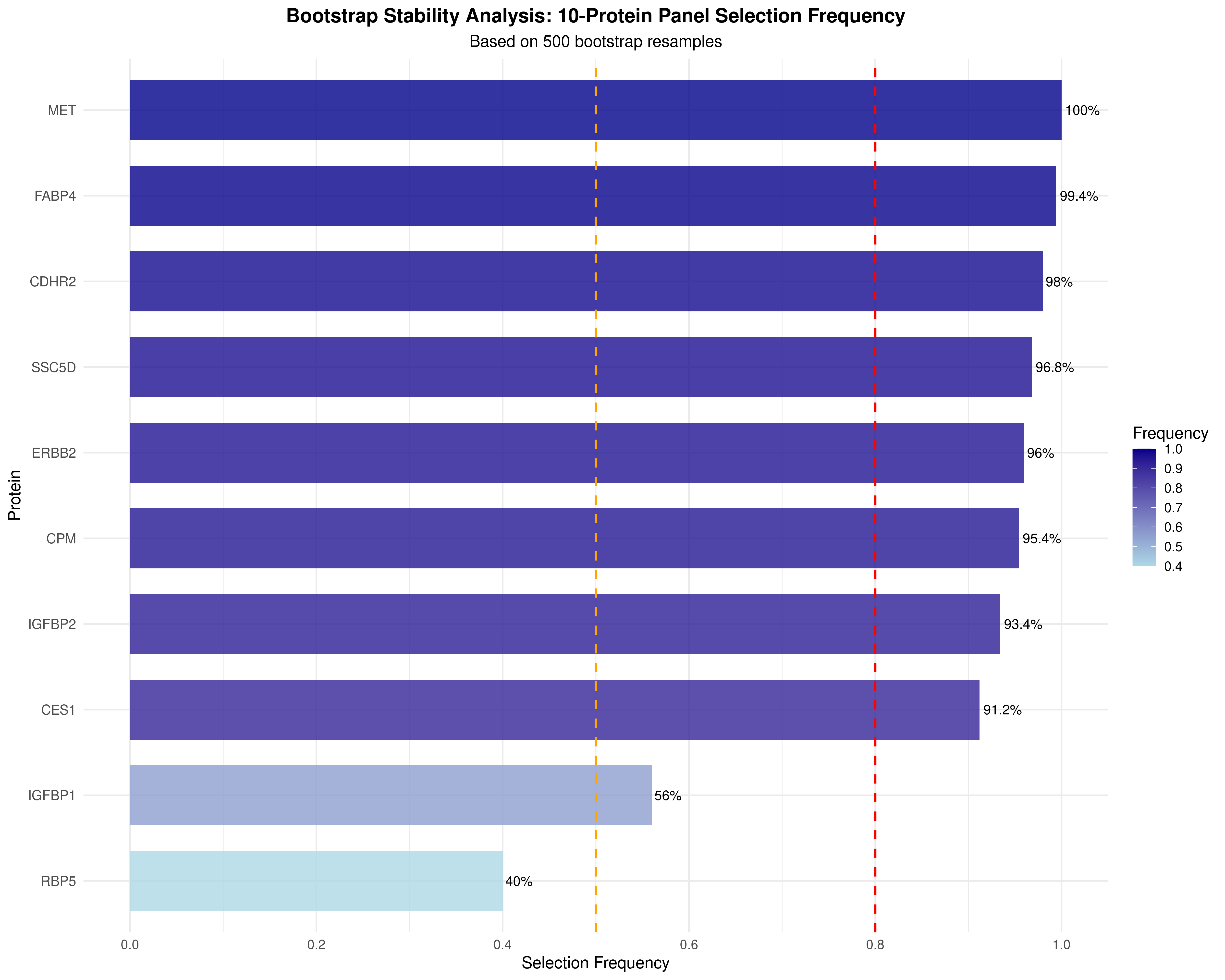
**

**Supplementary Figure 4. Selection frequency of the 10-protein liver fat content panel across 500 bootstrap iterations.**

Dashed red and orange lines indicate 80% and 50% selection thresholds, respectively.


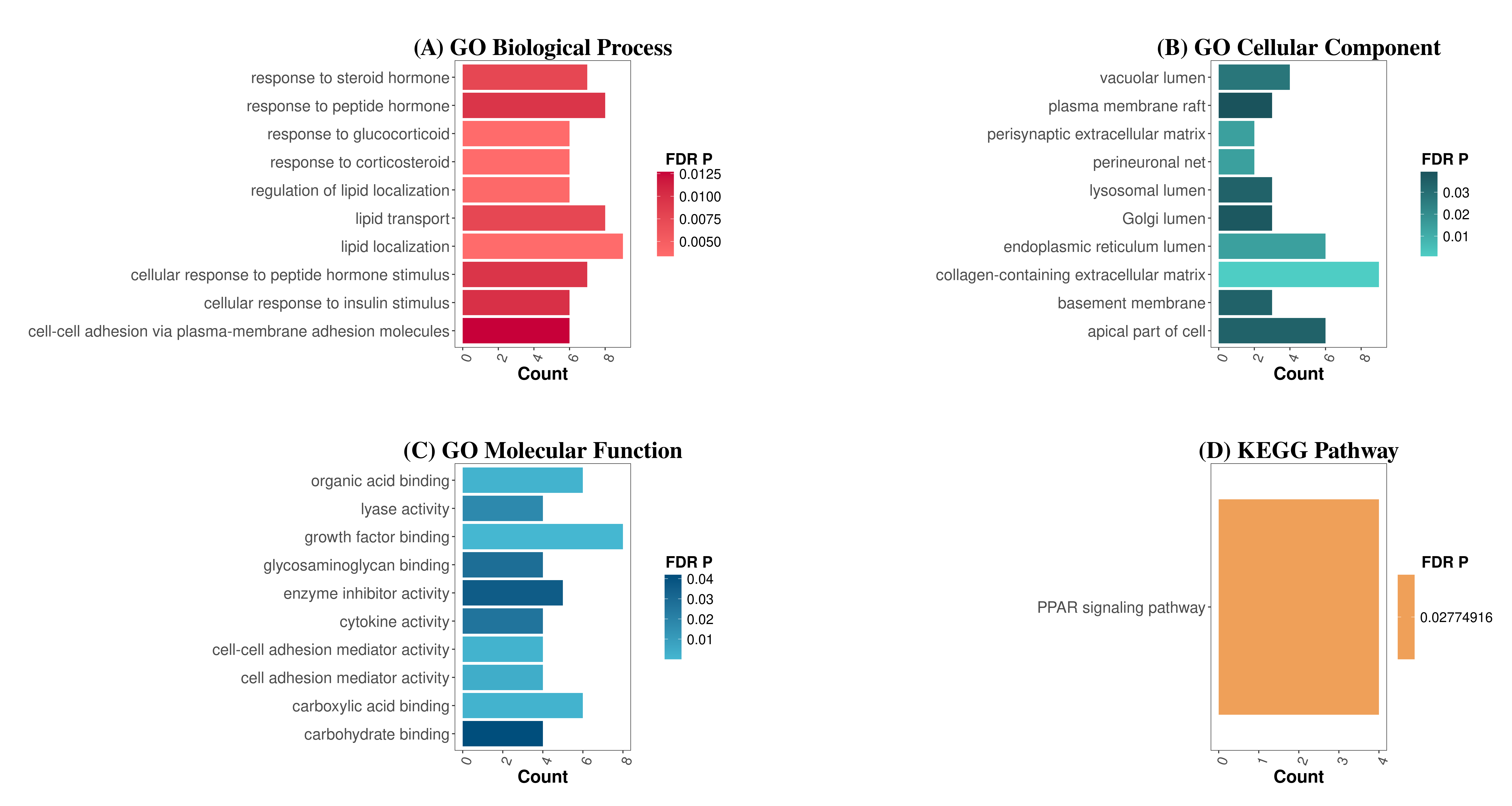


**Supplementary Figure 5. Functional Enrichment Analysis of the 62 LFC-related plasma proteins.**

A. GO Biological Process (Top 10 gene count); B. GO Cellular Component (Top 10 gene count); C. GO Molecular Function (Top 10 gene count); D. KEGG pathway enrichment analyses.

**Abbreviations**: GO, Gene ontology.

**
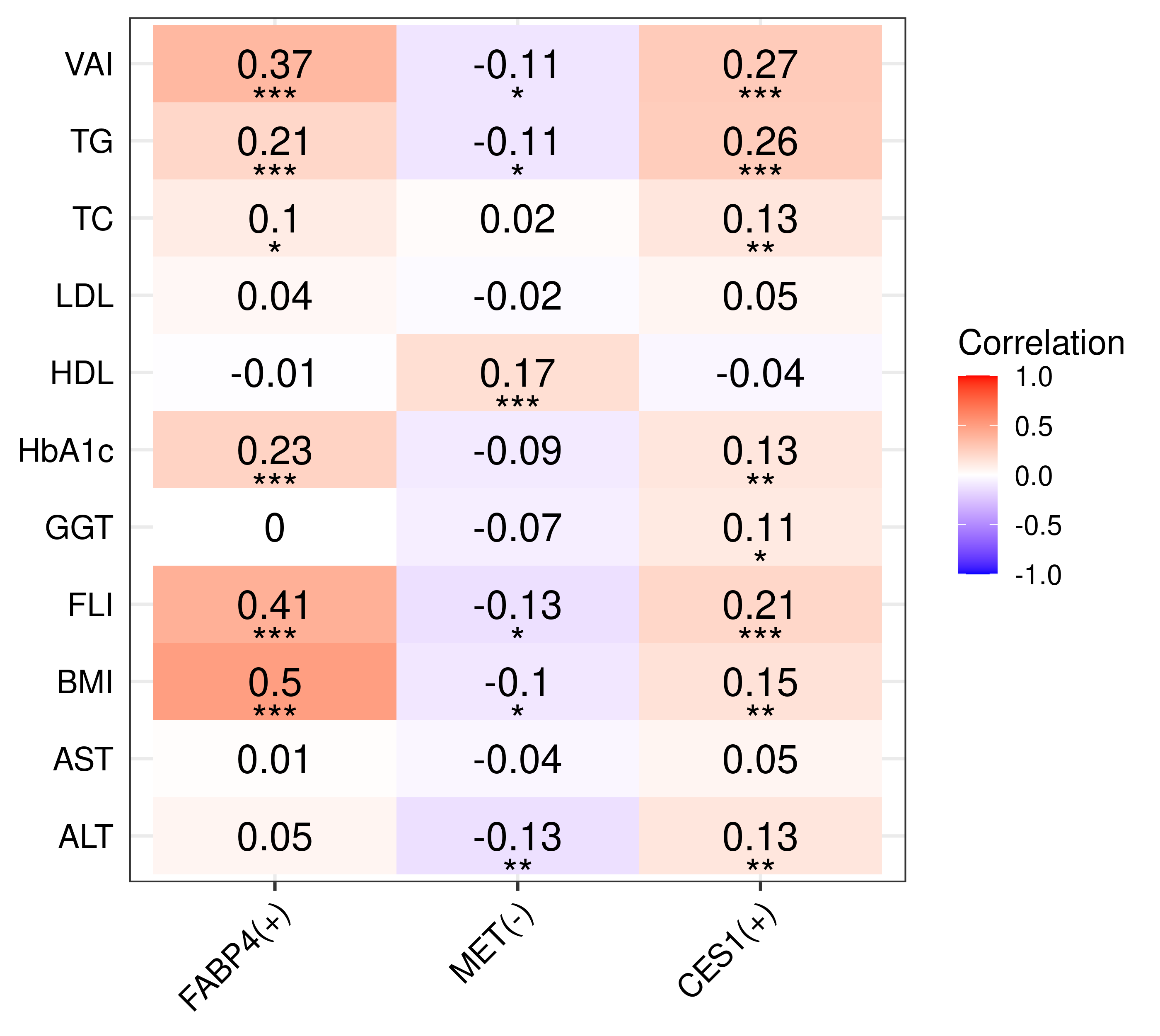
Supplemental Figure 6. Association of FABP4, MET, and CES1 with cardiometabolic risk factors in the Atherosclerosis Risk in Communities (ARIC) study.**

An (+) or (-) marked in proteins indicates the LASSO directionality. *for *P*< 0.05; **for *P*<0.01; ***for *P*<0.001.

**Abbreviations:** TG, triglyceride; TC, total cholesterol; HDL-C, high-density lipoprotein-cholesterol; LDL-C, low-density lipoprotein-cholesterol; HbA1c, glycosylated hemoglobin A1c; BMI, body mass index; VAI, visceral adiposity index; ALT, alanine aminotransferase; AST, aspartate aminotransferase; GGT, gamma-glutamyl transferase; FLI, fatty liver index; LASSO, least absolute shrinkage and selection operator.


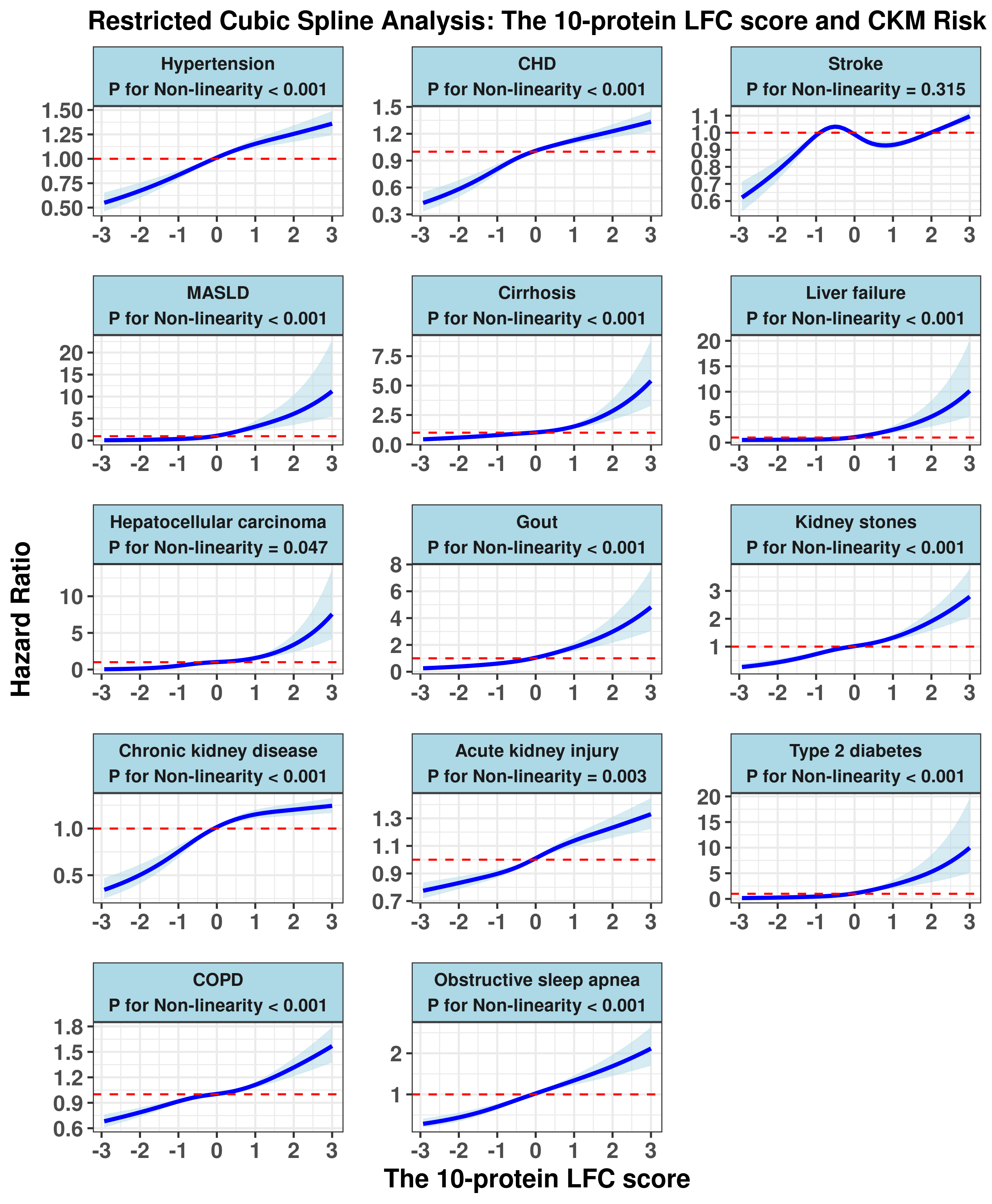


**Supplementary Figure 7. Restricted cubic spline curves for the associations between the simplified 10-protein LFC score and the risk of 14 CKM outcomes.***

*All models were adjusted for age, sex, race, BMI, Townsend deprivation index, smoking, alcohol, systolic blood pressure, LDL, and HbA1c.

**Abbreviations:** LFC, liver fat content; CKM, cardiovascular-kidney-metabolic; COPD, chronic obstructive pulmonary disease; BMI, body mass index; MASLD, metabolic dysfunction-associated steatotic liver disease; CHD, coronary heart disease; HbA1c, glycosylated hemoglobin A1c; LDL-C, low-density lipoprotein-cholesterol.


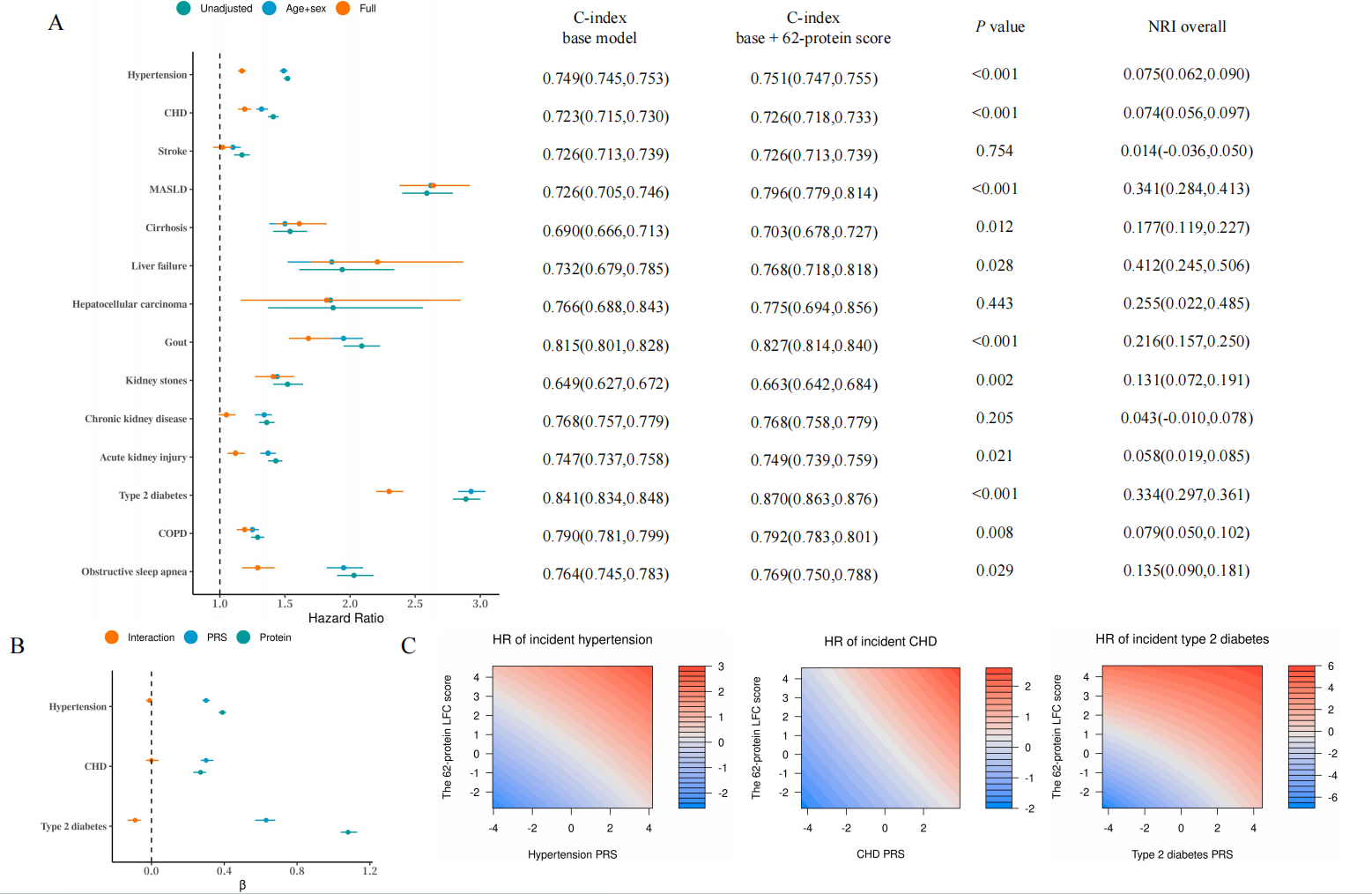


**Supplementary Figure 8. Association of the 62-protein LFC score and polygenic risk with the risk of 14 cardiovascular-kidney-metabolic outcomes.**

**A**, Forest plot of Cox model results with proteomic LFC score as the main predictor. The ‘Full’ adjustment model includes adjustment for age, sex, race, BMI, Townsend deprivation index, smoking, alcohol, systolic blood pressure, LDL, and HbA1c. Error bars, 95% CI. The adjoining table reports the C-index for Cox models without proteomic LFC score (Base) and with the score (Score). Base models include age, sex, race, BMI, Townsend deprivation index, smoking, alcohol, systolic blood pressure, LDL, and HbA1c. Reported *P* value is from comparison testing of C-indices.

**B**, Cox beta coefficients from models including an interaction between the protein score of LFC and PRSs of the indicated conditions or diseases. Error bars, 95% CI.

**C**, Contour map of the model predicted HR across the range of proteomic LFC score and PRSs. The referent hazard was set at the median of the protein score and median of the PRS.

**Abbreviations:** LFC, liver fat content; COPD, chronic obstructive pulmonary disease; BMI, body mass index; MASLD, metabolic dysfunction-associated steatotic liver disease; CHD, coronary heart disease; HbA1c, glycosylated hemoglobin A1c; LDL-C, low-density lipoprotein-cholesterol; PRS, polygenic risk scores.


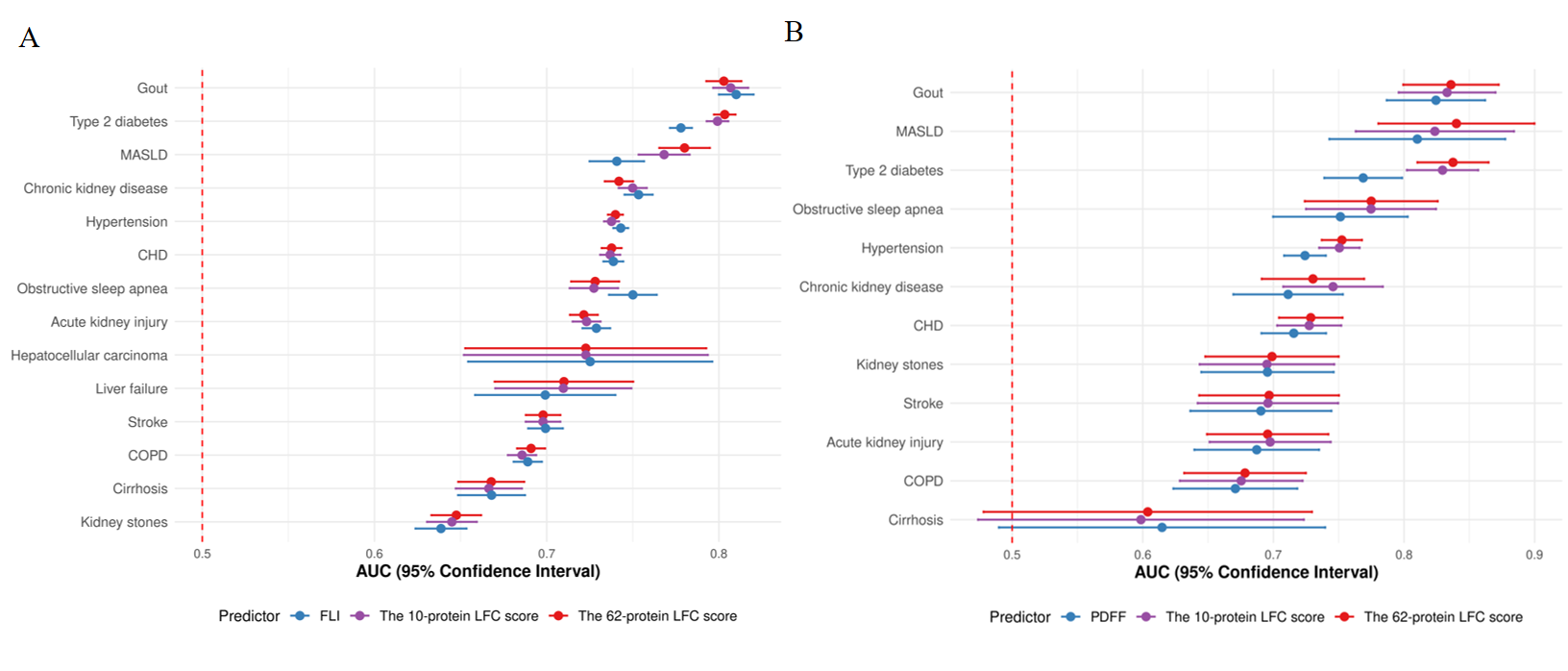


**Supplementary Figure 9. The AUC for predicting cardiovascular-kidney-metabolic outcomes.**

**(A) Forest plot of protein-based scores and FLI for predicting CKM outcomes.** All models were adjusted for age and sex. This analysis was conducted among 53,017 participants with proteomic data. Predictors included: 62-protein liver fat content (LFC) score, 10-protein LFC score, and fatty liver index (FLI).

**(B) Forest plot of protein-based scores and PDFF for predicting CKM outcomes.** All models were adjusted for age and sex. This analysis was conducted among 5,320 participants who had both PDFF measurements and protein scores available. Results for liver failure (n=6) and hepatocellular carcinoma (n=3) are not shown due to small sample sizes. Predictors included: 62-protein liver fat content (LFC) score, 10-protein LFC score, and proton density fat fraction (PDFF).

**Abbreviations:** CHD, coronary heart disease; MASLD, metabolic dysfunction-associated steatotic liver disease; COPD, chronic obstructive pulmonary disease.


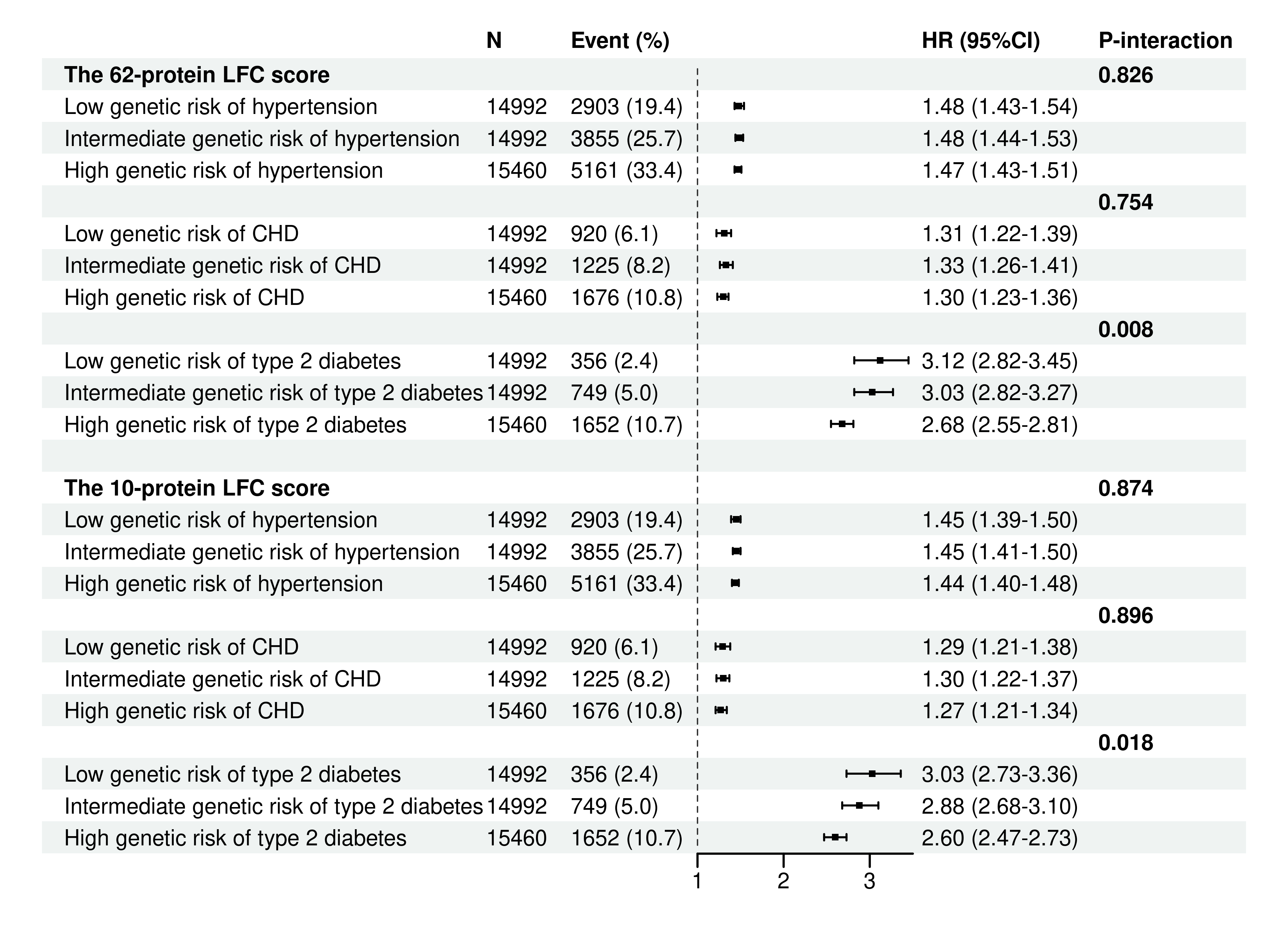


**Supplementary Figure 10. Association between the 62-protein LFC score (A) and 10-protein LFC score (B) with clinical outcomes stratified by genetic risk. ***

*Forest plot displays hazard ratios for the association between the protein scores and the specific disease corresponding to the genetic stratification used, adjusted for age, sex, race, and four principal components of genetic ancestry. For example, in the hypertension genetic risk stratification, all displayed HRs are for hypertension outcomes. Genetic risk was categorized as low, intermediate, or high based on tertiles of polygenic risk scores.

**Abbreviations:** LFC, liver fat content; CHD, coronary heart disease.

**
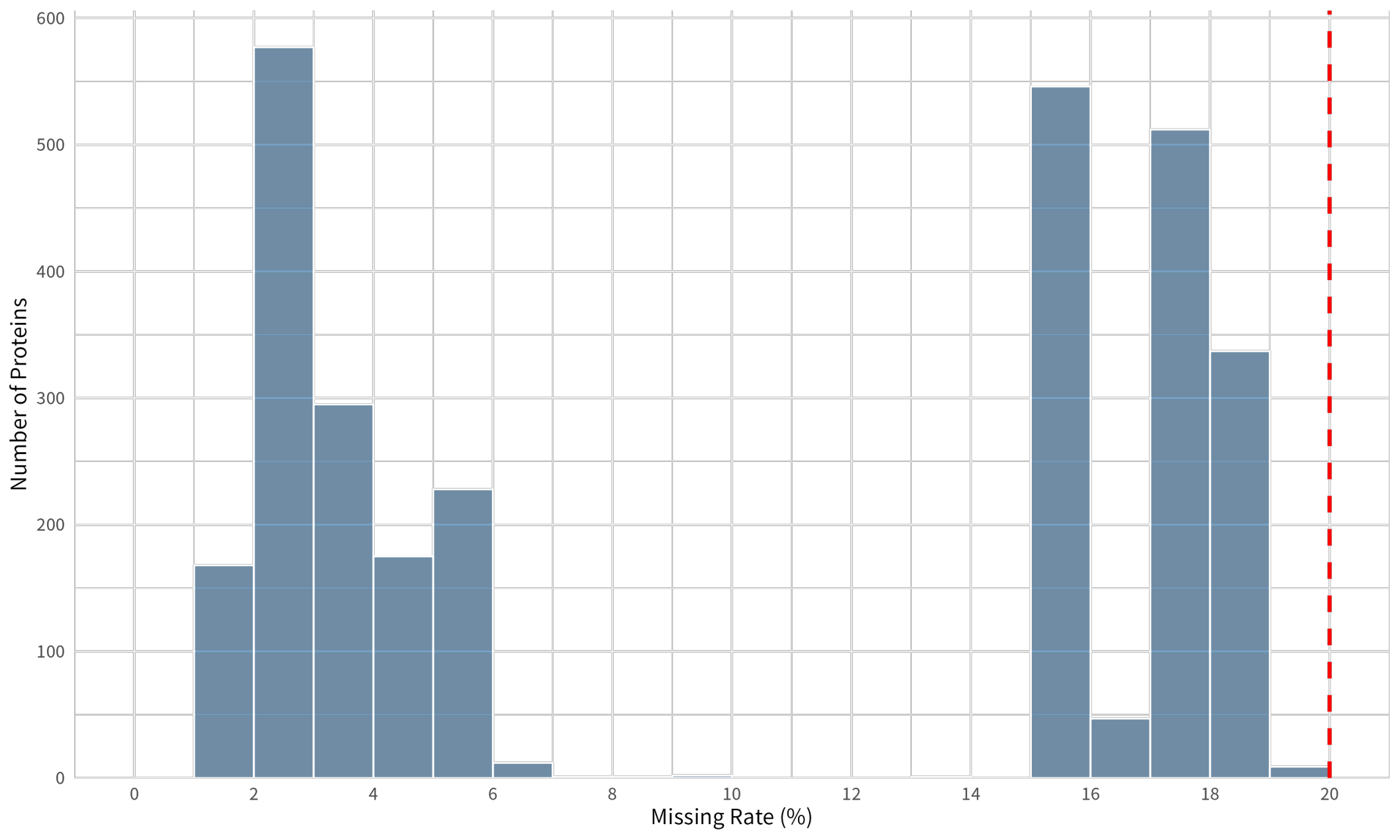
Supplementary Figure 11. Distribution of missing data rates among 2,911 plasma proteins included in analysis**.

The distribution exhibited distinct clustering, with proteins predominantly distributed in low (1-7.5%) and high (13.5-20%) missingness ranges, separated by a region of sparse data (7.5-13.5%). Red dashed line indicates the 20% exclusion threshold.


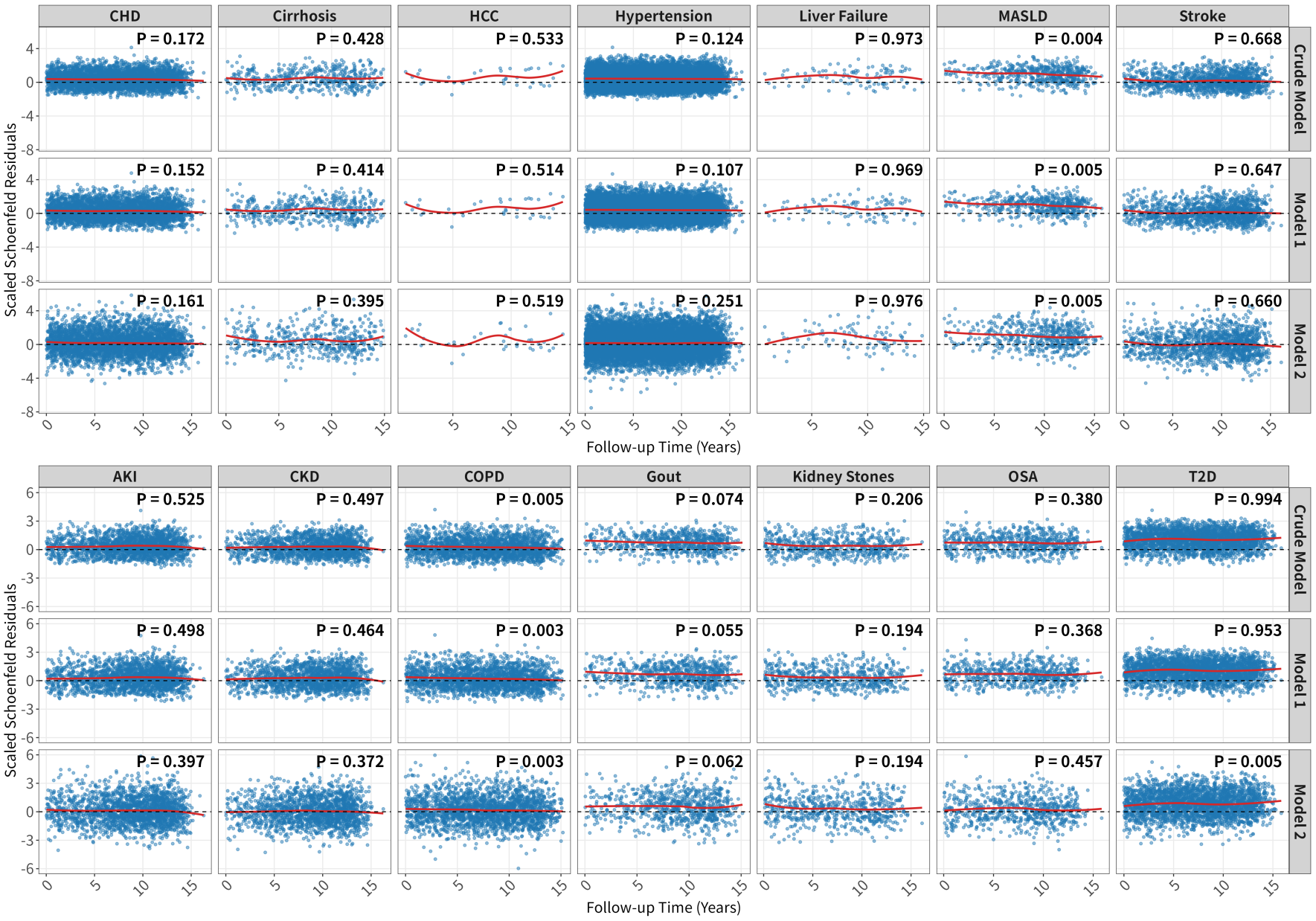


**Supplementary Figure 12. Schoenfeld residual plots for testing proportional hazards in the association between the 62-protein score and CKM diseases. ***

*****Scaled Schoenfeld residuals are plotted against follow-up time for the association between the 62-protein score and CKM diseases across three adjustment levels: (A) Crude model, (B) Model 1 (adjusted for age and sex), and (C) Model 2 (adjusted for age, sex, race, BMI, Townsend deprivation index, smoking status, alcohol use, systolic blood pressure, LDL cholesterol, and HbA1c). The red line represents a loess smooth curve, while the dashed horizontal line at y=0 indicates the expected pattern under proportional hazards. *P*-values test the null hypothesis of proportional hazards. All models showed satisfactory adherence to the proportional hazards assumption after Bonferroni correction (*P*<0.001).

**Abbreviations:** CHD, coronary heart disease; MASLD, metabolic dysfunction-associated steatotic liver disease; HCC, hepatocellular carcinoma; CKD, chronic kidney disease; AKI, acute kidney injury; T2D, type 2 diabetes; COPD, chronic obstructive pulmonary disease; OSA, obstructive sleep apnea; BMI, body mass index; HbA1c, glycated hemoglobin; LDL, low-density lipoprotein.


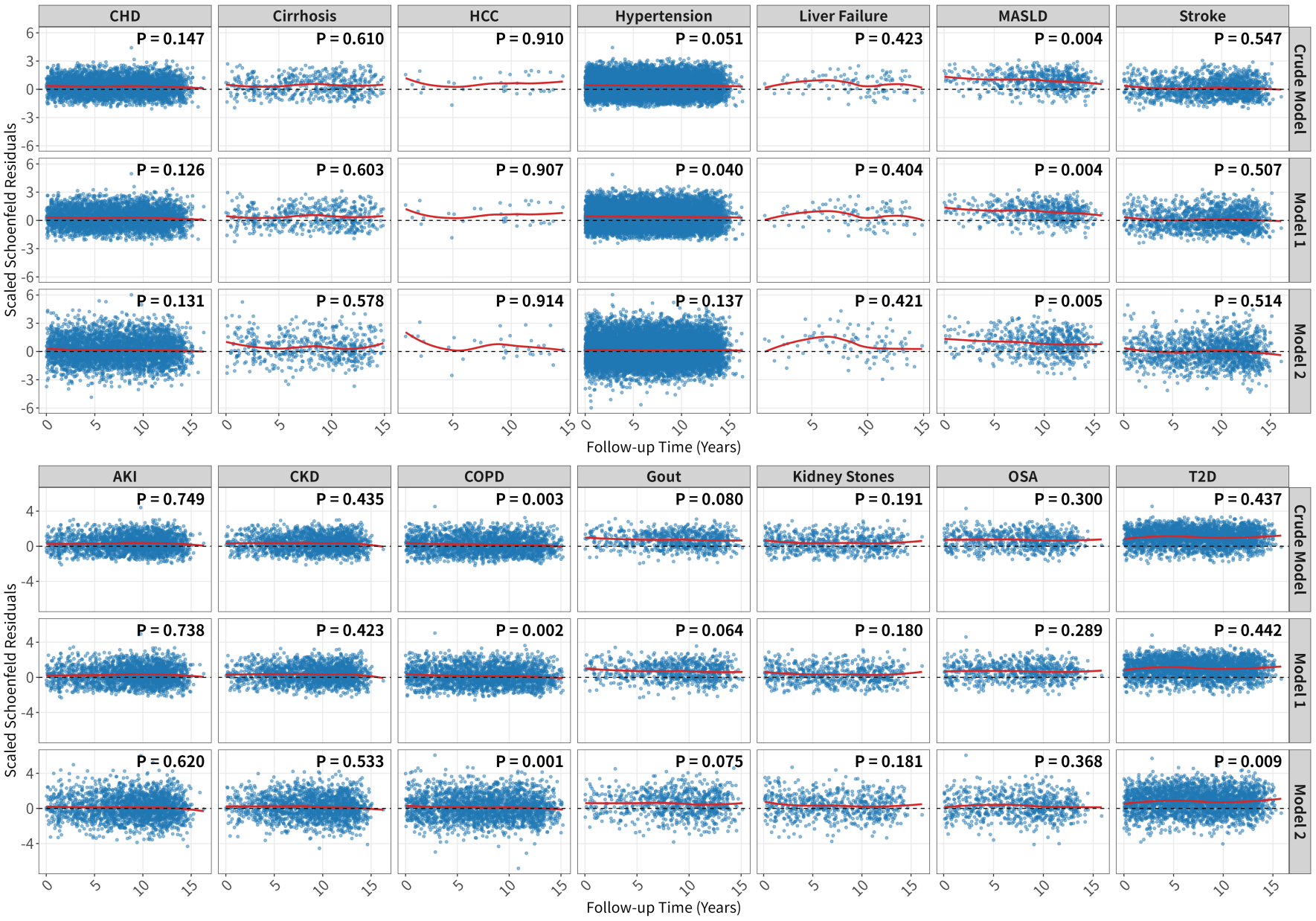


**Supplementary Figure 13. Schoenfeld residual plots for testing proportional hazards in the association between the 10-protein score and CKM diseases. ***

*****Scaled Schoenfeld residuals are plotted against follow-up time for the association between the 10-protein score and CKM diseases across three adjustment levels: (A) Crude model, (B) Model 1 (adjusted for age and sex), and (C) Model 2 (adjusted for age, sex, race, BMI, Townsend deprivation index, smoking status, alcohol use, systolic blood pressure, LDL cholesterol, and HbA1c). The red line represents a loess smooth curve, while the dashed horizontal line at y=0 indicates the expected pattern under proportional hazards. *P*-values test the null hypothesis of proportional hazards. All models showed satisfactory adherence to the proportional hazards assumption after Bonferroni correction (*P*<0.001).

**Abbreviations:** CHD, coronary heart disease; MASLD, metabolic dysfunction-associated steatotic liver disease; HCC, hepatocellular carcinoma; CKD, chronic kidney disease; AKI, acute kidney injury; T2D, type 2 diabetes; COPD, chronic obstructive pulmonary disease; OSA, obstructive sleep apnea; BMI, body mass index; HbA1c, glycated hemoglobin; LDL, low-density lipoprotein.

**Supplementary Table 1. Protein Weights and Model Performance for Proteomic LFC Score. ***

| **Protein** | **Protein name** | **UniProt** | **Panel** | **Weight** | **AUC** | **AIC** | **BIC** |
| --- | --- | --- | --- | --- | --- | --- | --- |
| IGFBP2 | Insulin-like growth factor-binding protein 2 | P18065 | Cardiometabolic | -0.105323598 | 0.761 | 3652 | 3664 |
| FABP4 | Fatty acid-binding protein, adipocyte | P15090 | Cardiometabolic | 0.097725286 | 0.775 | 3562 | 3574 |
| MET | Hepatocyte growth factor receptor | P08581 | Cardiometabolic | -0.075850471 | 0.785 | 3511 | 3524 |
| CPM | Carboxypeptidase M | P14384 | Neurology | 0.06301553 | 0.801 | 3406 | 3419 |
| CES1 | Liver carboxylesterase 1 | P23141 | Cardiometabolic | 0.04489469 | 0.812 | 3336 | 3349 |
| IGFBP1 | Insulin-like growth factor-binding protein 1 | P08833 | Cardiometabolic | -0.043484938 | 0.822 | 3264 | 3277 |
| CDHR2 | Cadherin-related family member 2 | Q9BYE9 | Oncology | 0.036340977 | 0.831 | 3203 | 3215 |
| RBP5 | Retinol-binding protein 5 | P82980 | Oncology | 0.034115469 | 0.834 | 3176 | 3188 |
| ERBB2 | Receptor tyrosine-protein kinase erbB-2 | P04626 | Oncology | 0.033889746 | 0.835 | 3170 | 3182 |
| SSC5D | Soluble scavenger receptor cysteine-rich domain-containing protein SSC5D | A1L4H1 | Cardiometabolic | 0.032098558 | 0.836 | 3158 | 3170 |
| DSG2 | Desmoglein-2 | Q14126 | Neurology | -0.02941007 | 0.838 | 3147 | 3159 |
| PTS | 6-pyruvoyl tetrahydrobiopterin synthase | Q03393 | Neurology | 0.028572764 | 0.839 | 3135 | 3148 |
| AFM | Afamin | P43652 | Inflammation II | 0.028017665 | 0.839 | 3130 | 3142 |
| BPIFB2 | BPI fold-containing family B member 2 | Q8N4F0 | Cardiometabolic II | 0.027998592 | 0.841 | 3119 | 3131 |
| GUSB | Beta-glucuronidase | P08236 | Cardiometabolic | 0.027961152 | 0.843 | 3107 | 3119 |
| SELE | E-selectin | P16581 | Cardiometabolic | 0.027471172 | 0.844 | 3094 | 3107 |
| FGF21 | Fibroblast growth factor 21 | Q9NSA1 | Oncology | 0.027368921 | 0.846 | 3074 | 3086 |
| WFIKKN2 | WAP, Kazal, immunoglobulin, Kunitz and NTR domain-containing protein 2 | Q8TEU8 | Inflammation | -0.02644152 | 0.848 | 3063 | 3076 |
| ACY1 | Aminoacylase-1 | Q03154 | Cardiometabolic | 0.025963847 | 0.848 | 3060 | 3073 |
| CA14 | Carbonic anhydrase 14 | Q9ULX7 | Oncology | -0.023512862 | 0.849 | 3055 | 3067 |
| IGSF9 | Protein turtle homolog A | Q9P2J2 | Cardiometabolic II | 0.023153273 | 0.849 | 3053 | 3065 |
| CTSO | Cathepsin O | P43234 | Inflammation | 0.018425566 | 0.849 | 3049 | 3062 |
| SSC4D | Scavenger receptor cysteine-rich domain-containing group B protein | Q8WTU2 | Cardiometabolic | 0.017593856 | 0.849 | 3050 | 3063 |
| COL4A1 | Collagen alpha-1(IV) chain | P02462 | Cardiometabolic | -0.017450843 | 0.849 | 3048 | 3061 |
| CA9 | Carbonic anhydrase 9 | Q16790 | Oncology | -0.016685756 | 0.85 | 3043 | 3056 |
| IL1RN | Interleukin-1 receptor antagonist protein | P18510 | Inflammation | 0.01451493 | 0.85 | 3044 | 3056 |
| DPP10 | Inactive dipeptidyl peptidase 10 | Q8N608 | Inflammation | -0.014510244 | 0.851 | 3040 | 3052 |
| ADIPOQ | Adiponectin | Q15848 | Inflammation II | -0.013713855 | 0.851 | 3036 | 3048 |
| ACP5 | Tartrate-resistant acid phosphatase type 5 | P13686 | Cardiometabolic | 0.013533804 | 0.851 | 3035 | 3047 |
| CD8A | T-cell surface glycoprotein CD8 alpha chain | P01732 | Neurology | 0.012858813 | 0.852 | 3030 | 3043 |
| PLAT | Tissue-type plasminogen activator | P00750 | Cardiometabolic | 0.012111204 | 0.851 | 3031 | 3043 |
| SELPLG | P-selectin glycoprotein ligand 1 | Q14242 | Inflammation | 0.01176547 | 0.852 | 3030 | 3042 |
| LPL | Lipoprotein lipase | P06858 | Cardiometabolic | -0.011455278 | 0.852 | 3026 | 3038 |
| INHBC | Inhibin beta C chain | P55103 | Neurology | 0.010805107 | 0.852 | 3027 | 3039 |
| CREG1 | Protein CREG1 | O75629 | Oncology | 0.01039558 | 0.852 | 3025 | 3037 |
| NTRK3 | NT-3 growth factor receptor | Q16288 | Neurology | -0.009679028 | 0.852 | 3024 | 3036 |
| FURIN | Furin | P09958 | Oncology | 0.00926312 | 0.852 | 3024 | 3037 |
| PLA2G1B | Phospholipase A2 | P04054 | Cardiometabolic | -0.008362678 | 0.852 | 3022 | 3035 |
| GUCA2A | Guanylin | Q02747 | Neurology | -0.006618192 | 0.853 | 3021 | 3033 |
| BPIFB1 | BPI fold-containing family B member 1 | Q8TDL5 | Cardiometabolic | -0.005718072 | 0.853 | 3019 | 3031 |
| SDHB | Succinate dehydrogenase | P21912 | Oncology II | -0.00554988 | 0.853 | 3016 | 3029 |
| GPRC5C | G-protein coupled receptor family C group 5 member C | Q9NQ84 | Oncology II | -0.004684098 | 0.853 | 3015 | 3027 |
| MENT | Protein MENT | Q9BUN1 | Inflammation II | -0.004677534 | 0.853 | 3014 | 3027 |
| PAMR1 | Inactive serine protease PAMR1 | Q6UXH9 | Neurology | 0.004664498 | 0.853 | 3014 | 3027 |
| KRT18 | Keratin, type I cytoskeletal 18 | P05783 | Oncology | 0.004459399 | 0.853 | 3014 | 3026 |
| FRZB | Secreted frizzled-related protein 3 | Q92765 | Neurology | 0.004137944 | 0.853 | 3014 | 3026 |
| SERPINF1 | Pigment epithelium-derived factor | P36955 | Inflammation II | 0.004137159 | 0.853 | 3013 | 3026 |
| BCAN | Brevican core protein | Q96GW7 | Neurology | -0.003803997 | 0.854 | 3013 | 3025 |
| PRAP1 | Proline-rich acidic protein 1 | Q96NZ9 | Cardiometabolic II | 0.003563269 | 0.854 | 3013 | 3025 |
| MATN3 | Matrilin-3 | O15232 | Neurology | -0.003499057 | 0.854 | 3012 | 3025 |
| NEFL | Neurofilament light polypeptide | P07196 | Neurology | -0.0032488 | 0.854 | 3011 | 3023 |
| ENTPD2 | Ectonucleoside triphosphate diphosphohydrolase 2 | Q9Y5L3 | Oncology | -0.002943213 | 0.854 | 3010 | 3022 |
| SFRP4 | Secreted frizzled-related protein 4 | Q6FHJ7 | Inflammation II | 0.002867946 | 0.854 | 3009 | 3022 |
| CBLN4 | Cerebellin-4 | Q9NTU7 | Oncology | 0.002556213 | 0.854 | 3009 | 3022 |
| CNTN5 | Contactin-5 | O94779 | Neurology | -0.00212208 | 0.854 | 3009 | 3021 |
| SCGB3A2 | Secretoglobin family 3A member 2 | Q96PL1 | Inflammation | -0.001557042 | 0.854 | 3008 | 3021 |
| ITGBL1 | Integrin beta-like protein 1 | O95965 | Cardiometabolic II | 0.001525225 | 0.854 | 3008 | 3021 |
| SLITRK6 | SLIT and NTRK-like protein 6 | Q9H5Y7 | Cardiometabolic | -0.001450863 | 0.854 | 3008 | 3020 |
| IL32 | Interleukin-32 | P24001 | Inflammation | -0.000924893 | 0.854 | 3008 | 3020 |
| APOA2 | Apolipoprotein A-II | P02652 | Inflammation II | -0.000829448 | 0.854 | 3007 | 3020 |
| NCAN | Neurocan core protein | O14594 | Neurology | -0.000725346 | 0.854 | 3007 | 3020 |
| DDC | Aromatic-L-amino-acid decarboxylase | P20711 | Cardiometabolic | -0.000106535 | 0.854 | 3007 | 3020 |

*Proteins are sorted by the absolute value of their LASSO coefficients in descending order. AUC, area under the receiver operating characteristic curve for predicting hepatic steatosis (MRI-PDFF >5%). Performance metrics represent the cumulative model including all proteins up to and including the current row.

**Abbreviations**: LFC, liver fat content; AIC, Akaike Information Criterion; BIC, Bayesian Information Criterion.

**Supplementary Table 2. Comprehensive model performance for predicting hepatic steatosis (MRI-PDFF >5%) and continuous liver fat content (MRI-PDFF) in derivation and validation set.**

| **Model** | **AUC (95% CI)** | **Calibration Intercept (95% CI)** | **Calibration slope (95% CI)** | **Brier Score (95% CI)** | **R^2^** | **RMSE (%)** | **MAE (%)** |
| --- | --- | --- | --- | --- | --- | --- | --- |
| **Derivation set** | | | | | | | |
| The 62-protein LFC score | 0.854(0.841,0.867) | 0.002(-0.107,0.119) | 1.001(0.932,1.071) | 0.130(0.124,0.136) | 0.32 | 3.99 | 2.48 |
| The 10-protein LFC score | 0.836(0.822,0.850) | 0.002(-0.107,0.118) | 1.002(0.931,1.074) | 0.137(0.131,0.144) | 0.28 | 4.09 | 2.55 |
| FLI | 0.781(0.765,0.798) | 0.001(-0.106,0.115) | 1.001(0.920,1.083) | 0.156(0.149,0.163) | 0.18 | 4.36 | 2.66 |
| **Validation set** | | | | | | | |
| The 62-protein LFC score | 0.868(0.849,0.887) | 0.001(-0.161,0.173) | 1.004(0.905,1.111) | 0.125(0.116,0.135) | 0.34 | 3.96 | 2.50 |
| The 10-protein LFC score | 0.850(0.829,0.870) | 0.001(-0.158,0.161) | 1.003(0.900,1.110) | 0.131(0.122,0.141) | 0.30 | 4.07 | 2.56 |
| FLI | 0.794(0.771,0.818) | -0.000(-0.150,0.151) | 1.002(0.899,1.115) | 0.153(0.144,0.162) | 0.22 | 4.32 | 2.65 |

**Notes:** AUC measures discriminatory ability for classifying hepatic steatosis (MRI-PDFF >5%). Calibration intercept and slope assess the agreement between predicted probabilities and observed outcomes; ideal values are 0 and 1, respectively. Brier score measures overall model performance for binary outcomes; ranges from 0 to 1, with lower values indicating better accuracy. R² represents the proportion of variance in continuous MRI-PDFF explained by the model. RMSE and MAE measure prediction accuracy for continuous MRI-PDFF values, with lower values indicating better performance.

**Abbreviations:** AUC, Area under the receiver operating characteristic curve; LFC, liver fat content; MRI, magnetic resonance imaging; PDFF, proton density fat fraction; RMSE, root mean square error; MAE, mean absolute error.

**Supplementary Table 3. Significant enrichment of the 62 LFC-related plasma proteins. ***

| **Enrichment analyses** | **ID** | **Term** | **GeneID** | **Observed gene count** | ***P*** value | **FDR-corrected** P value |
| --- | --- | --- | --- | --- | --- | --- |
| Biological Process (Gene Ontology) | GO:0010876 | lipid localization | FABP4/CES1/RBP5/ADIPOQ/LPL/FURIN/PLA2G1B/PRAP1/APOA2 | 9 | 1.33641892839731e-05 | 0.00337441782515804 |
| Biological Process (Gene Ontology) | GO:0006869 | lipid transport | FABP4/CES1/RBP5/ADIPOQ/FURIN/PLA2G1B/PRAP1/APOA2 | 8 | 6.96574092374574e-05 | 0.00761944891812804 |
| Biological Process (Gene Ontology) | GO:0043434 | response to peptide hormone | MET/IGFBP1/ERBB2/FGF21/ADIPOQ/NTRK3/PLA2G1B/NEFL | 8 | 0.000114303573115335 | 0.00950440858438682 |
| Biological Process (Gene Ontology) | GO:0048545 | response to steroid hormone | IGFBP2/DSG2/IL1RN/ADIPOQ/SERPINF1/NEFL/APOA2 | 7 | 6.9610772551832e-05 | 0.00761944891812804 |
| Biological Process (Gene Ontology) | GO:0071375 | cellular response to peptide hormone stimulus | MET/IGFBP1/ERBB2/FGF21/ADIPOQ/NTRK3/PLA2G1B | 7 | 0.000120308969422618 | 0.00950440858438682 |
| Biological Process (Gene Ontology) | GO:0051384 | response to glucocorticoid | IGFBP2/IL1RN/ADIPOQ/SERPINF1/NEFL/APOA2 | 6 | 3.43349482599966e-06 | 0.00337441782515804 |
| Biological Process (Gene Ontology) | GO:0031960 | response to corticosteroid | IGFBP2/IL1RN/ADIPOQ/SERPINF1/NEFL/APOA2 | 6 | 7.85694255987249e-06 | 0.00337441782515804 |
| Biological Process (Gene Ontology) | GO:1905952 | regulation of lipid localization | CES1/ADIPOQ/LPL/FURIN/PRAP1/APOA2 | 6 | 2.25671068441558e-05 | 0.00364252826860991 |
| Biological Process (Gene Ontology) | GO:0032869 | cellular response to insulin stimulus | MET/IGFBP1/ERBB2/ADIPOQ/NTRK3/PLA2G1B | 6 | 0.000132561138159805 | 0.00992115465596018 |
| Biological Process (Gene Ontology) | GO:0098742 | cell-cell adhesion via plasma-membrane adhesion molecules | CDHR2/DSG2/SELE/IGSF9/IL1RN/ADIPOQ | 6 | 0.000198220973977455 | 0.0127022300758973 |
| Biological Process (Gene Ontology) | GO:0032868 | response to insulin | MET/IGFBP1/ERBB2/ADIPOQ/NTRK3/PLA2G1B | 6 | 0.000432782458662524 | 0.0157464386210173 |
| Biological Process (Gene Ontology) | GO:0051336 | regulation of hydrolase activity | MET/NTRK3/FURIN/PLA2G1B/SERPINF1/APOA2 | 6 | 0.00050815870411453 | 0.0157464386210173 |
| Biological Process (Gene Ontology) | GO:0016042 | lipid catabolic process | CES1/FGF21/ADIPOQ/LPL/PLA2G1B/APOA2 | 6 | 0.000649669254257311 | 0.0164969585634624 |
| Biological Process (Gene Ontology) | GO:0050727 | regulation of inflammatory response | FABP4/SELE/ADIPOQ/ACP5/LPL/FURIN | 6 | 0.00214786349826391 | 0.0348178155799652 |
| Biological Process (Gene Ontology) | GO:0097006 | regulation of plasma lipoprotein particle levels | FGF21/ADIPOQ/LPL/FURIN/APOA2 | 5 | 1.00654224344174e-05 | 0.00337441782515804 |
| Biological Process (Gene Ontology) | GO:0048009 | insulin-like growth factor receptor signaling pathway | IGFBP2/MET/IGFBP1/ERBB2/NTRK3 | 5 | 1.42380498951816e-05 | 0.00337441782515804 |
| Biological Process (Gene Ontology) | GO:0048008 | platelet-derived growth factor receptor signaling pathway | MET/ERBB2/ADIPOQ/PLAT/NTRK3 | 5 | 1.71646023086428e-05 | 0.00348686635469858 |
| Biological Process (Gene Ontology) | GO:0032368 | regulation of lipid transport | CES1/ADIPOQ/FURIN/PRAP1/APOA2 | 5 | 0.000112272588534029 | 0.00950440858438682 |
| Biological Process (Gene Ontology) | GO:0009749 | response to glucose | FGF21/ADIPOQ/LPL/SERPINF1/APOA2 | 5 | 0.000414094161920282 | 0.0157464386210173 |
| Biological Process (Gene Ontology) | GO:0009746 | response to hexose | FGF21/ADIPOQ/LPL/SERPINF1/APOA2 | 5 | 0.000461521952170186 | 0.0157464386210173 |
| Biological Process (Gene Ontology) | GO:0034284 | response to monosaccharide | FGF21/ADIPOQ/LPL/SERPINF1/APOA2 | 5 | 0.000534672009750059 | 0.0157464386210173 |
| Biological Process (Gene Ontology) | GO:0009743 | response to carbohydrate | FGF21/ADIPOQ/LPL/SERPINF1/APOA2 | 5 | 0.000868831102663113 | 0.0187193610301053 |
| Biological Process (Gene Ontology) | GO:0051347 | positive regulation of transferase activity | ERBB2/ADIPOQ/NTRK3/PLA2G1B/APOA2 | 5 | 0.00119175888975377 | 0.0230687423819104 |
| Biological Process (Gene Ontology) | GO:0071402 | cellular response to lipoprotein particle stimulus | CES1/FGF21/LPL/APOA2 | 4 | 1.03009071394509e-05 | 0.00337441782515804 |
| Biological Process (Gene Ontology) | GO:0035790 | platelet-derived growth factor receptor-alpha signaling pathway | MET/ERBB2/ADIPOQ/NTRK3 | 4 | 2.3053976383607e-05 | 0.00364252826860991 |
| Biological Process (Gene Ontology) | GO:0038063 | collagen-activated tyrosine kinase receptor signaling pathway | MET/ERBB2/COL4A1/NTRK3 | 4 | 2.84859071122212e-05 | 0.00405069599135786 |
| Biological Process (Gene Ontology) | GO:0038065 | collagen-activated signaling pathway | MET/ERBB2/COL4A1/NTRK3 | 4 | 3.71184998512131e-05 | 0.00479840970803864 |
| Biological Process (Gene Ontology) | GO:0032371 | regulation of sterol transport | CES1/ADIPOQ/FURIN/APOA2 | 4 | 8.70572071289827e-05 | 0.00825302323582756 |
| Biological Process (Gene Ontology) | GO:0032374 | regulation of cholesterol transport | CES1/ADIPOQ/FURIN/APOA2 | 4 | 8.70572071289827e-05 | 0.00825302323582756 |
| Biological Process (Gene Ontology) | GO:0032637 | interleukin-8 production | SSC5D/ADIPOQ/PLA2G1B/APOA2 | 4 | 0.000288760557605462 | 0.0136872504304989 |
| Biological Process (Gene Ontology) | GO:0032677 | regulation of interleukin-8 production | SSC5D/ADIPOQ/PLA2G1B/APOA2 | 4 | 0.000288760557605462 | 0.0136872504304989 |
| Biological Process (Gene Ontology) | GO:0043405 | regulation of MAP kinase activity | ERBB2/ADIPOQ/NTRK3/PLA2G1B | 4 | 0.000333055809117475 | 0.0152775922762919 |
| Biological Process (Gene Ontology) | GO:0042632 | cholesterol homeostasis | FABP4/CES1/LPL/APOA2 | 4 | 0.000356938181758709 | 0.0157464386210173 |
| Biological Process (Gene Ontology) | GO:0055092 | sterol homeostasis | FABP4/CES1/LPL/APOA2 | 4 | 0.000369328093580919 | 0.0157464386210173 |
| Biological Process (Gene Ontology) | GO:1905954 | positive regulation of lipid localization | CES1/ADIPOQ/LPL/PRAP1 | 4 | 0.000382022916356158 | 0.0157464386210173 |
| Biological Process (Gene Ontology) | GO:0071902 | positive regulation of protein serine/threonine kinase activity | ERBB2/ADIPOQ/NTRK3/PLA2G1B | 4 | 0.00039502698450051 | 0.0157464386210173 |
| Biological Process (Gene Ontology) | GO:0030301 | cholesterol transport | CES1/ADIPOQ/FURIN/APOA2 | 4 | 0.000526699837975887 | 0.0157464386210173 |
| Biological Process (Gene Ontology) | GO:0015918 | sterol transport | CES1/ADIPOQ/FURIN/APOA2 | 4 | 0.000686979300516776 | 0.0168650850538002 |
| Biological Process (Gene Ontology) | GO:0045834 | positive regulation of lipid metabolic process | CES1/FGF21/ADIPOQ/APOA2 | 4 | 0.000706694705070971 | 0.0170325401798461 |
| Biological Process (Gene Ontology) | GO:0008543 | fibroblast growth factor receptor signaling pathway | MET/ERBB2/FGF21/NTRK3 | 4 | 0.000747323154446185 | 0.0175551994313798 |
| Biological Process (Gene Ontology) | GO:0090090 | negative regulation of canonical Wnt signaling pathway | IGFBP2/IGFBP1/FRZB/SFRP4 | 4 | 0.00107928886045504 | 0.021315954993987 |
| Biological Process (Gene Ontology) | GO:0044344 | cellular response to fibroblast growth factor stimulus | MET/ERBB2/FGF21/NTRK3 | 4 | 0.00157214551468142 | 0.0282986192642656 |
| Biological Process (Gene Ontology) | GO:0071774 | response to fibroblast growth factor | MET/ERBB2/FGF21/NTRK3 | 4 | 0.00186942907102136 | 0.0316467635594331 |
| Biological Process (Gene Ontology) | GO:0008286 | insulin receptor signaling pathway | MET/IGFBP1/ERBB2/NTRK3 | 4 | 0.00199031409580725 | 0.0332967840498578 |
| Biological Process (Gene Ontology) | GO:0030178 | negative regulation of Wnt signaling pathway | IGFBP2/IGFBP1/FRZB/SFRP4 | 4 | 0.00220365921392185 | 0.0348178155799652 |
| Biological Process (Gene Ontology) | GO:0015718 | monocarboxylic acid transport | FABP4/CES1/RBP5/PLA2G1B | 4 | 0.00243231705412429 | 0.0364079457996288 |
| Biological Process (Gene Ontology) | GO:0055088 | lipid homeostasis | FABP4/CES1/LPL/APOA2 | 4 | 0.00243231705412429 | 0.0364079457996288 |
| Biological Process (Gene Ontology) | GO:0034368 | protein-lipid complex remodeling | LPL/FURIN/APOA2 | 3 | 0.000190681713949732 | 0.0127022300758973 |
| Biological Process (Gene Ontology) | GO:0034369 | plasma lipoprotein particle remodeling | LPL/FURIN/APOA2 | 3 | 0.000190681713949732 | 0.0127022300758973 |
| Biological Process (Gene Ontology) | GO:0071404 | cellular response to low-density lipoprotein particle stimulus | CES1/FGF21/LPL | 3 | 0.000206581483088851 | 0.0127022300758973 |
| Biological Process (Gene Ontology) | GO:0034367 | protein-containing complex remodeling | LPL/FURIN/APOA2 | 3 | 0.000223316281221823 | 0.0127022300758973 |
| Biological Process (Gene Ontology) | GO:0055094 | response to lipoprotein particle | CES1/FGF21/LPL | 3 | 0.000223316281221823 | 0.0127022300758973 |
| Biological Process (Gene Ontology) | GO:0060074 | synapse maturation | IGSF9/BCAN/NEFL | 3 | 0.000240904904931611 | 0.0131756451851058 |
| Biological Process (Gene Ontology) | GO:0046461 | neutral lipid catabolic process | FGF21/LPL/APOA2 | 3 | 0.000278717897488556 | 0.0136872504304989 |
| Biological Process (Gene Ontology) | GO:0046464 | acylglycerol catabolic process | FGF21/LPL/APOA2 | 3 | 0.000278717897488556 | 0.0136872504304989 |
| Biological Process (Gene Ontology) | GO:0036323 | vascular endothelial growth factor receptor-1 signaling pathway | MET/ERBB2/NTRK3 | 3 | 0.000440635974123147 | 0.0157464386210173 |
| Biological Process (Gene Ontology) | GO:0036215 | response to stem cell factor | MET/ERBB2/NTRK3 | 3 | 0.000525161068128981 | 0.0157464386210173 |
| Biological Process (Gene Ontology) | GO:0036216 | cellular response to stem cell factor stimulus | MET/ERBB2/NTRK3 | 3 | 0.000525161068128981 | 0.0157464386210173 |
| Biological Process (Gene Ontology) | GO:0038109 | Kit signaling pathway | MET/ERBB2/NTRK3 | 3 | 0.000525161068128981 | 0.0157464386210173 |
| Biological Process (Gene Ontology) | GO:0038145 | macrophage colony-stimulating factor signaling pathway | MET/ERBB2/NTRK3 | 3 | 0.000586892578701769 | 0.0157464386210173 |
| Biological Process (Gene Ontology) | GO:0071827 | plasma lipoprotein particle organization | LPL/FURIN/APOA2 | 3 | 0.000586892578701769 | 0.0157464386210173 |
| Biological Process (Gene Ontology) | GO:0031547 | brain-derived neurotrophic factor receptor signaling pathway | MET/ERBB2/NTRK3 | 3 | 0.000619421728835508 | 0.0160148672437108 |
| Biological Process (Gene Ontology) | GO:0034381 | plasma lipoprotein particle clearance | FGF21/ADIPOQ/APOA2 | 3 | 0.000619421728835508 | 0.0160148672437108 |
| Biological Process (Gene Ontology) | GO:0071825 | protein-lipid complex organization | LPL/FURIN/APOA2 | 3 | 0.00068788673215219 | 0.0168650850538002 |
| Biological Process (Gene Ontology) | GO:0036006 | cellular response to macrophage colony-stimulating factor stimulus | MET/ERBB2/NTRK3 | 3 | 0.000799335738837361 | 0.0175551994313798 |
| Biological Process (Gene Ontology) | GO:0046324 | regulation of D-glucose import | FGF21/ADIPOQ/PLA2G1B | 3 | 0.000799335738837361 | 0.0175551994313798 |
| Biological Process (Gene Ontology) | GO:0048012 | hepatocyte growth factor receptor signaling pathway | MET/ERBB2/NTRK3 | 3 | 0.000799335738837361 | 0.0175551994313798 |
| Biological Process (Gene Ontology) | GO:0034113 | heterotypic cell-cell adhesion | DSG2/IL1RN/ADIPOQ | 3 | 0.000921653263455603 | 0.019044619894467 |
| Biological Process (Gene Ontology) | GO:0035791 | platelet-derived growth factor receptor-beta signaling pathway | MET/ERBB2/NTRK3 | 3 | 0.000921653263455603 | 0.019044619894467 |
| Biological Process (Gene Ontology) | GO:0036005 | response to macrophage colony-stimulating factor | MET/ERBB2/NTRK3 | 3 | 0.000964910598784626 | 0.0196014695924534 |
| Biological Process (Gene Ontology) | GO:0032757 | positive regulation of interleukin-8 production | ADIPOQ/PLA2G1B/APOA2 | 3 | 0.00100943467039816 | 0.0202171281874111 |
| Biological Process (Gene Ontology) | GO:0046503 | glycerolipid catabolic process | FGF21/LPL/APOA2 | 3 | 0.00120048307753964 | 0.0230687423819104 |
| Biological Process (Gene Ontology) | GO:0043406 | positive regulation of MAP kinase activity | ERBB2/NTRK3/PLA2G1B | 3 | 0.00130397351580329 | 0.0247233378596304 |
| Biological Process (Gene Ontology) | GO:0033344 | cholesterol efflux | CES1/ADIPOQ/APOA2 | 3 | 0.00146947164019113 | 0.0272240562787535 |
| Biological Process (Gene Ontology) | GO:0010827 | regulation of D-glucose transmembrane transport | FGF21/ADIPOQ/PLA2G1B | 3 | 0.00170984048009912 | 0.0300171995395179 |
| Biological Process (Gene Ontology) | GO:1904019 | epithelial cell apoptotic process | PLA2G1B/KRT18/SFRP4 | 3 | 0.00183869729350608 | 0.0315015367634415 |
| Biological Process (Gene Ontology) | GO:0046323 | D-glucose import | FGF21/ADIPOQ/PLA2G1B | 3 | 0.00204309756579017 | 0.0337823806808561 |
| Biological Process (Gene Ontology) | GO:0048013 | ephrin receptor signaling pathway | MET/ERBB2/NTRK3 | 3 | 0.00218691220920308 | 0.0348178155799652 |
| Biological Process (Gene Ontology) | GO:0032370 | positive regulation of lipid transport | CES1/ADIPOQ/PRAP1 | 3 | 0.00241418497628195 | 0.0364079457996288 |
| Biological Process (Gene Ontology) | GO:0038084 | vascular endothelial growth factor signaling pathway | MET/ERBB2/NTRK3 | 3 | 0.00299992311469522 | 0.0424897270946758 |
| Biological Process (Gene Ontology) | GO:0120162 | positive regulation of cold-induced thermogenesis | FABP4/FGF21/ADIPOQ | 3 | 0.0035659039672151 | 0.0482925280131416 |
| Biological Process (Gene Ontology) | GO:0034115 | negative regulation of heterotypic cell-cell adhesion | IL1RN/ADIPOQ | 2 | 0.000487841526422365 | 0.0157464386210173 |
| Biological Process (Gene Ontology) | GO:0070587 | regulation of cell-cell adhesion involved in gastrulation | IL1RN/ADIPOQ | 2 | 0.000487841526422365 | 0.0157464386210173 |
| Biological Process (Gene Ontology) | GO:0034370 | triglyceride-rich lipoprotein particle remodeling | LPL/APOA2 | 2 | 0.000584270362650276 | 0.0157464386210173 |
| Biological Process (Gene Ontology) | GO:0034372 | very-low-density lipoprotein particle remodeling | LPL/APOA2 | 2 | 0.000584270362650276 | 0.0157464386210173 |
| Biological Process (Gene Ontology) | GO:0070586 | cell-cell adhesion involved in gastrulation | IL1RN/ADIPOQ | 2 | 0.000584270362650276 | 0.0157464386210173 |
| Biological Process (Gene Ontology) | GO:1904729 | regulation of intestinal lipid absorption | PRAP1/APOA2 | 2 | 0.000584270362650276 | 0.0157464386210173 |
| Biological Process (Gene Ontology) | GO:0032536 | regulation of cell projection size | CDHR2/NEFL | 2 | 0.000802452857271228 | 0.0175551994313798 |
| Biological Process (Gene Ontology) | GO:1904478 | regulation of intestinal absorption | PRAP1/APOA2 | 2 | 0.000802452857271228 | 0.0175551994313798 |
| Biological Process (Gene Ontology) | GO:0099550 | trans-synaptic signaling, modulating synaptic transmission | PLAT/CBLN4 | 2 | 0.000924106028634475 | 0.019044619894467 |
| Biological Process (Gene Ontology) | GO:0010885 | regulation of cholesterol storage | CES1/LPL | 2 | 0.00149330266507931 | 0.0272240562787535 |
| Biological Process (Gene Ontology) | GO:1904037 | positive regulation of epithelial cell apoptotic process | PLA2G1B/SFRP4 | 2 | 0.00149330266507931 | 0.0272240562787535 |
| Biological Process (Gene Ontology) | GO:0043691 | reverse cholesterol transport | CES1/APOA2 | 2 | 0.00165600092505668 | 0.0294354164428826 |
| Biological Process (Gene Ontology) | GO:0034375 | high-density lipoprotein particle remodeling | LPL/APOA2 | 2 | 0.00182676082079524 | 0.0315015367634415 |
| Biological Process (Gene Ontology) | GO:0010955 | negative regulation of protein processing | PLAT/FURIN | 2 | 0.00219227121341162 | 0.0348178155799652 |
| Biological Process (Gene Ontology) | GO:0034114 | regulation of heterotypic cell-cell adhesion | IL1RN/ADIPOQ | 2 | 0.00238692508579594 | 0.0364079457996288 |
| Biological Process (Gene Ontology) | GO:0098856 | intestinal lipid absorption | PRAP1/APOA2 | 2 | 0.00238692508579594 | 0.0364079457996288 |
| Biological Process (Gene Ontology) | GO:0010878 | cholesterol storage | CES1/LPL | 2 | 0.00258944734335608 | 0.0375734094107382 |
| Biological Process (Gene Ontology) | GO:0043567 | regulation of insulin-like growth factor receptor signaling pathway | IGFBP2/IGFBP1 | 2 | 0.00258944734335608 | 0.0375734094107382 |
| Biological Process (Gene Ontology) | GO:1903318 | negative regulation of protein maturation | PLAT/FURIN | 2 | 0.00258944734335608 | 0.0375734094107382 |
| Biological Process (Gene Ontology) | GO:0090128 | regulation of synapse maturation | IGSF9/NEFL | 2 | 0.00279979019883251 | 0.0402151683105033 |
| Biological Process (Gene Ontology) | GO:0010875 | positive regulation of cholesterol efflux | CES1/ADIPOQ | 2 | 0.00301790607353183 | 0.0424897270946758 |
| Biological Process (Gene Ontology) | GO:0002227 | innate immune response in mucosa | PLA2G1B/BPIFB1 | 2 | 0.00324374759653721 | 0.0447826124492806 |
| Biological Process (Gene Ontology) | GO:0050996 | positive regulation of lipid catabolic process | FGF21/APOA2 | 2 | 0.00324374759653721 | 0.0447826124492806 |
| Biological Process (Gene Ontology) | GO:0002092 | positive regulation of receptor internalization | SELE/SFRP4 | 2 | 0.00347726760392171 | 0.0475449474305449 |
| Cellular Component (Gene Ontology) | GO:0062023 | collagen-containing extracellular matrix | SSC5D/COL4A1/ADIPOQ/SERPINF1/BCAN/MATN3/ENTPD2/CBLN4/NCAN | 9 | 7.27458072299461e-06 | 0.000865675106036359 |
| Cellular Component (Gene Ontology) | GO:0005788 | endoplasmic reticulum lumen | CES1/IGFBP1/BPIFB2/COL4A1/MATN3/APOA2 | 6 | 0.000440114465865348 | 0.0144806636140937 |
| Cellular Component (Gene Ontology) | GO:0045177 | apical part of cell | IGFBP2/CDHR2/ERBB2/DSG2/CA14/PLAT | 6 | 0.00372832852232239 | 0.0341285457043357 |
| Cellular Component (Gene Ontology) | GO:0005775 | vacuolar lumen | GUSB/CREG1/BCAN/NCAN | 4 | 0.00231321990817062 | 0.0275273169072304 |
| Cellular Component (Gene Ontology) | GO:0005604 | basement membrane | COL4A1/SERPINF1/ENTPD2 | 3 | 0.00345775826732109 | 0.0341285457043357 |
| Cellular Component (Gene Ontology) | GO:0043202 | lysosomal lumen | GUSB/BCAN/NCAN | 3 | 0.00355909443853851 | 0.0341285457043357 |
| Cellular Component (Gene Ontology) | GO:0005796 | Golgi lumen | FURIN/BCAN/NCAN | 3 | 0.0044354981766472 | 0.0376677145773986 |
| Cellular Component (Gene Ontology) | GO:0044853 | plasma membrane raft | SELE/CD8A/SELPLG | 3 | 0.00556532094701515 | 0.0392784000152786 |
| Cellular Component (Gene Ontology) | GO:0072534 | perineuronal net | BCAN/NCAN | 2 | 0.000423769781478054 | 0.0144806636140937 |
| Cellular Component (Gene Ontology) | GO:0098966 | perisynaptic extracellular matrix | BCAN/NCAN | 2 | 0.00051690039609267 | 0.0144806636140937 |
| Cellular Component (Gene Ontology) | GO:0099535 | synapse-associated extracellular matrix | BCAN/NCAN | 2 | 0.00061903471485008 | 0.0144806636140937 |
| Cellular Component (Gene Ontology) | GO:0042627 | chylomicron | LPL/APOA2 | 2 | 0.000730117493147579 | 0.0144806636140937 |
| Cellular Component (Gene Ontology) | GO:0034361 | very-low-density lipoprotein particle | LPL/APOA2 | 2 | 0.00193437610028589 | 0.0275273169072304 |
| Cellular Component (Gene Ontology) | GO:0034385 | triglyceride-rich plasma lipoprotein particle | LPL/APOA2 | 2 | 0.00193437610028589 | 0.0275273169072304 |
| Cellular Component (Gene Ontology) | GO:0140047 | specialized extracellular matrix | BCAN/NCAN | 2 | 0.00212354950735874 | 0.0275273169072304 |
| Cellular Component (Gene Ontology) | GO:0031528 | microvillus membrane | CDHR2/CA9 | 2 | 0.00474803124925193 | 0.0376677145773986 |
| Cellular Component (Gene Ontology) | GO:0034358 | plasma lipoprotein particle | LPL/APOA2 | 2 | 0.00594127059054634 | 0.0392784000152786 |
| Cellular Component (Gene Ontology) | GO:1990777 | lipoprotein particle | LPL/APOA2 | 2 | 0.00594127059054634 | 0.0392784000152786 |
| Cellular Component (Gene Ontology) | GO:0032994 | protein-lipid complex | LPL/APOA2 | 2 | 0.0069166502256489 | 0.0433200724659063 |
| Molecular function (Gene Ontology) | GO:0019838 | growth factor binding | IGFBP2/IGFBP1/ERBB2/WFIKKN2/COL4A1/IL1RN/NTRK3/FURIN | 8 | 9.20739866944834e-09 | 1.85989453122856e-06 |
| Molecular function (Gene Ontology) | GO:0031406 | carboxylic acid binding | FABP4/RBP5/SELE/ADIPOQ/FURIN/PLA2G1B | 6 | 2.46998587674472e-05 | 0.00132435685834375 |
| Molecular function (Gene Ontology) | GO:0043177 | organic acid binding | FABP4/RBP5/SELE/ADIPOQ/FURIN/PLA2G1B | 6 | 2.62248882840346e-05 | 0.00132435685834375 |
| Molecular function (Gene Ontology) | GO:0004857 | enzyme inhibitor activity | IGFBP2/WFIKKN2/FURIN/SERPINF1/APOA2 | 5 | 0.0093986828888387 | 0.0360893912137755 |
| Molecular function (Gene Ontology) | GO:0098632 | cell-cell adhesion mediator activity | DSG2/IGSF9/KRT18/CNTN5 | 4 | 2.55734875826929e-05 | 0.00132435685834375 |
| Molecular function (Gene Ontology) | GO:0098631 | cell adhesion mediator activity | DSG2/IGSF9/KRT18/CNTN5 | 4 | 9.16403237322916e-05 | 0.00370226907878458 |
| Molecular function (Gene Ontology) | GO:0016829 | lyase activity | PTS/CA14/CA9/DDC | 4 | 0.00347245417044069 | 0.0175358935607255 |
| Molecular function (Gene Ontology) | GO:0005125 | cytokine activity | IL1RN/ADIPOQ/INHBC/IL32 | 4 | 0.00604583828183555 | 0.0259842411261868 |
| Molecular function (Gene Ontology) | GO:0005539 | glycosaminoglycan binding | LPL/FURIN/BCAN/NCAN | 4 | 0.00679635563216769 | 0.028601329952039 |
| Molecular function (Gene Ontology) | GO:0030246 | carbohydrate binding | GUSB/SELE/BCAN/NCAN | 4 | 0.0112051877178144 | 0.0419120982934105 |
| Molecular function (Gene Ontology) | GO:0030547 | signaling receptor inhibitor activity | IGFBP2/WFIKKN2/IL1RN | 3 | 0.000345891136550491 | 0.00448731567491554 |
| Molecular function (Gene Ontology) | GO:0005004 | GPI-linked ephrin receptor activity | MET/ERBB2/NTRK3 | 3 | 0.000475898772828306 | 0.00448731567491554 |
| Molecular function (Gene Ontology) | GO:0005008 | hepatocyte growth factor receptor activity | MET/ERBB2/NTRK3 | 3 | 0.000475898772828306 | 0.00448731567491554 |
| Molecular function (Gene Ontology) | GO:0005009 | insulin receptor activity | MET/ERBB2/NTRK3 | 3 | 0.000475898772828306 | 0.00448731567491554 |
| Molecular function (Gene Ontology) | GO:0005011 | macrophage colony-stimulating factor receptor activity | MET/ERBB2/NTRK3 | 3 | 0.000475898772828306 | 0.00448731567491554 |
| Molecular function (Gene Ontology) | GO:0005018 | platelet-derived growth factor alpha-receptor activity | MET/ERBB2/NTRK3 | 3 | 0.000475898772828306 | 0.00448731567491554 |
| Molecular function (Gene Ontology) | GO:0005020 | stem cell factor receptor activity | MET/ERBB2/NTRK3 | 3 | 0.000475898772828306 | 0.00448731567491554 |
| Molecular function (Gene Ontology) | GO:0008288 | boss receptor activity | MET/ERBB2/NTRK3 | 3 | 0.000475898772828306 | 0.00448731567491554 |
| Molecular function (Gene Ontology) | GO:0036332 | placental growth factor receptor activity | MET/ERBB2/NTRK3 | 3 | 0.000475898772828306 | 0.00448731567491554 |
| Molecular function (Gene Ontology) | GO:0038062 | protein tyrosine kinase collagen receptor activity | MET/ERBB2/NTRK3 | 3 | 0.000475898772828306 | 0.00448731567491554 |
| Molecular function (Gene Ontology) | GO:0060175 | brain-derived neurotrophic factor receptor activity | MET/ERBB2/NTRK3 | 3 | 0.000475898772828306 | 0.00448731567491554 |
| Molecular function (Gene Ontology) | GO:0005006 | epidermal growth factor receptor activity | MET/ERBB2/NTRK3 | 3 | 0.000505160200914847 | 0.00448731567491554 |
| Molecular function (Gene Ontology) | GO:0005007 | fibroblast growth factor receptor activity | MET/ERBB2/NTRK3 | 3 | 0.000505160200914847 | 0.00448731567491554 |
| Molecular function (Gene Ontology) | GO:0005017 | platelet-derived growth factor receptor activity | MET/ERBB2/NTRK3 | 3 | 0.000505160200914847 | 0.00448731567491554 |
| Molecular function (Gene Ontology) | GO:0005019 | platelet-derived growth factor beta-receptor activity | MET/ERBB2/NTRK3 | 3 | 0.000505160200914847 | 0.00448731567491554 |
| Molecular function (Gene Ontology) | GO:0005010 | insulin-like growth factor receptor activity | MET/ERBB2/NTRK3 | 3 | 0.000535550670869971 | 0.00448731567491554 |
| Molecular function (Gene Ontology) | GO:0005021 | vascular endothelial growth factor receptor activity | MET/ERBB2/NTRK3 | 3 | 0.000535550670869971 | 0.00448731567491554 |
| Molecular function (Gene Ontology) | GO:0005030 | neurotrophin receptor activity | MET/ERBB2/NTRK3 | 3 | 0.000535550670869971 | 0.00448731567491554 |
| Molecular function (Gene Ontology) | GO:0005003 | ephrin receptor activity | MET/ERBB2/NTRK3 | 3 | 0.000599789718924354 | 0.00448731567491554 |
| Molecular function (Gene Ontology) | GO:0005005 | transmembrane-ephrin receptor activity | MET/ERBB2/NTRK3 | 3 | 0.000599789718924354 | 0.00448731567491554 |
| Molecular function (Gene Ontology) | GO:0038064 | collagen receptor activity | MET/ERBB2/NTRK3 | 3 | 0.000599789718924354 | 0.00448731567491554 |
| Molecular function (Gene Ontology) | GO:0004714 | transmembrane receptor protein tyrosine kinase activity | MET/ERBB2/NTRK3 | 3 | 0.000862745788951508 | 0.00600947066786913 |
| Molecular function (Gene Ontology) | GO:0016500 | protein-hormone receptor activity | MET/ERBB2/NTRK3 | 3 | 0.00118929851736851 | 0.00774962259704639 |
| Molecular function (Gene Ontology) | GO:0019199 | transmembrane receptor protein kinase activity | MET/ERBB2/NTRK3 | 3 | 0.00184353364765769 | 0.0110355350058875 |
| Molecular function (Gene Ontology) | GO:0016835 | carbon-oxygen lyase activity | PTS/CA14/CA9 | 3 | 0.00191209764953496 | 0.0110355350058875 |
| Molecular function (Gene Ontology) | GO:0033293 | monocarboxylic acid binding | FABP4/RBP5/PLA2G1B | 3 | 0.00191209764953496 | 0.0110355350058875 |
| Molecular function (Gene Ontology) | GO:0004867 | serine-type endopeptidase inhibitor activity | WFIKKN2/FURIN/SERPINF1 | 3 | 0.00323128215470052 | 0.016736384493577 |
| Molecular function (Gene Ontology) | GO:0035401 | histone H3Y41 kinase activity | MET/ERBB2/NTRK3 | 3 | 0.00551683292702541 | 0.0242260924186768 |
| Molecular function (Gene Ontology) | GO:0140801 | histone H2AXY142 kinase activity | MET/ERBB2/NTRK3 | 3 | 0.00551683292702541 | 0.0242260924186768 |
| Molecular function (Gene Ontology) | GO:0016298 | lipase activity | CES1/LPL/PLA2G1B | 3 | 0.00773583649824082 | 0.0318905912784622 |
| Molecular function (Gene Ontology) | GO:0005179 | hormone activity | ADIPOQ/INHBC/GUCA2A | 3 | 0.00806594940695313 | 0.0325864356040907 |
| Molecular function (Gene Ontology) | GO:0004713 | protein tyrosine kinase activity | MET/ERBB2/NTRK3 | 3 | 0.00946899868480248 | 0.0360893912137755 |
| Molecular function (Gene Ontology) | GO:0019842 | vitamin binding | RBP5/AFM/DDC | 3 | 0.0114117099313741 | 0.0419120982934105 |
| Molecular function (Gene Ontology) | GO:0004866 | endopeptidase inhibitor activity | WFIKKN2/FURIN/SERPINF1 | 3 | 0.0137959021920879 | 0.0497637900500314 |
| Molecular function (Gene Ontology) | GO:0043121 | neurotrophin binding | NTRK3/FURIN | 2 | 0.000421436206559631 | 0.00448731567491554 |
| Molecular function (Gene Ontology) | GO:0031994 | insulin-like growth factor I binding | IGFBP2/IGFBP1 | 2 | 0.00072611661352033 | 0.00523841271182524 |
| Molecular function (Gene Ontology) | GO:0004089 | carbonate dehydratase activity | CA14/CA9 | 2 | 0.000973562015987835 | 0.00655531757431809 |
| Molecular function (Gene Ontology) | GO:0005520 | insulin-like growth factor binding | IGFBP2/IGFBP1 | 2 | 0.00157288785404059 | 0.00992885457863125 |
| Molecular function (Gene Ontology) | GO:0033691 | sialic acid binding | SELE/ADIPOQ | 2 | 0.00211208344372507 | 0.0118511348786796 |
| Molecular function (Gene Ontology) | GO:0043274 | phospholipase binding | SELE/NEFL | 2 | 0.00230862006942772 | 0.0126038176763351 |
| Molecular function (Gene Ontology) | GO:0005540 | hyaluronic acid binding | BCAN/NCAN | 2 | 0.00251347209012339 | 0.013361088479077 |
| Molecular function (Gene Ontology) | GO:0048019 | receptor antagonist activity | WFIKKN2/IL1RN | 2 | 0.00391432062376286 | 0.0192851894146365 |
| Molecular function (Gene Ontology) | GO:0017147 | Wnt-protein binding | FRZB/SFRP4 | 2 | 0.00417594181015791 | 0.0200842915631404 |
| Molecular function (Gene Ontology) | GO:0071813 | lipoprotein particle binding | LPL/APOA2 | 2 | 0.00444547012011426 | 0.0204087491877973 |
| Molecular function (Gene Ontology) | GO:0071814 | protein-lipid complex binding | LPL/APOA2 | 2 | 0.00444547012011426 | 0.0204087491877973 |
| Molecular function (Gene Ontology) | GO:0005504 | fatty acid binding | FABP4/RBP5 | 2 | 0.0094037757971564 | 0.0360893912137755 |
| KEGG Pathways | hsa03320 | PPAR signaling pathway (Endocrine system) | FABP4/APOA2/LPL/ADIPOQ | 4 | 0.000227452110892795 | 0.0277491575289209 |

**Supplementary Table 4. Biological curation of top-ten LFC-related plasma proteins. ***

| **Protein** | **Protein name** | **LASSO directionality** | **Molecular evidence**† |
| --- | --- | --- | --- |
| IGFBP2 | Insulin-like growth factor-binding protein 2 | - | Inversely associated with liver fat accumulation and positively associated with higher insulin sensitivity, and a more favorable lipid profile [15, 16] |
| FABP4 | Fatty acid-binding protein, adipocyte | + | Regulated lipid storage and oxidation; Increases adipose tissue and systemic inflammation; Worsened insulin sensitivity; Promoted atherosclerosis development; Increases endothelial dysfunction [20] |
| MET | Hepatocyte growth factor receptor | - | Alleviated hepatic insulin resistance and lipid accumulation [21] |
| CPM | Carboxypeptidase M | + | Involved in apoptosis, adipogenesis and cancer [24] |
| CES1 | Liver carboxylesterase 1 | + | Promotes lipid storage, insulin resistance and NAFLD fibrosis; participated in obesity-induced hepatic steatosis [17] |
| IGFBP1 | Insulin-like growth factor-binding protein 1 | - | A hepatoprotective factor that prevents apoptosis in hepatocytes [25]; decreased susceptibility to obesity and improve insulin sensitivity [18,19] |
| CDHR2 | Cadherin-related family member 2 | + | A tumor suppressor in hepatocellular carcinoma [26] |
| RBP5 | Retinol-binding protein 5 | + | Pro-inflammation cytokines |
| ERBB2 | Receptor tyrosine-protein kinase erbB-2 | + | Associated with insulin resistance and lipid metabolism in HER2-positive breast cancer [27] |
| SSC5D | Soluble scavenger receptor cysteine-rich domain-containing protein SSC5D | + | Drived immune responses and inflammation [28]; a hepatic fibrosis biomarker [29] |

*The top-ten LFC-related plasma proteins (LASSO regression) were examined via literature search to assess potential implications in cardiometabolic health.

† The corresponding references are placed in the reference list of the main text.

**Abbreviations:** LFC, liver fat content; LASSO, least absolute shrinkage and selection operator.

**Supplementary Table 5. The association between the 62-protein LFC score and 10-protein LFC score with 14 cardiovascular-kidney-metabolic (CKM) outcomes.**

| **Outcomes** | **Event (%)** | **The 62-protein LFC score** | | | **The 10-protein LFC score** | | |
| --- | --- | --- | --- | --- | --- | --- | --- |
|  |  | **Crude Model HR (95 CI %)** | **Model 1**  **HR (95 CI %)** | **Model 2**  **HR (95 CI %)** | **Crude Model HR (95 CI %)** | **Model 1**  **HR (95 CI %)** | **Model 2**  **HR (95 CI %)** |
| Hypertension | 11919(26.2) | 1.52(1.49,1.54) | 1.49(1.46,1.52) | 1.17(1.14,1.20) | 1.49(1.46,1.51) | 1.47(1.44,1.49) | 1.15(1.12,1.18) |
| CHD | 3821(8.4) | 1.41(1.37,1.45) | 1.32(1.28,1.37) | 1.19(1.14,1.24) | 1.36(1.32,1.40) | 1.30(1.26,1.34) | 1.16(1.11,1.21) |
| Stroke | 1395(3.1) | 1.17(1.11,1.23) | 1.10(1.04,1.16) | 1.02(0.95,1.10) | 1.14(1.08,1.20) | 1.08(1.02,1.14) | 1.00(0.93,1.08) |
| MASLD | 563(1.2) | 2.59(2.40,2.79) | 2.62(2.42,2.82) | 2.64(2.38,2.92) | 2.50(2.31,2.70) | 2.51(2.32,2.71) | 2.38(2.15,2.64) |
| Cirrhosis | 482(1.1) | 1.54(1.41,1.67) | 1.50(1.38,1.64) | 1.61(1.43,1.82) | 1.49(1.37,1.62) | 1.46(1.34,1.59) | 1.53(1.36,1.72) |
| Liver failure | 93(0.2) | 1.94(1.61,2.34) | 1.86(1.52,2.26) | 2.21(1.70,2.87) | 1.85(1.52,2.24) | 1.78(1.45,2.17) | 2.05(1.58,2.67) |
| Hepatocellular carcinoma | 33(0.1) | 1.87(1.37,2.56) | 1.85(1.33,2.59) | 1.82(1.16,2.85) | 1.88(1.36,2.59) | 1.87(1.34,2.62) | 1.86(1.19,2.90) |
| Gout | 761(1.7) | 2.09(1.95,2.23) | 1.95(1.82,2.10) | 1.68(1.53,1.85) | 2.07(1.93,2.21) | 1.99(1.85,2.13) | 1.70(1.54,1.87) |
| Kidney stones | 609(1.3) | 1.52(1.41,1.64) | 1.44(1.33,1.56) | 1.41(1.27,1.57) | 1.48(1.37,1.60) | 1.42(1.31,1.53) | 1.38(1.24,1.53) |
| Chronic kidney disease | 1722(3.8) | 1.36(1.30,1.42) | 1.34(1.27,1.40) | 1.05(0.99,1.12) | 1.42(1.36,1.49) | 1.41(1.35,1.48) | 1.18(1.11,1.25) |
| Acute kidney injury | 2106(4.6) | 1.43(1.37,1.48) | 1.37(1.31,1.43) | 1.12(1.06,1.19) | 1.39(1.34,1.45) | 1.35(1.29,1.41) | 1.11(1.05,1.18) |
| Type 2 diabetes | 2757(6.1) | 2.89(2.79,3.00) | 2.93(2.83,3.04) | 2.30(2.20,2.41) | 2.81(2.71,2.92) | 2.85(2.74,2.96) | 2.19(2.09,2.30) |
| COPD | 2233(4.9) | 1.29(1.24,1.34) | 1.25(1.19,1.30) | 1.19(1.13,1.26) | 1.21(1.16,1.26) | 1.17(1.12,1.22) | 1.12(1.06,1.19) |
| Obstructive sleep apnea | 679(1.5) | 2.03(1.90,2.18) | 1.95(1.82,2.10) | 1.29(1.17,1.42) | 2.04(1.90,2.19) | 1.97(1.83,2.12) | 1.33(1.20,1.46) |

Model 1: Adjusted for age, and sex.

Model 2: Adjusted for age, sex, race, BMI, Townsend deprivation index, smoking, alcohol, systolic blood pressure, LDL, and HbA1c.

**Abbreviations**: LFC, liver fat content; COPD, chronic obstructive pulmonary disease; BMI, body mass index; MASLD, metabolic dysfunction-associated steatotic liver disease; CHD, coronary heart disease; HbA1c, glycosylated hemoglobin A1c; LDL-C, low-density lipoprotein-cholesterol.

**Supplementary Table 6. Stratified analyses for association between the 62-protein LFC score and 10-protein LFC score with risk of stroke. ***

| **Variable** | **The 62-protein LFC score** | | **The 10-protein LFC score** | |
| --- | --- | --- | --- | --- |
|  | **HR (95%CI)** | ***P* interaction** | **HR (95%CI)** | ***P* interaction** |
| **Age, years** |  | <0.001 |  | <0.001 |
| <57.0 [Median] | 1.21(1.07,1.35) |  | 1.17(1.05,1.32) |  |
| ≥57.0 | 0.95(0.87,1.02) |  | 0.93(0.86,1.01) |  |
| **Sex** |  | 0.943 |  | 0.932 |
| Female | 1.02(0.93,1.12) |  | 1.00(0.91,1.10) |  |
| Male | 1.02(0.93,1.11) |  | 1.00(0.92,1.10) |  |
| **Race** |  | 0.473 |  | 0.762 |
| White | 1.01(0.94,1.09) |  | 1.00(0.93,1.08) |  |
| Non-white | 1.11(0.86,1.44) |  | 1.04(0.80,1.34) |  |
| **HbA1c, mmol/L** |  | 0.419 |  | 0.456 |
| <35.1 [Median] | 1.01(0.91,1.13) |  | 1.00(0.90,1.11) |  |
| ≥35.1 | 1.06(0.98,1.16) |  | 1.04(0.96,1.14) |  |
| **LDL-C, mmol/L** |  | 0.049 |  | 0.054 |
| <3.6 [Median] | 0.97(0.89,1.06) |  | 0.96(0.87,1.04) |  |
| ≥3.6 | 1.08(0.99,1.19) |  | 1.06(0.97,1.17) |  |
| **Systolic blood pressure, mmHg** |  | 0.312 |  | 0.232 |
| <135.5 [Median] | 1.07(0.97,1.20) |  | 1.06(0.96,1.18) |  |
| ≥135.5 | 1.01(0.93,1.10) |  | 0.99(0.91,1.08) |  |
| **BMI, kg/m^2^** |  | 0.298 |  | 0.276 |
| <26.5 [Median] | 1.08(0.97,1.20) |  | 1.07(0.96,1.18) |  |
| ≥26.5 | 1.00(0.92,1.09) |  | 0.99(0.91,1.08) |  |

*All models were adjusted, if not stratified, for age, sex, race, BMI, Townsend deprivation index, smoking, alcohol, systolic blood pressure, LDL, and HbA1c.

**Abbreviations:** LFC, liver fat content; BMI, body mass index; HbA1c, glycosylated hemoglobin A1c; LDL-C, low-density lipoprotein-cholesterol.

**Supplementary Table 7. The association between the 62-protein LFC score and 10-protein LFC score with 14 cardiovascular-kidney-metabolic (CKM) outcomes ***

| **Outcomes** | **The 62-protein LFC score** | | | | | **The 10-protein LFC score** | | | | |
| --- | --- | --- | --- | --- | --- | --- | --- | --- | --- | --- |
|  | **Multiple Imputation^†^ HR (95 CI %)** | **Complete-case Analysis**^‡^  **HR (95 CI %)** | **Fine-Gray Model^§^**  **sHR (95 CI %)** | **GPS-IPW^‖^**  **HR (95 CI %)** | **GPS-Adjusted^¶^**  **HR (95 CI %)** | **Multiple Imputation^†^ HR (95 CI %)** | **Complete-case Analysis**^‡^  **HR (95 CI %)** | **Fine-Gray Model^§^**  **sHR (95 CI %)** | **GPS-IPW^‖^**  **HR (95 CI %)** | **GPS-Adjusted^¶^**  **HR (95 CI %)** |
| Hypertension | 1.17(1.14,1.20) | 1.18(1.14,1.21) | 1.20 (1.17-1.23) | 1.32(1.29,1.35) | 1.17(1.14,1.20) | 1.15(1.12,1.18) | 1.15(1.11,1.19) | 1.18 (1.14-1.21) | 1.28(1.25,1.32) | 1.15(1.12,1.18) |
| CHD | 1.20(1.14,1.25) | 1.18(1.11,1.25) | 1.22 (1.17-1.29) | 1.28(1.23,1.33) | 1.19(1.14,1.24) | 1.17(1.12,1.22) | 1.15(1.09,1.22) | 1.19 (1.14-1.25) | 1.23(1.18,1.28) | 1.16(1.11,1.21) |
| Stroke | 1.01(0.94,1.09) | 0.97(0.88,1.07) | 1.10 (1.01-1.20) | 1.04(0.97,1.12) | 1.02(0.95,1.10) | 1.00(0.93,1.07) | 0.97(0.88,1.06) | 1.06 (0.98-1.15) | 1.01(0.94,1.08) | 1.00(0.93,1.08) |
| MASLD | 2.74(2.46,3.04) | 2.92(2.54,3.35) | 2.82 (2.53-3.15) | 2.66(2.42,2.92) | 2.64(2.38,2.92) | 2.42(2.18,2.69) | 2.56(2.23,2.93) | 2.53 (2.28-2.81) | 2.45(2.22,2.70) | 2.38(2.15,2.64) |
| Cirrhosis | 1.62(1.43,1.82) | 1.56(1.33,1.84) | 1.60 (1.30-1.95) | 1.58(1.41,1.77) | 1.61(1.43,1.82) | 1.53(1.36,1.72) | 1.45(1.23,1.70) | 1.52 (1.26-1.84) | 1.50(1.34,1.68) | 1.53(1.36,1.72) |
| Liver failure | 2.21(1.69,2.88) | 2.25(1.56,3.24) | 3.45 (1.95-6.13) | 2.03(1.57,2.63) | 2.21(1.70,2.87) | 2.05(1.57,2.67) | 2.13(1.48,3.08) | 3.35 (1.85-6.04) | 1.94(1.52,2.49) | 2.05(1.58,2.67) |
| Hepatocellular carcinoma | 1.99(1.26,3.13) | 0.98(0.44,2.18) | 1.84 (0.67-5.10) | 1.77(1.20,2.60) | 1.82(1.16,2.85) | 1.96(1.25,3.07) | 1.03(0.47,2.23) | 2.07 (0.79-5.40) | 1.74(1.13,2.68) | 1.86(1.19,2.90) |
| Gout | 1.71(1.55,1.88) | 1.80(1.59,2.04) | 1.87 (1.68-2.09) | 1.85(1.67,2.04) | 1.68(1.53,1.85) | 1.71(1.56,1.89) | 1.81(1.59,2.05) | 1.87 (1.68-2.08) | 1.76(1.60,1.94) | 1.70(1.54,1.87) |
| Kidney stones | 1.41(1.27,1.57) | 1.44(1.25,1.65) | 1.45 (1.29-1.62) | 1.43(1.29,1.59) | 1.41(1.27,1.57) | 1.38(1.24,1.53) | 1.39(1.21,1.60) | 1.40 (1.25-1.57) | 1.40(1.26,1.56) | 1.38(1.24,1.53) |
| Chronic kidney disease | 1.06(0.99,1.12) | 1.05(0.96,1.15) | 1.06 (0.99-1.14) | 1.19(1.12,1.26) | 1.05(0.99,1.12) | 1.17(1.10,1.25) | 1.16(1.07,1.27) | 1.18 (1.10-1.27) | 1.25(1.18,1.33) | 1.18(1.11,1.25) |
| Acute kidney injury | 1.12(1.06,1.19) | 1.18(1.09,1.27) | 1.21 (1.13-1.31) | 1.28(1.21,1.36) | 1.12(1.06,1.19) | 1.11(1.05,1.18) | 1.17(1.09,1.27) | 1.21 (1.12-1.30) | 1.24(1.17,1.32) | 1.11(1.05,1.18) |
| Type 2 diabetes | 2.34(2.23,2.45) | 2.27(2.14,2.42) | 2.38 (2.25-2.53) | 2.31(2.18,2.44) | 2.30(2.20,2.41) | 2.23(2.13,2.34) | 2.17(2.04,2.31) | 2.23 (2.10-2.36) | 2.22(2.10,2.35) | 2.19(2.09,2.30) |
| COPD | 1.19(1.12,1.26) | 1.26(1.17,1.36) | 1.17 (1.09-1.25) | 1.23(1.17,1.30) | 1.19(1.13,1.26) | 1.11(1.05,1.18) | 1.18(1.10,1.27) | 1.11 (1.03-1.19) | 1.16(1.10,1.23) | 1.12(1.06,1.19) |
| Obstructive sleep apnea | 1.30(1.18,1.43) | 1.29(1.14,1.48) | 1.31 (1.18-1.45) | 1.66(1.52,1.82) | 1.29(1.17,1.42) | 1.33(1.21,1.46) | 1.31(1.15,1.49) | 1.35 (1.21-1.49) | 1.68(1.52,1.85) | 1.33(1.20,1.46) |

*All models were adjusted for age, sex, race, BMI, Townsend deprivation index, smoking, alcohol, systolic blood pressure, LDL, and HbA1c.

**†** Multiple Imputation: Results from multiple imputation by chained equations (MICE) analysis.

‡Fine-Gray Model: Subdistribution hazard ratios (sHR) from Fine-Gray proportional hazards models, accounting for death as a competing risk.

§Complete-case Analysis (n=26168): Hazard ratios from Cox regression models using unimputed data.

‖ GPS-IPW: Hazard ratios from generalized propensity score inverse probability weighted Cox regression.

¶ GPS-Adjusted: Hazard ratios from Cox regression with generalized propensity score included as a covariate.

**Abbreviations**: LFC, liver fat content; COPD, chronic obstructive pulmonary disease; BMI, body mass index; MASLD, metabolic dysfunction-associated steatotic liver disease; CHD, coronary heart disease; HbA1c, glycosylated hemoglobin A1c; LDL-C, low-density lipoprotein-cholesterol.

**Supplementary Table 8. Categorical net reclassification improvement (NRI) of the 62- and 10-protein LFC score for** **14 cardiovascular-kidney-metabolic (CKM) outcomes beyond standard risk factors.**

| **Outcomes** | **Categorical NRI of the 62-protein LFC score** | | | **Categorical NRI of the 10-protein LFC score** | | |
| --- | --- | --- | --- | --- | --- | --- |
|  | **Overall** | **Events** | **Non-Events** | **Overall** | **Events** | **Non-Events** |
| Hypertension | 0.005 (0.001, 0.010) | 0.001 (-0.002, 0.005) | 0.004 (0.002, 0.008) | 0.004 (-0.000, 0.009) | -0.000 (-0.003, 0.004) | 0.004 (0.001, 0.007) |
| CHD | 0.007 (-0.002, 0.014) | 0.002 (-0.005, 0.009) | 0.005 (0.001, 0.008) | 0.002 (-0.004, 0.010) | -0.001 (-0.006, 0.007) | 0.003 (-0.000, 0.005) |
| Stroke | 0.000 (-0.008, 0.007) | 0.000 (-0.009, 0.007) | 0.000 (-0.001, 0.002) | 0.001 (-0.008, 0.008) | 0.001 (-0.009, 0.007) | -0.000 (-0.001, 0.002) |
| MASLD | 0.148 (0.085, 0.210) | 0.096 (0.028, 0.164) | 0.053 (0.020, 0.086) | 0.124 (0.065, 0.189) | 0.082 (0.020, 0.151) | 0.042 (0.013, 0.073) |
| Cirrhosis | 0.049 (-0.020, 0.066) | 0.013 (-0.056, 0.029) | 0.037 (0.018, 0.058) | 0.051 (-0.017, 0.066) | 0.022 (-0.045, 0.036) | 0.029 (0.014, 0.049) |
| Liver failure | 0.059 (-0.060, 0.195) | 0.018 (-0.097, 0.149) | 0.041 (0.009, 0.088) | 0.013 (-0.064, 0.180) | -0.020 (-0.102, 0.141) | 0.033 (0.006, 0.081) |
| Hepatocellular carcinoma | 0.078 (-0.147, 0.169) | 0.049 (-0.167, 0.147) | 0.029 (0.000, 0.065) | 0.080 (-0.145, 0.199) | 0.049 (-0.175, 0.164) | 0.031 (0.001, 0.071) |
| Gout | 0.020 (0.003, 0.055) | 0.000 (-0.014, 0.034) | 0.020 (0.012, 0.029) | 0.021 (0.004, 0.058) | -0.000 (-0.014, 0.036) | 0.021 (0.013, 0.030) |
| Kidney stones | 0.010 (-0.010, 0.061) | -0.009 (-0.033, 0.031) | 0.019 (0.001, 0.052) | 0.020 (-0.011, 0.056) | 0.004 (-0.033, 0.030) | 0.016 (-0.001, 0.047) |
| Chronic kidney disease | -0.001 (-0.006, 0.010) | -0.001 (-0.007, 0.009) | 0.000 (-0.001, 0.002) | -0.002 (-0.008, 0.016) | -0.004 (-0.011, 0.013) | 0.002 (0.000, 0.006) |
| Acute kidney injury | 0.002 (-0.007, 0.015) | 0.001 (-0.007, 0.013) | 0.001 (-0.001, 0.003) | 0.003 (-0.007, 0.014) | 0.002 (-0.007, 0.013) | 0.002 (-0.001, 0.003) |
| Type 2 diabetes | 0.076 (0.057, 0.091) | 0.003 (-0.008, 0.019) | 0.073 (0.055, 0.082) | 0.064 (0.049, 0.081) | 0.001 (-0.009, 0.016) | 0.063 (0.050, 0.072) |
| COPD | 0.006 (-0.004, 0.016) | 0.004 (-0.006, 0.013) | 0.002 (0.000, 0.006) | 0.005 (-0.005, 0.012) | 0.004 (-0.006, 0.011) | 0.001 (-0.001, 0.003) |
| Obstructive sleep apnea | 0.014 (-0.003, 0.039) | -0.012 (-0.024, 0.011) | 0.026 (0.011, 0.037) | 0.018 (-0.003, 0.043) | -0.012 (-0.030, 0.010) | 0.030 (0.014, 0.041) |

The categorical NRI was calculated using risk thresholds based on half of the cumulative incidence for each outcome. The 62-protein LFC score and 10-protein LFC score were added to the standard risk factors model to assess improvement in risk reclassification. Standard risk factors: age, sex, race, BMI, Townsend deprivation index, smoking status, alcohol use, systolic blood pressure, LDL cholesterol, and HbA1c.

**Abbreviations**: CHD, coronary heart disease; CKM, cardiovascular-kidney-metabolic; COPD, chronic obstructive pulmonary disease; FLI, fatty liver index; LFC, liver-function composite; MASLD, metabolic dysfunction-associated steatotic liver disease; NRI, net reclassification improvement.

**Supplementary Table 9. Categorical net reclassification improvement (NRI) of the 62- and 10-protein LFC score for 14 cardiovascular-kidney-metabolic (CKM) outcomes beyond FLI.**

| **Outcomes** | **Categorical NRI of the 62-protein LFC score** | | | **Categorical NRI of the 10-protein LFC score** | | |
| --- | --- | --- | --- | --- | --- | --- |
|  | **Overall** | **Events** | **Non-Events** | **Overall** | **Events** | **Non-Events** |
| Hypertension | 0.010 (0.006, 0.015) | 0.001 (-0.002, 0.005) | 0.009 (0.006, 0.011) | 0.006 (0.002, 0.011) | 0.000 (-0.003, 0.004) | 0.006 (0.003, 0.009) |
| CHD | 0.004 (-0.002, 0.015) | -0.000 (-0.005, 0.010) | 0.004 (0.000, 0.006) | 0.002 (-0.005, 0.009) | -0.001 (-0.006, 0.007) | 0.003 (-0.001, 0.004) |
| Stroke | -0.002 (-0.007, 0.008) | -0.001 (-0.007, 0.008) | -0.001 (-0.002, 0.002) | 0.001 (-0.008, 0.007) | 0.001 (-0.008, 0.007) | 0.000 (-0.001, 0.003) |
| MASLD | 0.100 (0.052, 0.149) | 0.061 (0.018, 0.106) | 0.039 (0.025, 0.052) | 0.107 (0.057, 0.149) | 0.082 (0.034, 0.121) | 0.025 (0.013, 0.037) |
| Cirrhosis | 0.007 (-0.018, 0.046) | -0.009 (-0.035, 0.027) | 0.016 (0.004, 0.034) | 0.013 (-0.028, 0.036) | 0.003 (-0.040, 0.025) | 0.010 (0.001, 0.025) |
| Liver failure | 0.038 (-0.055, 0.127) | 0.000 (-0.093, 0.081) | 0.037 (0.008, 0.073) | 0.026 (-0.048, 0.137) | 0.000 (-0.083, 0.102) | 0.026 (0.002, 0.057) |
| Hepatocellular carcinoma | 0.062 (-0.163, 0.172) | 0.053 (-0.192, 0.147) | 0.009 (-0.001, 0.062) | 0.012 (-0.156, 0.149) | 0.002 (-0.184, 0.126) | 0.010 (-0.001, 0.060) |
| Gout | -0.002 (-0.013, 0.022) | -0.008 (-0.022, 0.016) | 0.007 (0.003, 0.012) | -0.002 (-0.015, 0.025) | -0.010 (-0.023, 0.015) | 0.008 (0.004, 0.014) |
| Kidney stones | 0.027 (-0.001, 0.077) | 0.013 (-0.018, 0.054) | 0.014 (-0.005, 0.041) | 0.016 (-0.003, 0.066) | 0.007 (-0.018, 0.049) | 0.009 (-0.008, 0.035) |
| Chronic kidney disease | 0.006 (-0.004, 0.010) | 0.005 (-0.005, 0.009) | 0.001 (-0.001, 0.002) | 0.006 (-0.004, 0.018) | 0.005 (-0.007, 0.014) | 0.002 (-0.000, 0.006) |
| Acute kidney injury | 0.006 (-0.002, 0.019) | 0.007 (-0.002, 0.018) | -0.001 (-0.002, 0.003) | 0.005 (-0.005, 0.013) | 0.006 (-0.005, 0.012) | -0.001 (-0.002, 0.002) |
| Type 2 diabetes | 0.068 (0.055, 0.085) | 0.015 (0.004, 0.031) | 0.053 (0.045, 0.061) | 0.064 (0.050, 0.082) | 0.020 (0.010, 0.035) | 0.043 (0.035, 0.051) |
| COPD | 0.008 (-0.003, 0.016) | 0.003 (-0.007, 0.010) | 0.005 (0.001, 0.008) | 0.001 (-0.005, 0.006) | 0.001 (-0.006, 0.005) | 0.000 (-0.001, 0.002) |
| Obstructive sleep apnea | 0.010 (-0.010, 0.029) | 0.010 (-0.011, 0.031) | -0.000 (-0.006, 0.007) | 0.004 (-0.012, 0.032) | 0.004 (-0.015, 0.033) | 0.000 (-0.006, 0.008) |

The categorical NRI was calculated using risk thresholds based on half of the cumulative incidence for each outcome. The 62-protein LFC score and 10-protein LFC score were added to the FLI model to assess improvement in risk reclassification.

**Abbreviations**: CHD, coronary heart disease; CKM, cardiovascular-kidney-metabolic; COPD, chronic obstructive pulmonary disease; FLI, fatty liver index; LFC, liver-function composite; MASLD, metabolic dysfunction-associated steatotic liver disease; NRI, net reclassification improvement.

**Supplementary Table 10. Predictive performance of the 62-protein LFC score over the FLI model for various clinical outcomes.**

| **Outcomes** | **C-index**  **Age + sex+ FLI** | **C-index**  **Age +sex + FLI + 62-protein LFC score** | ***P* value** | **NRI overall** |
| --- | --- | --- | --- | --- |
| Hypertension | 0.701(0.696,0.705) | 0.704(0.700,0.709) | <0.001 | 0.071(0.057,0.084) |
| CHD | 0.712(0.704,0.720) | 0.715(0.707,0.722) | 0.001 | 0.064(0.039,0.085) |
| Stroke | 0.708(0.694,0.721) | 0.708(0.695,0.721) | 0.719 | 0.005(-0.045,0.042) |
| MASLD | 0.743(0.723,0.763) | 0.784(0.765,0.803) | <0.001 | 0.222(0.168,0.284) |
| Cirrhosis | 0.664(0.640,0.689) | 0.672(0.648,0.697) | 0.049 | 0.082(0.022,0.142) |
| Liver failure | 0.697(0.644,0.750) | 0.724(0.674,0.775) | 0.024 | 0.241(0.082,0.354) |
| Hepatocellular carcinoma | 0.750(0.656,0.843) | 0.751(0.659,0.843) | 0.943 | 0.086(-0.163,0.362) |
| Gout | 0.815(0.801,0.829) | 0.821(0.807,0.835) | <0.001 | 0.103(0.056,0.147) |
| Kidney stones | 0.631(0.608,0.653) | 0.648(0.627,0.670) | <0.001 | 0.104(0.056,0.163) |
| Chronic kidney disease | 0.749(0.738,0.761) | 0.750(0.739,0.761) | 0.367 | 0.028(-0.026,0.062) |
| Acute kidney injury | 0.721(0.710,0.732) | 0.723(0.712,0.734) | 0.022 | 0.040(0.005,0.073) |
| Type 2 diabetes | 0.773(0.764,0.781) | 0.812(0.804,0.819) | <0.001 | 0.276(0.250,0.304) |
| COPD | 0.685(0.674,0.695) | 0.688(0.678,0.699) | 0.002 | 0.066(0.036,0.085) |
| Obstructive sleep apnea | 0.742(0.723,0.761) | 0.744(0.725,0.763) | 0.326 | 0.042(-0.014,0.080) |

**Abbreviations:** LFC, liver fat content; FLI, fatty liver index; COPD, chronic obstructive pulmonary disease; MASLD, metabolic dysfunction-associated steatotic liver disease; CHD, coronary heart disease; NRI, net reclassification improvement.

**Supplementary Table 11. Enhanced predictive performance of the 62- and 10-protein LFC score for 14 cardiovascular-kidney-metabolic (CKM) outcomes beyond FLI using multiple imputation.**

| **Outcomes** | **C-index** | | ***P***  **value** | **NRI overall** | **C-index** | | ***P***  **value** | **NRI overall** |
| --- | --- | --- | --- | --- | --- | --- | --- | --- |
|  | **Age +sex+ FLI** | **Age +sex + FLI + 10-protein score** |  |  | **Age +sex+ FLI** | **Age +sex + FLI + 62-protein score** |  |  |
| Hypertension | 0.701(0.696,0.705) | 0.703(0.699,0.708) | <0.001 | 0.060(0.048,0.072) | 0.701(0.696,0.705) | 0.704(0.7,0.709) | <0.001 | 0.072(0.059,0.086) |
| CHD | 0.712(0.704,0.720) | 0.714(0.706,0.721) | 0.005 | 0.059(0.035,0.080) | 0.712(0.704,0.720) | 0.715(0.707,0.723) | <0.001 | 0.072(0.047,0.088) |
| Stroke | 0.708(0.694,0.721) | 0.708(0.694,0.721) | 0.913 | -0.030(-0.064,0.088) | 0.708(0.694,0.721) | 0.708(0.695,0.721) | 0.768 | 0.008(-0.032,0.039) |
| MASLD | 0.743(0.723,0.763) | 0.775(0.756,0.793) | <0.001 | 0.212(0.151,0.276) | 0.743(0.723,0.763) | 0.786(0.767,0.804) | <0.001 | 0.207(0.135,0.263) |
| Cirrhosis | 0.664(0.640,0.689) | 0.668(0.643,0.693) | 0.232 | 0.102(0.049,0.159) | 0.664(0.640,0.689) | 0.672(0.647,0.696) | 0.061 | 0.079(0.018,0.137) |
| Liver failure | 0.697(0.644,0.750) | 0.717(0.666,0.768) | 0.059 | 0.222(0.112,0.332) | 0.697(0.644,0.750) | 0.723(0.673,0.773) | 0.031 | 0.157(0.03,0.308) |
| Hepatocellular carcinoma | 0.750(0.656,0.843) | 0.755(0.663,0.847) | 0.695 | 0.154(-0.102,0.353) | 0.750(0.656,0.843) | 0.752(0.66,0.843) | 0.900 | 0.039(-0.13,0.270) |
| Gout | 0.815(0.801,0.829) | 0.822(0.808,0.836) | <0.001 | 0.150(0.086,0.185) | 0.815(0.801,0.829) | 0.821(0.808,0.835) | <0.001 | 0.114(0.052,0.157) |
| Kidney stones | 0.631(0.608,0.653) | 0.645(0.623,0.667) | 0.002 | 0.112(0.072,0.153) | 0.631(0.608,0.653) | 0.648(0.626,0.67) | 0.001 | 0.113(0.054,0.163) |
| Chronic kidney disease | 0.749(0.738,0.761) | 0.752(0.741,0.763) | 0.007 | 0.078(0.035,0.110) | 0.749(0.738,0.761) | 0.75(0.739,0.761) | 0.343 | 0.029(-0.048,0.060) |
| Acute kidney injury | 0.721(0.710,0.732) | 0.722(0.711,0.733) | 0.112 | 0.043(-0.001,0.073) | 0.721(0.710,0.732) | 0.723(0.712,0.734) | 0.027 | 0.042(0.004,0.075) |
| Type 2 diabetes | 0.773(0.764,0.781) | 0.807(0.799,0.815) | <0.001 | 0.263(0.236,0.290) | 0.773(0.764,0.781) | 0.812(0.804,0.82) | <0.001 | 0.276(0.248,0.298) |
| COPD | 0.685(0.674,0.695) | 0.685(0.674,0.695) | 0.410 | 0.028(-0.025,0.052) | 0.685(0.674,0.695) | 0.688(0.678,0.699) | 0.004 | 0.065(0.043,0.092) |
| Obstructive sleep apnea | 0.742(0.723,0.761) | 0.744(0.726,0.763) | 0.263 | 0.057(0.002,0.123) | 0.742(0.723,0.761) | 0.744(0.725,0.762) | 0.362 | 0.053(-0.007,0.096) |

**Abbreviations**: LFC, liver fat content; FLI, fatty liver index; COPD, chronic obstructive pulmonary disease; MASLD, metabolic dysfunction-associated steatotic liver disease; CHD, coronary heart disease; NRI, net reclassification improvement.

**Supplementary Table 12. Enhanced predictive performance of the 62- and 10-protein LFC score for 14 cardiovascular-kidney-metabolic (CKM) outcomes beyond standard risk factors using multiple imputation. ***

| **Outcomes** | **C-index** | | ***P***  **value** | **NRI overall** | **C-index** | | ***P***  **value** | **NRI overall** |
| --- | --- | --- | --- | --- | --- | --- | --- | --- |
|  | **Base model** | **Base + 10-protein score** |  |  | **Age +sex+ FLI** | **Age +sex + FLI + 62-protein score** |  |  |
| Hypertension | 0.749(0.745,0.753) | 0.750(0.746,0.754) | <0.001 | 0.078(0.064,0.090) | 0.749(0.745,0.753) | 0.751(0.747,0.755) | <0.001 | 0.080(0.063,0.095) |
| CHD | 0.723(0.715,0.730) | 0.725(0.718,0.733) | <0.001 | 0.071(0.047,0.090) | 0.723(0.715,0.730) | 0.726(0.718,0.733) | <0.001 | 0.082(0.060,0.101) |
| Stroke | 0.726(0.713,0.739) | 0.726(0.713,0.739) | 0.985 | 0.015(-0.041,0.058) | 0.726(0.713,0.739) | 0.726(0.713,0.739) | 0.801 | 0.014(-0.029,0.054) |
| MASLD | 0.726(0.705,0.746) | 0.788(0.770,0.805) | <0.001 | 0.313(0.264,0.382) | 0.726(0.705,0.746) | 0.798(0.781,0.816) | <0.001 | 0.348(0.302,0.399) |
| Cirrhosis | 0.690(0.666,0.713) | 0.701(0.676,0.725) | 0.021 | 0.137(0.075,0.192) | 0.690(0.666,0.713) | 0.702(0.678,0.727) | 0.012 | 0.168(0.088,0.224) |
| Liver failure | 0.732(0.679,0.785) | 0.759(0.708,0.811) | 0.066 | 0.376(0.218,0.485) | 0.732(0.679,0.785) | 0.767(0.717,0.817) | 0.032 | 0.369(0.197,0.486) |
| Hepatocellular carcinoma | 0.766(0.688,0.843) | 0.783(0.703,0.864) | 0.210 | 0.387(0.032,0.582) | 0.766(0.688,0.843) | 0.779(0.698,0.86) | 0.306 | 0.327(0.010,0.543) |
| Gout | 0.815(0.801,0.828) | 0.827(0.814,0.841) | <0.001 | 0.229(0.177,0.269) | 0.815(0.801,0.828) | 0.827(0.814,0.841) | <0.001 | 0.221(0.169,0.283) |
| Kidney stones | 0.649(0.627,0.672) | 0.661(0.640,0.682) | 0.005 | 0.130(0.066,0.182) | 0.649(0.627,0.672) | 0.663(0.641,0.684) | 0.002 | 0.134(0.079,0.165) |
| Chronic kidney disease | 0.768(0.757,0.779) | 0.770(0.760,0.781) | 0.003 | 0.090(0.049,0.125) | 0.768(0.757,0.779) | 0.768(0.758,0.779) | 0.198 | 0.042(-0.028,0.07) |
| Acute kidney injury | 0.747(0.737,0.758) | 0.749(0.738,0.759) | 0.040 | 0.055(0.025,0.086) | 0.747(0.737,0.758) | 0.749(0.739,0.759) | 0.023 | 0.054(0.012,0.089) |
| Type 2 diabetes | 0.841(0.834,0.848) | 0.866(0.859,0.872) | <0.001 | 0.322(0.292,0.350) | 0.841(0.834,0.848) | 0.869(0.863,0.876) | <0.001 | 0.332(0.309,0.358) |
| COPD | 0.790(0.781,0.799) | 0.791(0.782,0.800) | 0.078 | 0.051(0.022,0.078) | 0.790(0.781,0.799) | 0.792(0.783,0.801) | 0.011 | 0.088(0.057,0.109) |
| Obstructive sleep apnea | 0.764(0.745,0.783) | 0.769(0.749,0.788) | 0.061 | 0.156(0.100,0.204) | 0.764(0.745,0.783) | 0.768(0.749,0.787) | 0.041 | 0.140(0.084,0.189) |

*The table compares model discrimination (C-statistics) between baseline models (standard risk factors only) and enhanced models (adding the 62- or 10-protein score), with P values reflecting the significance of predictive improvement. Standard risk factors: age, sex, race, BMI, Townsend deprivation index, smoking status, alcohol use, systolic blood pressure, LDL cholesterol, and HbA1c.

**Abbreviations**: LFC, liver fat content; FLI, fatty liver index; COPD, chronic obstructive pulmonary disease; MASLD, metabolic dysfunction-associated steatotic liver disease; CHD, coronary heart disease; NRI, net reclassification improvement.

**Supplementary Table 13. Enhanced predictive performance of the 62- and 10-protein LFC score for 14 cardiovascular-kidney-metabolic (CKM) outcomes beyond FLI: Analysis of Complete Cases (Sample size: 41,213 for the 10-protein score and 26,168 for the 62-protein score).**

| **Outcomes** | **C-index** | | ***P***  **value** | **NRI overall** | **C-index** | | ***P***  **value** | **NRI overall** |
| --- | --- | --- | --- | --- | --- | --- | --- | --- |
|  | **Age +sex+ FLI** | **Age +sex + FLI + 10-protein score** |  |  | **Age +sex+ FLI** | **Age +sex + FLI + 62-protein score** |  |  |
| Hypertension | 0.701(0.697,0.706) | 0.704(0.699,0.709) | <0.001 | 0.062(0.051,0.075) | 0.698(0.692,0.704) | 0.701(0.695,0.707) | <0.001 | 0.071(0.050,0.091) |
| CHD | 0.712(0.704,0.72) | 0.714(0.706,0.722) | 0.013 | 0.056(0.026,0.081) | 0.708(0.698,0.718) | 0.710(0.700,0.721) | 0.007 | 0.065(0.036,0.09) |
| Stroke | 0.707(0.693,0.721) | 0.707(0.693,0.721) | 0.870 | -0.039(-0.067,0.073) | 0.706(0.689,0.723) | 0.706(0.689,0.723) | 0.981 | -0.005(-0.026,0.059) |
| MASLD | 0.743(0.722,0.764) | 0.775(0.755,0.795) | <0.001 | 0.214(0.164,0.282) | 0.750(0.725,0.776) | 0.797(0.773,0.821) | <0.001 | 0.228(0.124,0.315) |
| Cirrhosis | 0.662(0.635,0.689) | 0.666(0.639,0.692) | 0.258 | 0.091(0.031,0.153) | 0.661(0.629,0.693) | 0.671(0.640,0.703) | 0.039 | 0.054(-0.018,0.128) |
| Liver failure | 0.684(0.625,0.744) | 0.709(0.652,0.766) | 0.133 | 0.243(0.069,0.395) | 0.673(0.600,0.747) | 0.719(0.653,0.785) | 0.037 | 0.240(0.088,0.445) |
| Hepatocellular carcinoma | 0.760(0.657,0.862) | 0.760(0.659,0.861) | 0.978 | 0.156(-0.235,0.425) | 0.697(0.509,0.886) | 0.698(0.505,0.890) | 0.996 | -0.096(-0.36,0.423) |
| Gout | 0.815(0.800,0.829) | 0.822(0.807,0.836) | 0.003 | 0.145(0.083,0.205) | 0.814(0.795,0.832) | 0.823(0.805,0.841) | 0.001 | 0.141(0.073,0.218) |
| Kidney stones | 0.624(0.601,0.648) | 0.642(0.620,0.665) | 0.001 | 0.118(0.061,0.169) | 0.611(0.581,0.641) | 0.630(0.601,0.660) | 0.022 | 0.117(0.048,0.184) |
| Chronic kidney disease | 0.749(0.737,0.761) | 0.752(0.740,0.764) | 0.010 | 0.084(0.043,0.110) | 0.745(0.729,0.761) | 0.746(0.730,0.761) | 0.349 | 0.043(-0.009,0.085) |
| Acute kidney injury | 0.720(0.709,0.732) | 0.722(0.711,0.734) | 0.016 | 0.047(0.007,0.081) | 0.714(0.699,0.729) | 0.718(0.704,0.733) | 0.005 | 0.047(0.009,0.086) |
| Type 2 diabetes | 0.775(0.766,0.783) | 0.808(0.800,0.816) | <0.001 | 0.260(0.238,0.282) | 0.775(0.765,0.786) | 0.814(0.804,0.824) | <0.001 | 0.278(0.247,0.305) |
| COPD | 0.686(0.674,0.697) | 0.686(0.675,0.697) | 0.423 | 0.031(-0.024,0.058) | 0.675(0.661,0.689) | 0.681(0.667,0.695) | 0.006 | 0.084(0.048,0.116) |
| Obstructive sleep apnea | 0.743(0.724,0.763) | 0.746(0.726,0.765) | 0.193 | 0.047(-0.003,0.103) | 0.753(0.728,0.777) | 0.753(0.729,0.778) | 0.662 | 0.059(-0.012,0.115) |

**Abbreviations**: LFC, liver fat content; FLI, fatty liver index; COPD, chronic obstructive pulmonary disease; MASLD, metabolic dysfunction-associated steatotic liver disease; CHD, coronary heart disease; NRI, net reclassification improvement.

**Supplementary Table 14. Enhanced predictive performance of the 62- and 10-protein LFC score for 14 cardiovascular-kidney-metabolic (CKM) outcomes beyond standard risk factors: Analysis of Complete Cases (Sample size: 41,213 for the 10-protein score and 26,168 for the 62-protein score). ***

| **Outcomes** | **C-index** | | ***P***  **value** | **NRI overall** | **C-index** | | ***P***  **value** | **NRI overall** |
| --- | --- | --- | --- | --- | --- | --- | --- | --- |
|  | **Base model** | **Base + 10-protein score** |  |  | **Age +sex+ FLI** | **Age +sex + FLI + 62-protein score** |  |  |
| Hypertension | 0.750(0.745,0.754) | 0.751(0.747,0.755) | <0.001 | 0.084(0.069,0.098) | 0.749(0.743,0.754) | 0.750(0.745,0.756) | <0.001 | 0.086(0.064,0.109) |
| CHD | 0.723(0.715,0.731) | 0.725(0.717,0.733) | 0.001 | 0.072(0.048,0.096) | 0.722(0.712,0.731) | 0.724(0.714,0.734) | 0.002 | 0.075(0.055,0.102) |
| Stroke | 0.725(0.711,0.738) | 0.725(0.711,0.738) | 0.918 | 0.017(-0.046,0.050) | 0.727(0.711,0.744) | 0.727(0.711,0.744) | 0.752 | -0.006(-0.049,0.074) |
| MASLD | 0.727(0.706,0.748) | 0.791(0.772,0.809) | <0.001 | 0.295(0.245,0.354) | 0.733(0.707,0.760) | 0.812(0.790,0.835) | <0.001 | 0.339(0.259,0.430) |
| Cirrhosis | 0.689(0.664,0.714) | 0.701(0.675,0.727) | 0.026 | 0.144(0.067,0.220) | 0.698(0.668,0.728) | 0.713(0.683,0.744) | 0.009 | 0.140(0.042,0.220) |
| Liver failure | 0.725(0.667,0.782) | 0.765(0.711,0.820) | 0.012 | 0.367(0.202,0.516) | 0.739(0.670,0.808) | 0.790(0.725,0.856) | 0.005 | 0.527(0.301,0.636) |
| Hepatocellular carcinoma | 0.780(0.699,0.861) | 0.790(0.700,0.879) | 0.394 | 0.382(0.007,0.607) | 0.854(0.766,0.941) | 0.854(0.766,0.941) | 0.976 | -0.17(-0.282,0.459) |
| Gout | 0.813(0.798,0.827) | 0.826(0.812,0.840) | <0.001 | 0.220(0.151,0.269) | 0.814(0.795,0.832) | 0.829(0.811,0.846) | <0.001 | 0.239(0.164,0.308) |
| Kidney stones | 0.647(0.624,0.670) | 0.661(0.639,0.684) | 0.002 | 0.139(0.088,0.186) | 0.650(0.621,0.680) | 0.664(0.635,0.693) | 0.033 | 0.118(0.034,0.177) |
| Chronic kidney disease | 0.769(0.757,0.780) | 0.772(0.760,0.783) | 0.002 | 0.104(0.062,0.131) | 0.764(0.749,0.780) | 0.765(0.749,0.780) | 0.355 | 0.067(-0.059,0.112) |
| Acute kidney injury | 0.747(0.736,0.758) | 0.749(0.738,0.760) | 0.007 | 0.066(0.028,0.101) | 0.744(0.730,0.758) | 0.747(0.733,0.761) | 0.028 | 0.083(0.037,0.124) |
| Type 2 diabetes | 0.841(0.833,0.849) | 0.866(0.859,0.873) | <0.001 | 0.323(0.293,0.344) | 0.856(0.847,0.865) | 0.881(0.873,0.889) | <0.001 | 0.324(0.296,0.364) |
| COPD | 0.788(0.778,0.797) | 0.789(0.779,0.798) | 0.097 | 0.051(0.020,0.078) | 0.786(0.774,0.799) | 0.789(0.777,0.801) | 0.028 | 0.097(0.067,0.137) |
| Obstructive sleep apnea | 0.763(0.743,0.783) | 0.767(0.748,0.787) | 0.029 | 0.157(0.086,0.198) | 0.774(0.749,0.799) | 0.776(0.750,0.801) | 0.363 | 0.129(0.046,0.183) |

*The table compares model discrimination (C-statistics) between baseline models (standard risk factors only) and enhanced models (adding the 62- or 10-protein score), with P values reflecting the significance of predictive improvement. Standard risk factors: age, sex, race, BMI, Townsend deprivation index, smoking status, alcohol use, systolic blood pressure, LDL cholesterol, and HbA1c.

**Abbreviations**: LFC, liver fat content; FLI, fatty liver index; COPD, chronic obstructive pulmonary disease; MASLD, metabolic dysfunction-associated steatotic liver disease; CHD, coronary heart disease; NRI, net reclassification improvement.

**Supplementary Table 15. Predictive performance of protein panels versus FLI for various disease outcomes.**

| **Outcomes** | **AUC (95% CI): 62-Protein Panel** | **AUC (95% CI): 10-Protein Panel** | **AUC (95% CI): FLI** | ***P* value: 62-Protein Panel vs FLI** | ***P* value: 10-Protein Panel vs FLI** | **NRI (95% CI): 62-Protein Panel vs FLI** | **NRI (95% CI): 10-Protein Panel vs FLI** |
| --- | --- | --- | --- | --- | --- | --- | --- |
| Hypertension | 0.740 (0.736-0.744) | 0.738 (0.733-0.742) | 0.743 (0.739-0.747) | 0.002 | <0.001 | -0.062(-0.080, -0.045) | -0.065(-0.085, -0.043) |
| CHD | 0.738 (0.732-0.743) | 0.737 (0.731-0.743) | 0.739 (0.733-0.744) | 0.282 | 0.057 | -0.04(-0.069, -0.002) | -0.035(-0.063, -0.006) |
| Stroke | 0.698 (0.688-0.708) | 0.698 (0.688-0.708) | 0.699 (0.689-0.709) | 0.075 | 0.073 | -0.043(-0.091, -0.005) | -0.035(-0.076,0.007) |
| MASLD | 0.780 (0.765-0.795) | 0.768 (0.753-0.783) | 0.741 (0.725-0.757) | <0.001 | <0.001 | 0.133(0.050,0.197) | 0.087(0.013,0.148) |
| Cirrhosis | 0.668 (0.649-0.687) | 0.666 (0.647-0.686) | 0.668 (0.649-0.687) | 0.971 | 0.766 | -0.007(-0.084,0.072) | -0.002(-0.100,0.094) |
| Liver failure | 0.710 (0.670-0.750) | 0.710 (0.670-0.749) | 0.699 (0.658-0.740) | 0.351 | 0.385 | 0.055(-0.146,0.295) | 0.054(-0.136,0.248) |
| Hepatocellular carcinoma | 0.723 (0.653-0.793) | 0.723 (0.652-0.794) | 0.725 (0.654-0.796) | 0.882 | 0.890 | -0.163(-0.335, -0.218) | -0.067(-0.327,0.134) |
| Gout | 0.803 (0.793-0.813) | 0.807 (0.797-0.817) | 0.810 (0.800-0.820) | 0.014 | 0.280 | -0.205(-0.263, -0.148) | -0.137(-0.198, -0.072) |
| Kidney stones | 0.648 (0.633-0.662) | 0.645 (0.631-0.659) | 0.639 (0.624-0.653) | 0.003 | 0.036 | 0.047(0.002,0.109) | 0.030(-0.009,0.074) |
| Chronic kidney disease | 0.742 (0.734-0.750) | 0.750 (0.742-0.758) | 0.753 (0.745-0.762) | <0.001 | 0.030 | -0.178(-0.205, -0.150) | -0.064(-0.105, -0.011) |
| Acute kidney injury | 0.722 (0.714-0.730) | 0.723 (0.715-0.731) | 0.729 (0.721-0.737) | <0.001 | <0.001 | -0.126(-0.149, -0.085) | -0.089(-0.120, -0.053) |
| Type 2 diabetes | 0.803 (0.797-0.810) | 0.799 (0.793-0.805) | 0.778 (0.772-0.784) | <0.001 | <0.001 | 0.097(0.063, 0.129) | 0.087(0.053, 0.121) |
| COPD | 0.691 (0.683-0.699) | 0.686 (0.677-0.694) | 0.689 (0.681-0.697) | 0.166 | 0.015 | 0.004(-0.027, 0.040) | -0.05(-0.085, -0.010) |
| Obstructive sleep apnea | 0.728 (0.714-0.742) | 0.727 (0.713-0.741) | 0.750 (0.736-0.764) | <0.001 | <0.001 | -0.177(-0.226, -0.126) | -0.163(-0.215, -0.105) |

**Note:** This analysis was conducted among 53,017 participants in the full proteomic cohort. *P*-values from DeLong's test compare each protein model against PDFF as reference. NRI values >0 indicate improved risk reclassification.

**Abbreviations:** CHD, coronary heart disease; MASLD, metabolic dysfunction-associated steatotic liver disease; FLI, fatty liver index; COPD, chronic obstructive pulmonary disease; LFC, liver fat content; AUC, area under the curve; NRI, net reclassification improvement.

**Supplementary Table 16. Predictive performance of protein panels versus PDFF for various disease outcomes.**

| **Outcomes** | **AUC (95% CI): 62-Protein Panel** | **AUC (95% CI): 10-Protein Panel** | **AUC (95% CI): PDFF** | ***P* value: 62-Protein Panel vs PDFF** | ***P* value: 10-Protein Panel vs PDFF** | **NRI (95% CI): 62-Protein Panel vs PDFF** | **NRI (95% CI): 10-Protein Panel vs PDFF** |
| --- | --- | --- | --- | --- | --- | --- | --- |
| Hypertension | 0.752(0.737-0.768) | 0.751(0.735-0.766) | 0.724(0.708-0.740) | <0.001 | <0.001 | 0.204(0.156,0.247) | 0.217(0.163,0.253) |
| CHD | 0.729(0.704-0.753) | 0.727(0.703-0.752) | 0.716(0.691-0.740) | 0.003 | 0.005 | 0.182(0.106,0.233) | 0.176(0.123,0.238) |
| Stroke | 0.697(0.643-0.750) | 0.696(0.642-0.750) | 0.690(0.636-0.744) | 0.317 | 0.348 | 0.127(-0.064,0.266) | 0.170(-0.109,0.33) |
| MASLD | 0.840(0.780-0.900) | 0.824(0.763-0.884) | 0.810(0.743-0.877) | 0.372 | 0.718 | 0.318(0.043,0.605) | 0.283(0.010,0.570) |
| Cirrhosis | 0.604(0.478-0.730) | 0.599(0.474-0.723) | 0.615(0.490-0.740) | 0.793 | 0.626 | -0.151(-0.417,0.433) | -0.232(-0.347,0.33) |
| Gout | 0.836(0.799-0.872) | 0.833(0.796-0.870) | 0.824(0.787-0.862) | 0.314 | 0.484 | 0.272(0.117,0.456) | 0.255(0.059,0.424) |
| Kidney stones | 0.699(0.648-0.750) | 0.695(0.644-0.747) | 0.695(0.645-0.746) | 0.724 | 0.969 | 0.032(-0.126,0.136) | -0.011(-0.175,0.129) |
| Chronic kidney disease | 0.730(0.691-0.769) | 0.746(0.708-0.784) | 0.711(0.669-0.753) | 0.042 | 0.005 | 0.225(0.102,0.335) | 0.289(0.174,0.385) |
| Acute kidney injury | 0.696(0.649-0.742) | 0.698(0.651-0.744) | 0.687(0.640-0.735) | 0.165 | 0.136 | 0.184(-0.014,0.324) | 0.228(-0.018,0.353) |
| Type 2 diabetes | 0.837(0.810-0.865) | 0.830(0.802-0.857) | 0.769(0.739-0.799) | <0.001 | <0.001 | 0.378(0.290,0.461) | 0.369(0.277,0.446) |
| COPD | 0.678(0.632-0.725) | 0.675(0.628-0.722) | 0.671(0.623-0.718) | 0.319 | 0.512 | 0.129(-0.108,0.268) | 0.135(-0.17,0.264) |
| Obstructive sleep apnea | 0.775(0.724-0.826) | 0.775(0.725-0.825) | 0.751(0.700-0.803) | 0.110 | 0.108 | 0.241(0.088,0.414) | 0.232(0.018,0.380) |

**Note:** This analysis was conducted among 5,320 participants who had both PDFF measurements and protein scores available. Results for liver failure (n=6) and hepatocellular carcinoma (n=3) are not shown due to small sample sizes. *P*-values from DeLong's test compare each protein model against PDFF as reference. NRI values >0 indicate improved risk reclassification.

**Abbreviations:** CHD, coronary heart disease; MASLD, metabolic dysfunction-associated steatotic liver disease; COPD, chronic obstructive pulmonary disease; LFC, liver fat content; AUC, area under the curve; PDFF, proton density fat fraction; NRI, net reclassification improvement.

**Supplementary Table 17. Cox model results from UK Biobank with protein scores and polygenic risk scores as the main predictors, including an interaction term.***

| **Exposure** | **PRS** | **Protein: HR (95% CI)** | **PRS: HR**  **(95% CI)** | **Interaction: HR (95% CI)** | **P for interaction** |
| --- | --- | --- | --- | --- | --- |
| The 62-protein LFC score | Hypertension PRS | 1.48(1.45,1.50) | 1.35(1.33,1.38) | 0.99(0.97,1.00) | 0.104 |
|  | CHD PRS | 1.31(1.26,1.35) | 1.35(1.31,1.40) | 1.00(0.97,1.04) | 0.769 |
|  | Type 2 diabetes PRS | 2.95(2.82,3.09) | 1.87(1.77,1.98) | 0.91(0.88,0.94) | <0.001 |
| The 10-protein LFC score | Hypertension PRS | 1.44(1.42,1.47) | 1.35(1.33,1.38) | 0.99(0.97,1.01) | 0.302 |
|  | CHD PRS | 1.28(1.24,1.32) | 1.35(1.31,1.40) | 1.00(0.97,1.04) | 0.886 |
|  | Type 2 diabetes PRS | 2.85(2.73,2.98) | 1.89(1.79,2.00) | 0.91(0.87,0.94) | <0.001 |

*Adjusted for age, sex, race and four principal components of genetic ancestry (UKB Field 26201).

**Abbreviations:** LFC, liver fat content; PRS, polygenic risk scores; CHD, coronary heart disease.

**Supplementary Table 18. Definitions of relevant UKB data fields and codes.**

| **Variables** | **Field ID** | **Field names** |
| --- | --- | --- |
| **PDFF** | 40061 | Proton density fat fraction (PDFF) |
| **Polygenic risk score (PRS)** |  |  |
| PRS for hypertension | 26244 | Standard PRS for hypertension (HT) |
| PRS for CHD | 26227 | Standard PRS for coronary artery disease (CAD) |
| PRS for type 2 diabetes | 26285 | Standard PRS for type 2 diabetes (T2D) |
| **Covariates** |  |  |
| Age | 21022 | Age at recruitment |
| Sex | 31 | Sex |
| Race | 21000 | Ethnic background |
| Weight | 21002 | Weight |
| Height | 50 | Standing height |
| Townsend deprivation index | 22189 | Townsend deprivation index at recruitment |
| Systolic blood pressure | 4080; 93 | Systolic blood pressure, automated reading; Systolic blood pressure, manual reading |
| Smoking status | 20116 | Smoking status |
| Alcohol consumption | 1558 | Alcohol intake frequency |
| Low-density lipoprotein cholesterol (LDL) | 30780 | LDL direct |
| HbA1c | 30750 | Glycated haemoglobin (HbA1c) |
| Four principal components of genetic ancestry | 26201 | PRS genetic principal components |

**Details about the collection of these covariates is available in the UK Biobank online protocol (www.ukbiobank.ac.uk)**.

**Supplementary Table 19. MAR Assumption Evaluation Results. ***

| **Protein** | **Missing Rate (%)** | **Number of Significant Associations** | **Key Associated Variables** |
| --- | --- | --- | --- |
| C3 | 19.7 | 4 | Race, TDI, Alcohol, LDL-C |
| KLK7 | 19.5 | 5 | Race, TDI, Smoking, LDL-C, Systolic blood pressure |
| GCHFR | 19.4 | 6 | Sex, Race, TDI, Smoking, LDL-C, Systolic blood pressure |
| NHLRC3 | 19.4 | 5 | Race, TDI, Smoking, LDL-C, Systolic blood pressure |
| APOD | 19.2 | 6 | Age, Race, TDI, Smoking, LDL-C, Systolic blood pressure |
| GAPDH | 19.2 | 6 | Sex, Race, TDI, Smoking, LDL-C, Systolic blood pressure |
| TP53I3 | 19.2 | 5 | Race, TDI, Smoking, LDL-C, Systolic blood pressure |
| CPA4 | 19.0 | 5 | Race, TDI, Smoking, LDL-C, Systolic blood pressure |
| ANXA2 | 19.0 | 3 | Race, TDI, Smoking |
| GRSF1 | 19.0 | 3 | Race, TDI, Smoking |

*All proteins showed evidence supporting the MAR assumption (≥3 significant associations with clinical variables including age, sex, race, TDI, BMI, smoking status, alcohol consumption, LDL-C, systolic blood pressure, and HbA1c). Associations between protein missingness (missing vs observed) and clinical variables were tested using t-tests for continuous variables and chi-square/Fisher's exact tests for categorical variables.

**Abbreviations:** MAR, missing at random; TDI, Townsend deprivation index; LDL-C, low-density lipoprotein cholesterol;

**Supplementary Table 20. Disease definitions used in the UK Biobank study.**

| **Disease** | **ICD-10** | **ICD-9** | **Opcs-4** | **Reference (DOI)** |
| --- | --- | --- | --- | --- |
| Gout | M10 | 274 |  | 10.1186/s13075-017-1390-1 |
| Kidney stones | N20, N23 | 592, 788 | M09, M14, M26, M27, M28, M31 | 10.1016/j.euf.2019.05.002 |
| Acute kidney injury | I120, I139, K767, N055, N056, N059, N139, N170, N171, N172, N178, N179, N19, N990, P960, R392, T795 |  |  | [10.1007/s40620-021-01174-z](https://doi.org/10.1007/s40620-021-01174-z) |
| Type 2 diabetes | E11 |  |  | 10.1371/journal.pmed.1003767 |
| Chronic kidney disease | I120, I131, I132, N180, N183, N184, N185, N188, N189 | 585 | M01 | 10.1016/j.jacc.2021.05.004 |
| Hypertension | I10 | 401 |  | 10.3389/fcvm.2021.769130 |
| Coronary heart disease | I20, I21, I22, I23, I24, I25 | 410, 411, 412, 413, 414 | K40, K41, K42, K43, K44, K45, K46, K49, K50, K75 | 10.1161/CIRCULATIONAHA.117.032432;  10.1016/j.jacc.2021.05.004 |
| Stroke | I60, I61, I63, I64, I629, I678, I690, I693 | 430, 431, 434, 436 | A052, A053, A054, L351, L353, L343 | 10.1371/journal.pmed.1003830 |
| MASLD | K760, K758 | 5718 |  | 10.1002/hep.31726 |
| Cirrhosis | K746, I859, I982, I864, I850, I983, R18, K767, K766 | 5715, 4561, 45621, 4560, 45620, 7895, 5722, 5724, 5723 |  | 10.1002/hep.31726 |
| Liver failure | K721, K729, K720 | 5728, 570 |  | 10.1002/hep.31726 |
| Hepatocellular carcinoma | C220 | 1550 |  | 10.1002/hep.31726 |
| COPD | J40, J41, J42, J43, J44 | 490,491,492,496 |  | 10.1016/j.envint.2022.107654 |
| Obstructive sleep apnea | G473 |  |  | 10.1038/s41366-023-01402-5 |

**Abbreviations**: ICD, International Classification of Diseases, OPCS, Office of Population Censuses and Surveys: Classification of Intervention and Procedures; COPD, chronic obstructive pulmonary disease; BMI, body mass index; MASLD, metabolic dysfunction-associated steatotic liver disease.
